# A Secreted Bacterial Peptidylarginine Deiminase Can Neutralize Human Innate Immune Defenses

**DOI:** 10.1128/mBio.01704-18

**Published:** 2018-10-30

**Authors:** Tim Stobernack, Marines du Teil Espina, Lianne M. Mulder, Laura M. Palma Medina, Dillon R. Piebenga, Giorgio Gabarrini, Xin Zhao, Koen M. J. Janssen, Jarnick Hulzebos, Elisabeth Brouwer, Thomas Sura, Dörte Becher, Arie Jan van Winkelhoff, Friedrich Götz, Andreas Otto, Johanna Westra, Jan Maarten van Dijl

**Affiliations:** a Department of Medical Microbiology, University of Groningen, University Medical Center Groningen, Groningen, The Netherlands; b Department of Periodontology, University of Groningen, University Medical Center Groningen, Center for Dentistry and Oral Hygiene, Groningen, The Netherlands; c Department of Oral and Maxillofacial Surgery, University of Groningen, University Medical Center Groningen, Groningen, The Netherlands; d Department of Rheumatology and Clinical Immunology, University of Groningen, University Medical Center Groningen, Groningen, The Netherlands; e Institute for Microbiology, Ernst-Moritz-Arndt-University Greifswald, Greifswald, Germany; f Microbial Genetics, Interfaculty Institute of Microbiology and Infection Medicine and Infection Medicine (IMIT), University of Tübingen, Tübingen, Germany; GSK Vaccines

**Keywords:** Porphyromonas gingivalis, citrullination, immune evasion, neutrophils, protein modification

## Abstract

The keystone oral pathogen Porphyromonas gingivalis is associated with severe periodontitis. Intriguingly, this bacterium is known to secrete large amounts of an enzyme that converts peptidylarginine into citrulline residues. The present study was aimed at identifying possible functions of this citrullinating enzyme, named Porphyromonas peptidylarginine deiminase (PPAD), in the periodontal environment. The results show that PPAD is detectable in the gingiva of patients with periodontitis, and that it literally neutralizes human innate immune defenses at three distinct levels, namely bacterial phagocytosis, capture in neutrophil extracellular traps (NETs), and killing by the lysozyme-derived cationic antimicrobial peptide LP9. As shown by mass spectrometry, exposure of neutrophils to PPAD-proficient bacteria reduces the levels of neutrophil proteins involved in phagocytosis and the bactericidal histone H2. Further, PPAD is shown to citrullinate the histone H3, thereby facilitating the bacterial escape from NETs. Last, PPAD is shown to citrullinate LP9, thereby restricting its antimicrobial activity. The importance of PPAD for immune evasion is corroborated in the infection model Galleria mellonella, which only possesses an innate immune system. Together, the present observations show that PPAD-catalyzed protein citrullination defuses innate immune responses in the oral cavity, and that the citrullinating enzyme of P. gingivalis represents a new type of bacterial immune evasion factor.

## Abstract (precis)

Bacterial pathogens do not only succeed in breaking the barriers that protect humans from infection, but they also manage to evade insults from the human immune system. The importance of the present study resides in the fact that protein citrullination is shown to represent a new bacterial mechanism for immune evasion. In particular, the oral pathogen P. gingivalis employs this mechanism to defuse innate immune responses by secreting a protein-citrullinating enzyme. Of note, this finding impacts not only the global health problem of periodontitis, but it also extends to the prevalent autoimmune disease rheumatoid arthritis, which has been strongly associated with periodontitis, PPAD activity, and loss of tolerance against citrullinated proteins, such as the histone H3.

## INTRODUCTION

**FIG 1:**
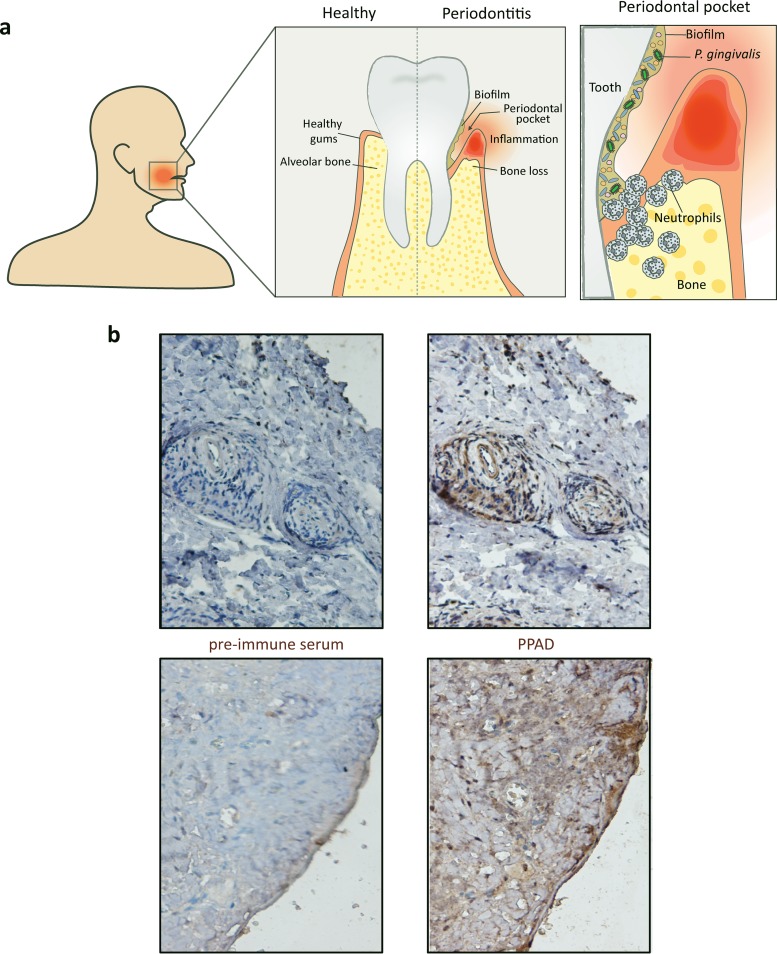
Detection of PPAD in gingival tissue of a periodontitis patient. (a) Hallmarks of periodontitis, with schematic representation of biofilm formation and neutrophil recruitment in the periodontal pocket. Note that the periodontal biofilm is polymicrobial, where *P. gingivalis* is represented in green and other microorganisms in orange and blue. (b) PPAD detection by immunohistochemistry in gingival tissues of a periodontitis patient using a PPAD-specific antibody. Control staining of the same gingival tissues was performed with the respective rabbit preimmune serum. PPAD staining is observed in gingival tissue primarily around blood vessels (upper images) or at the epithelium (lower images).

Periodontitis affects around 10% to 15% of the adult population, making it one of the most prevalent diseases worldwide (1). It is characterized by chronic inflammation of the tissues supporting the teeth and is associated with a dysbiotic oral microbiome found primarily in the form of biofilms in the periodontal pocket (Fig. 1a). These conditions trigger an increased tissue infiltration by immune cells, mainly neutrophils, which play a pivotal role in maintaining periodontal health by employing diverse and potent bactericidal mechanisms (2, 3). Successful periodontal pathogens, however, have evolved sophisticated strategies to avoid or subvert neutrophil killing and to thrive in an inflamed environment. In particular, the Gram-negative anaerobe Porphyromonas gingivalis, which is considered a major etiological agent of periodontitis, possesses the ability to dysregulate the homeostasis between oral biofilms and innate immunity (2, 3). The bacterium secretes large amounts of a unique enzyme, the P. gingivalis peptidylarginine deiminase (PPAD), which catalyzes the citrullination of both bacterial and host proteins (4–8). This posttranslational protein modification involves the deimination of positively charged arginine residues into neutral citrulline residues. Intriguingly, P. gingivalis has not only been implicated in periodontitis but also in the prevalent autoimmune disease rheumatoid arthritis, which is strongly associated with periodontitis, PPAD activity, and a loss of tolerance against citrullinated proteins, such as the histone H3 (2, 9–11). Nonetheless, the biological and clinical relevance of PPAD for dysbiosis in the oral cavity had so far remained enigmatic. The question raised in our present study was whether this citrullinating enzyme may literally neutralize human innate immune defenses in the periodontal environment, thereby serving as a secreted bacterial immune evasion factor.

## RESULTS AND DISCUSSION

### PPAD impairs bacterial binding and internalization by neutrophils.

**FIG 2:**
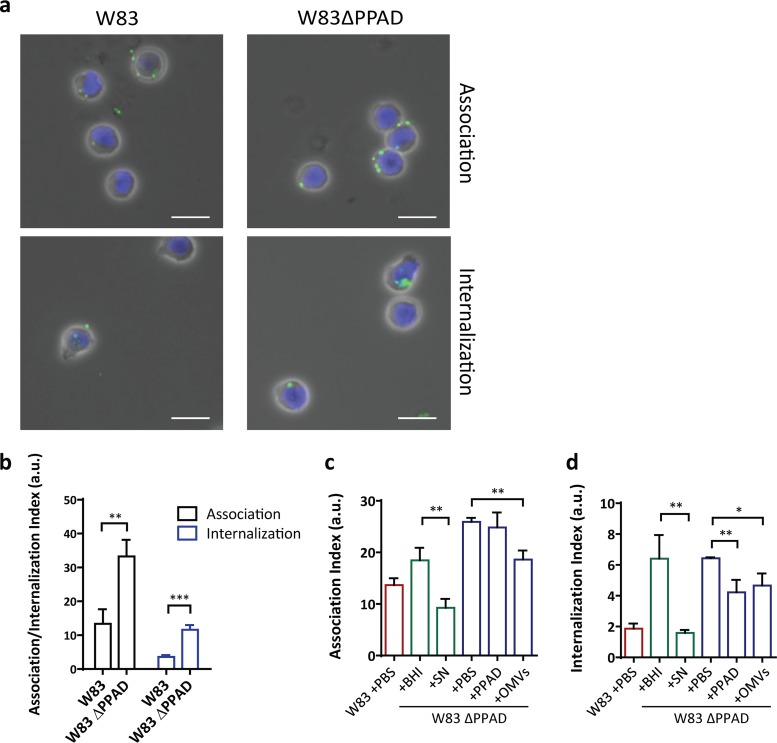
PPAD impairs bacterial binding and internalization by neutrophils. (a and b) *P. gingivalis* W83 ΔPPAD is bound and internalized by neutrophils at a higher rate than wild-type *P. gingivalis* W83. Microscopic visualization of neutrophils with bound or internalized *P. gingivalis* (a) (scale bars = 10 µm), and the respective association and internalization indices as determined by flow cytometry (b). (c and d) Rescue of bacterial binding and internalization by neutrophils upon addition of 2.5 µg recombinant PPAD (indicated as PPAD), 16 µg PPAD-containing W83 outer membrane vesicles (OMVs), or 100 µl PPAD-containing W83 culture supernatant (SN). Association and internalization indices determined by flow cytometry are shown. (b) Data are means of three biologically independent samples (neutrophils from three donors), where each infection experiment was carried out four times. (c and d) Data are means of four replicates of one biological sample (one neutrophil donor). *, *P < *0.05; **, *P < *0.01; ***, *P < *0.001; two-tailed unpaired Student’s *t* tests. Data are presented as mean values ± standard deviation (SD). a.u., arbitrary units.

To verify the relevance of PPAD production in inflamed periodontal tissue, we performed immunohistochemistry using a previously developed PPAD-specific antibody. As shown in Fig. 1b, this allowed us to detect the presence of PPAD in gingival tissues of periodontitis patients for the first time. This observation enticed us to further investigate the interaction of P. gingivalis with key host immune cells. In particular, we aimed this investigation at dissecting potentially pleiotropic functions of PPAD in the evasion of neutrophil-specific innate immunity by P. gingivalis W83, previously characterized as one of the most virulent Porphyromonas strains (12). Challenge with human neutrophils showed that strain W83 is bound and internalized by these neutrophils (Fig. 2a and b). Notably, the association and internalization levels observed for a genetically engineered PPAD-deficient P. gingivalis mutant were 2- to 3-fold higher than in the parental W83 strain (Fig. 2b). This is partly related to a higher percentage of the neutrophils binding and internalizing PPAD-deficient P. gingivalis (Fig. S1a). The addition of PPAD-containing culture supernatant allowed the PPAD-deficient mutant to evade neutrophil association and internalization, and significant evasion of neutrophil internalization was even observed upon the addition of purified recombinant PPAD (Fig. 2c and d). This shows that PPAD helps P. gingivalis evade destruction by neutrophils, which is a prerequisite to survive the high neutrophil influx in inflamed gingival tissue of periodontitis patients.

10.1128/mBio.01704-18.1 FIG S1 Supporting data for the neutrophil association (assoc.) and invasion assays. (a) P. gingivalis W83 ΔPPAD is bound and internalized (intern.) by neutrophils at a higher rate than wild-type P. gingivalis W83. (b) Western blot detection of PPAD in W83 OMVs and absence of PPAD from W83 ΔPPAD OMVs. (c) Western blot detection of outer membrane protein 41 (Omp41) in W83 and W83 ΔPPAD OMVs. (d) Levels of the gingipains RgpA and RgpB are lower in neutrophils infected with wild-type P. gingivalis W83 than in the W83 ΔPPAD strain, as determined by Western blotting, corresponding to Fig. 1g; a representative image of three independent experiments is shown. (e to g) Flow cytometry gating strategy for inclusion of human neutrophils based on forward scatter (FSC-H) and side scatter (SSC-H). (h to j) FITC-fluorescence peaks of uninfected (h) and FITC-stained P. gingivalis-infected neutrophils (i and j). V1-L represents the FITC-negative cell population, and V1-R represents the FITC-positive cell population. (e to j) Images shown in are representative for the respective infection condition. Data are presented as mean values ± SD. *, *P < *0.05; **, *P* < 0.01, two-tailed unpaired Student’s *t* tests. Download FIG S1, PDF file, 1.5 MB. Copyright © 2018 Stobernack et al. 2018 Stobernack et al. This content is distributed under the terms of the Creative Commons Attribution 4.0 International license.

We have recently shown that PPAD is secreted in two different forms, either in a soluble state or bound to excreted outer membrane vesicles (OMVs) (7, 8). As shown with the recombinant protein, soluble PPAD can limit neutrophil internalization, and the same effect was observed upon addition of purified PPAD-containing OMVs to the PPAD-deficient P. gingivalis (Fig. 2d; see also Fig. S1b and c in the supplemental material). Moreover, these OMVs even inhibited binding of the PPAD mutant bacteria by neutrophils (Fig. 2c). Together, these observations imply that both forms of secreted PPAD, soluble and OMV bound, can serve to protect P. gingivalis against containment and elimination by human neutrophils. Further, the data suggest that OMV-bound PPAD could be primarily used by P. gingivalis to evade neutrophil binding, while the soluble PPAD might be more effective against internalization. However, it is important to bear in mind that the recombinant PPAD isolated from Lactococcus lactis, though soluble and enzymatically active, may lack particular as-yet-unidentified posttranslational modifications that are present in the soluble PPAD produced by P. gingivalis. Such modifications could impact, for example, the enzyme’s substrate specificity and specific activity. This awaits further experimental verification by purification of soluble PPAD from the P. gingivalis W83 growth medium and subsequent functional and structural characterization.

**FIG 3:**
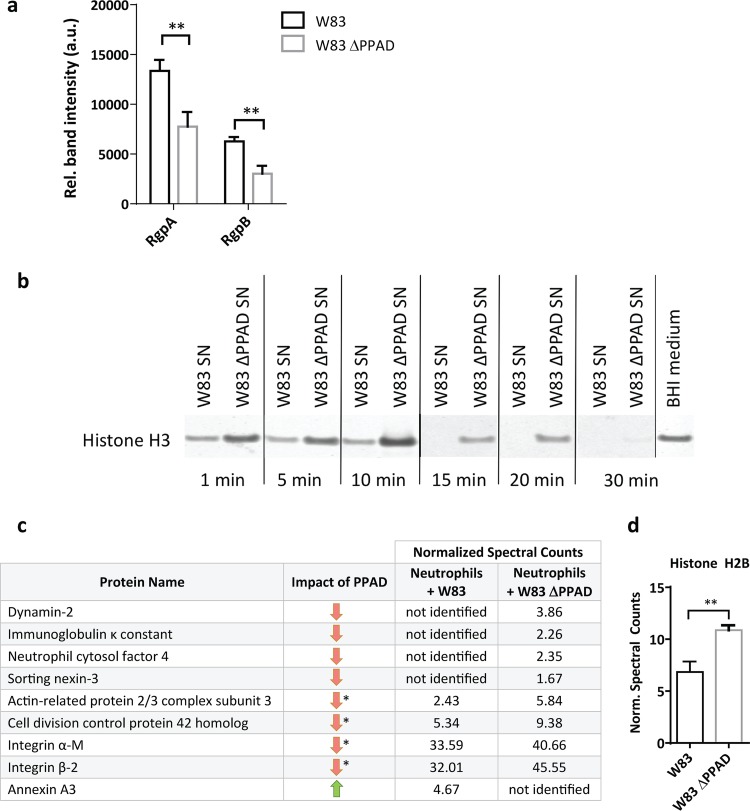
PPAD stabilizes gingipains and modulates the levels of phagocytosis-related proteins. (a) Relative (Rel.) levels of gingipains (RgpA/RgpB) in infected neutrophils. (b) Time course of histone H3 degradation by *P. gingivalis* proteases in the presence or absence of PPAD, as determined by Western blotting (SN, culture supernatant). (c and d) Quantification of significant changes in the amounts of phagocytosis-related proteins (c) and the antimicrobial histone H2B (d) in infected neutrophils, as approximated by mass spectrometry. (a, c, and d) Data are means of three replicates of one neutrophil donor. **, *P* < 0.01, two-tailed unpaired Student’s *t* tests. Data are presented as mean values ± SD. *, *P* < 0.05, Fisher’s exact test. Green and red arrows indicate up- or downregulation of >10% of the respective protein in W83-infected neutrophils. Norm., normalized.

How could PPAD mediate neutrophil evasion? An attractive hypothesis is that this involves the so-called gingipains of P. gingivalis, a group of highly proteolytic enzymes, including the arginine-specific enzymes RgpA and RgpB (13, 14). We recently reported that these gingipains are subject to citrullination by PPAD (6). Further, Maekawa et al. have previously shown that RgpA and RgpB induce Toll-like receptor 2 (TLR2)-C5aR cross talk, ultimately leading to the inhibition of actin polymerization and consequent inhibition of phagocytosis (44). We therefore assessed the RgpA and RgpB levels by Western blotting. As shown in Fig. 3a and S1d, the neutrophils are exposed to lower levels of RgpA and RgpB in the absence of PPAD. Moreover, the overall proteolytic activity in the growth medium of PPAD-deficient P. gingivalis is significantly reduced, as shown by a lowered rate of histone H3 protein degradation by PPAD-deficient W83 compared to that by the PPAD-proficient strain (Fig. 3b). Overall, in accordance with the model of Maekawa and colleagues (44), lower levels of RgpA and RgbB at the neutrophil surface, as observed for neutrophils infected with PPAD-deficient bacteria, will lead to less suppression of phagocytosis and therefore enhanced internalization of these bacteria, as shown in Fig. 2b. The underlying mechanism by which the presence of PPAD results in increased levels and activity of RgpA and RgpB is likely to be their previously documented citrullination by PPAD (6), which could confer protection against possible (self-)cleavage at arginine residues.

Furthermore, to verify the possibility that phagocytosis in neutrophils is decreased due to lower actin polymerization in the presence of PPAD-proficient bacteria, we applied a mass spectrometry-based approach. Indeed, the results show that the levels of the actin assembly-related proteins dynamin-2 (15), actin-related protein 2/3 (16), and the cell division control protein 42 (17) are decreased when neutrophils are challenged by wild-type P. gingivalis (Fig. 3c). This is consistent with a role of gingipain citrullination in the inhibition of actin polymerization and evasion of phagocytosis. However, our mass spectrometry analyses provide more clues as to how P. gingivalis corrupts the neutrophil. For example, the immunoglobulin κ constant protein is not detectable in neutrophils infected with wild-type P. gingivalis, while this protein is identified in neutrophils infected with the PPAD mutant (Fig. 3c). This implies a role of PPAD in inhibiting opsonization of the bacteria, as immunoglobulins are important in opsonization, which is the first step of phagocytosis. Altogether, a challenge with wild-type P. gingivalis leads to altered levels of 17 phagocytosis-related proteins compared to the PPAD mutant (Table S1). In particular, the levels of the integrins α-M and β-2, involved in actin polymerization (18), are reduced (Fig. 3c). These integrins play also crucial roles in cell signaling, neutrophil adhesion to endothelial cells, and granule exocytosis for releasing bactericidal toxins into the intracellular milieu (19). In fact, once a bacterial prey is internalized by neutrophils, several granule and cytosolic proteins facilitate its efficient destruction. Among these, the neutrophil cytosolic factor 4 (NCF4/p40phox) is involved in the oxidative burst that serves to kill internalized bacteria (20). Indeed, the NCF4 levels are also substantially lower when neutrophils are challenged with wild-type P. gingivalis than with PPAD-deficient bacteria (Fig. 3c). Last, the bactericidal histone H2B (21) is present in smaller amounts when neutrophils are exposed to PPAD-proficient P. gingivalis (Fig. 3d). Altogether, these findings show that P. gingivalis needs PPAD to escape internalization and subsequent elimination by neutrophils. Further, our results correlate the increased phagocytosis in the absence of PPAD to reduced levels of gingipains and a restricted impact of PPAD-deficient P. gingivalis on neutrophil proteins needed for phagocytosis.

10.1128/mBio.01704-18.6 TABLE S1 Overview of phagocytosis-related proteins identified in infected neutrophil samples. Twenty-five proteins with the gene ontology (GO) annotation “phagocytosis” were identified in neutrophils infected with P. gingivalis W83 or W83 ΔPPAD. Mean values of normalized spectral counts from three independent experiments are shown. Green and red arrows indicate up- or downregulation of >10% of the protein in the W83-infected neutrophils. Orange arrows indicate the absence of regulation. Stars indicate significance, based on *P* values lower than 0.05, as determined by Fisher’s exact test. Download Table S1, PDF file, 1.0 MB. Copyright © 2018 Stobernack et al. 2018 Stobernack et al. This content is distributed under the terms of the Creative Commons Attribution 4.0 International license.

### PPAD citrullinates histone H3 and helps evade NETs.

**FIG 4:**
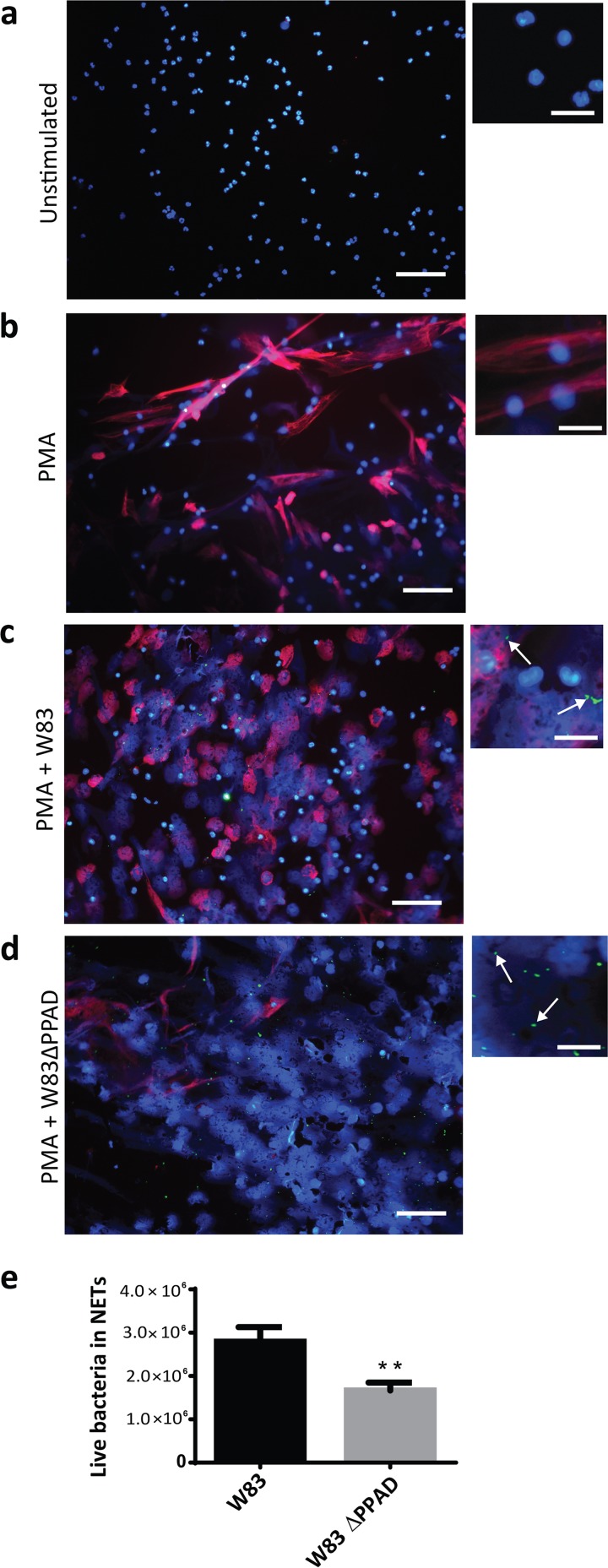
PPAD impacts on histone H3 citrullination and allows *P. gingivalis* to evade and survive capture in neutrophil extracellular traps (NETs). (a to d) Representative fluorescence microscopy images of NETosis and citrullinated histone H3 levels in the presence of *P. gingivalis*. PMA was applied at a concentration of 20 nM to induce NETosis. DNA was stained with DAPI (blue), *P. gingivalis* was labeled with FITC (green), and citrullinated histone H3 (citH3; red) was visualized with a specific antibody (scale bars, 200 µm in regular images and 50 µm in enlarged images). (e) Quantification of live bacteria present in isolated NETs.

Neutrophils can also capture bacteria with neutrophil extracellular traps (NETs), which are web-like structures mainly consisting of decondensed chromatin and bactericidal proteins (22, 23). Recent studies have shown that NETs are abundantly produced in periodontitis (24, 25). During the process of NET activation and release (known as NETosis), DNA-bound histones are citrullinated by the human peptidylarginine deiminases, leading to a change in charge and decondensation of the DNA (26). Of note, histones are known to have different roles in NET formation. On the one hand, the positive charge of histones is needed for their bactericidal effects. On the other hand, Li and colleagues have shown that citrullination of histone H3 by the human peptidylarginine deiminase 4 (PAD4) is essential for bacterial killing in NETs (27). The process of NETosis can be artificially induced by the addition of phorbol myristate acetate (PMA), as shown in Fig. 4a and b (see also Fig. S2). We exposed PPAD-proficient and PPAD-deficient P. gingivalis to neutrophils undergoing NETosis and observed higher NETosis in both infection situations than in the uninfected PMA-activated neutrophils. However, a greater number of intact neutrophil nuclei were noticed for PPAD-proficient bacteria than for the PPAD-deficient bacteria (Fig. 4c and d). This indicates that PPAD activity can impair the bacteria-induced NETosis. Consistent with this view, higher numbers of PPAD-deficient bacteria were observed to be trapped in NETs (Fig. 4c and d) and eliminated upon capture (Fig. 4e). The exact mechanisms by which PPAD could interfere with NET formation are currently unknown and should be a topic of future investigations. A possible explanation could be that the higher levels of secreted protease activity produced by the PPAD-proficient bacteria have a negative impact on the NET formation, for example, by degrading certain human proteins needed for DNA decondensation.

10.1128/mBio.01704-18.2 FIG S2 Fluorescence microscopy images of NETs, citrullinated histone H3, and P. gingivalis. (a) Unstimulated neutrophils. (b) Neutrophils undergoing NETosis induced by PMA. (c) Addition of P. gingivalis strain W83 to neutrophils undergoing NETosis. (d) Addition of P. gingivalis strain W83 ΔPPAD to neutrophils undergoing NETosis. DNA was stained with DAPI (blue), P. gingivalis was labeled with FITC (green), and citrullinated histone H3 (citH3; red) was visualized with a specific antibody. a to d, scale bars = 200 µm. Download FIG S2, PDF file, 3.2 MB. Copyright © 2018 Stobernack et al. 2018 Stobernack et al. This content is distributed under the terms of the Creative Commons Attribution 4.0 International license.

**FIG 5:**
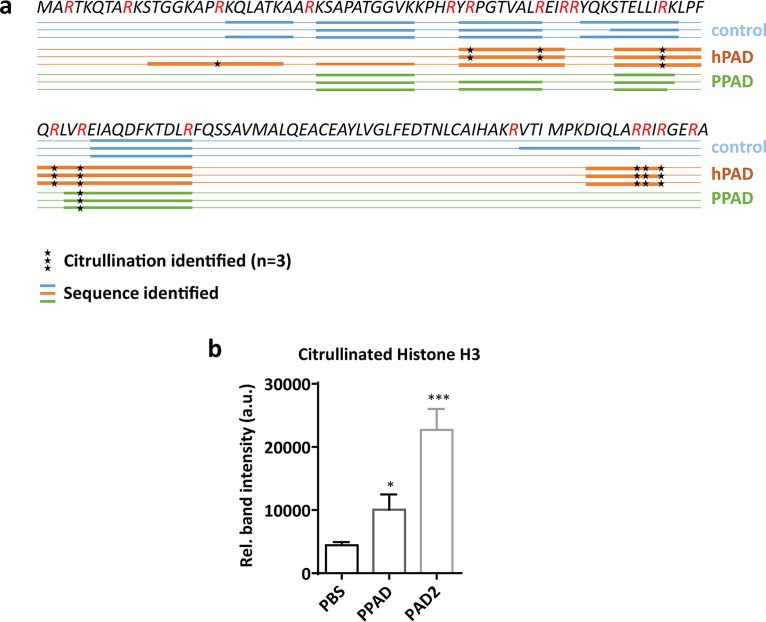
PPAD citrullinates histone H3. *In vitro* citrullination of histone H3. Citrullination by human PAD2 was used as a positive control. (a) Schematic representation of citrullinated arginine residues in histone H3 upon incubation with PPAD or PAD2, as determined by mass spectrometry. (b) Western blot analysis of citrullinated histone H3. Quantification of band intensity in three independent experiments is shown. *, *P < *0.05; ***, *P < *0.001, two-tailed unpaired Student’s *t* tests. Data are presented as mean values ± SD.

Histones are critical actors in capture and killing of bacteria in the NETs, and arginine-rich histones especially directly disrupt the bacterial cell membrane by virtue of their positive charge (21). We therefore inspected histone H3 citrullination in neutrophils undergoing NETosis, which revealed a strong PPAD-dependent citrullination of this antibacterial agent (Fig. 4c and d). This result was subsequently validated by incubating purified histone H3 with the recombinant PPAD, which led to histone H3 citrullination, as shown by Western blotting and mass spectrometry (Fig. 5a and b and S3a and b). Compared to the purified human peptidylarginine deiminase 2 (PAD2), PPAD showed a somewhat lower citrullinating activity on purified histone H3 that correlated with the citrullination of only one arginine residue (Arg73), whereas human PAD2 was capable of citrullinating up to nine different arginine residues in histone H3 (Fig. 5a). Even so, in terms of citrullination of the NET-associated histone H3, the impact of PPAD was much higher than that of any other human PAD released by neutrophils undergoing NETosis (Fig. 4b and d). These findings are fully consistent with the previously published observation that citrullinated histone H3 is abundantly detectable in inflamed periodontal tissue (28). Thus, P. gingivalis is capable of neutralizing a major NET-associated histone implicated in bacterial elimination in the periodontium, where PPAD is clearly detectable (Fig. 1b).

### PPAD citrullinates human lysozyme-derived peptide LP9, neutralizing its antibacterial activity.

**FIG 6:**
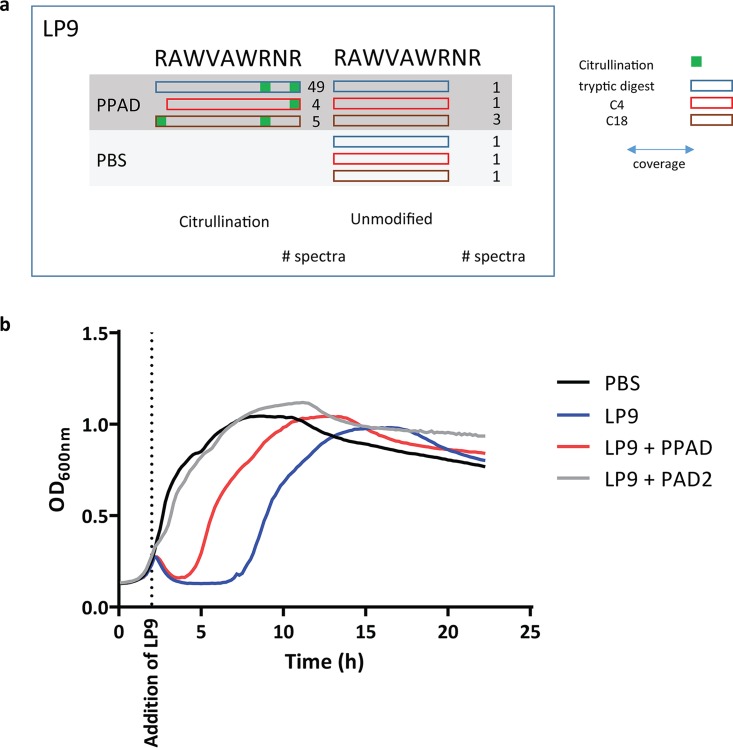
PPAD citrullinates human lysozyme-derived peptide LP9, neutralizing its antibacterial activity. (a) Arginine residues in the LP9 peptide (RAWVAWRNR) are citrullinated by PPAD, as determined by mass spectrometry. Blue, red, and brown rectangles mark the outcomes from three distinct analytical approaches, tryptic digest, C_4_ exclusion filtration, and C_18_ inclusion filtration, respectively. (b) Citrullination of LP9 by PPAD or PAD2 impairs the antibacterial activity of LP9. Citrullinated LP9 exhibits significantly reduced growth inhibition of the indicator bacterium *B. subtilis*. Results are representative of three independent experiments, with three technical replicates per experiment. OD_600_, optical density at 600 nm.

The bacterial cell wall-degrading enzyme lysozyme is an important contributor to human innate immunity. This enzyme, abundantly present in our saliva, is also produced by neutrophils (29, 30). It acts in two different modes, the first that the full-size protein has muramidase activity that degrades peptidoglycan, leading to bacterial lysis. In addition, degradation products of lysozyme act as cationic antimicrobial peptides (CAMPs), as was shown for the LP9 peptide (_107_RAWVAWRNR_115_) (31). LP9 introduces pores into the bacterial cell membrane by electrostatic interaction, leading to bacterial death. Presumably, this relates to LP9’s three arginine residues. We therefore tested whether PPAD can neutralize LP9 by citrullination, thereby abrogating its bactericidal activity toward LP9-susceptible bacteria. This is indeed the case, as mass spectrometry showed that PPAD can convert all three arginines of LP9 to citrulline (Fig. 6a). Concomitantly, citrullination reduced the bactericidal activity of LP9, as demonstrated with the LP9-susceptible indicator Bacillus subtilis (Fig. 6b). This shows that PPAD can even neutralize CAMPs, which belong to our most effective defenses against bacterial pathogens. Notably, PPAD-proficient and PPAD-deficient P. gingivalis strains are not susceptible to LP9 (Fig. S4). This shows that PPAD is not the only factor that protects P. gingivalis against LP9 activity. In fact, this finding is in agreement with the previous observation that gingipains play an important role in the deactivation of CAMPs by proteolytic degradation (32, 33).

### PPAD is a critical virulence factor of P. gingivalis.

**FIG 7:**
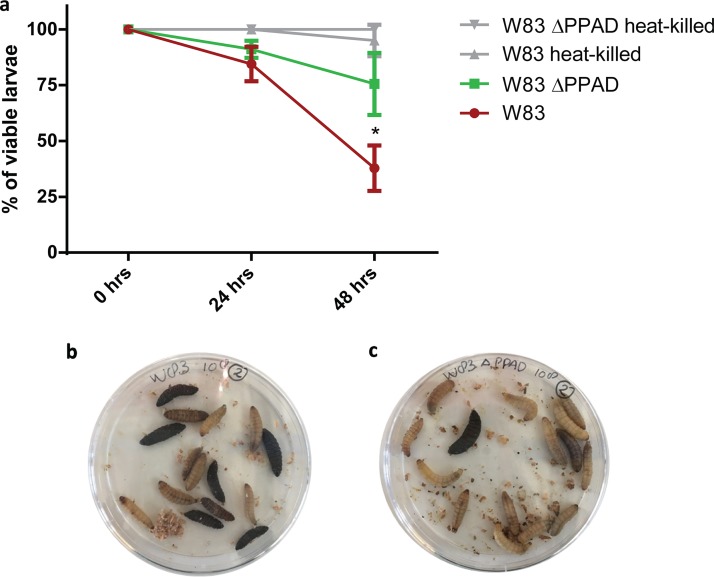
PPAD is a critical virulence factor of *P. gingivalis*. Viability of *Galleria mellonella* larvae was measured 24 h and 48 h after infection with *P. gingivalis.* (a) The larvae were significantly less susceptible to *P. gingivalis* W83 ΔPPAD than to the wild-type strain W83. Heat-killed bacteria were used as a negative control. Data are means of three biological replicates (*n* = 15). (b and c) Representative images of *G. mellonella* larvae infected with *P. gingivalis* W83 (b) or W83 ΔPPAD (c) *, *P <* 0.05, two-tailed unpaired Student’s *t* tests.

While the above-mentioned studies show that PPAD targets innate immunity at three different levels, an important question that remained to be addressed was whether it contributes *in vivo* to the virulence of P. gingivalis. This was investigated using larvae of the wax moth Galleria mellonella, because this infection model only possesses an innate immune system. Hemocytes, the main innate immune cells of G. mellonella, closely resemble human neutrophils, since they employ the same defense mechanisms, in particular, phagocytosis and NETosis (34). As shown in Fig. 7, G. mellonella larvae are less susceptible to injected PPAD-deficient P. gingivalis than to the wild-type bacteria, whereas heat-killed P. gingivalis bacteria do not affect larval viability. This observation is fully in line with the here-proposed role of PPAD as an immune evasion factor.

### Conclusion.

Altogether, our present findings show for the first time that the virulence factor PPAD of the oral pathogen P. gingivalis defuses antibacterial neutrophil insults at three distinct levels, namely, phagocytosis, NETosis, and CAMP activity. This identifies PPAD as a major agent in the evasion of human innate immunity, a view that is supported by studies from Potempa and coworkers showing PPAD-dependent citrullination of the complement system (35). Importantly, an essential role of PPAD in immune evasion explains why this enzyme is invariantly produced by all of the over 100 clinical isolates of P. gingivalis investigated to date (8, 36).

## MATERIALS AND METHODS

### Immunohistochemistry.

Immunohistochemical staining of PPAD was performed as described before (28). Briefly, human paraffin-embedded gingival tissues were collected from P. gingivalis-colonized periodontitis patients at the dentistry department of the University Medical Center Groningen. Deparaffinization of 5-µm sections was performed by several xylene, ethanol, and water washes. Endogenous peroxidase activity was inhibited by the addition of hydrogen peroxide in methanol, followed by blocking of nonspecific antibody binding with 1% bovine serum albumin and 1% normal goat serum in phosphate-buffered saline (PBS). Next, samples were stained either with an in-house-produced PPAD-specific antibody (7, 8) or with the respective preimmune serum (1:100 in PBS, 1 h). Upon removal of excessive primary antibody by PBS, a secondary goat anti-rabbit IgG horseradish peroxidase (HRP) antibody (P0448; Dako, Santa Clara, CA, USA) was added at a concentration of 1:100 in PBS for 45 min, followed by washing and a developing reaction using a 3,3′-diaminobenzidine (DAB) kit (K3467; Dako). Sections were counterstained with hematoxylin and mounted with glycerine before microscopic evaluation.

### P. gingivalis culture.

The P. gingivalis reference strain W83 and the respective PPAD-deficient mutant (W83 ΔPPAD) (37) were grown as described before (6). For infection experiments, liquid cultures were grown until stationary phase, which was reached after ∼24 h. For several experiments, inoculation was performed by diluting bacterial glycerol stocks stored at −80°C in a 1:100 ratio into fresh brain heart infusion (BHI) medium (Oxoid, Basingstoke, UK).

### Neutrophil isolation.

Neutrophils were freshly isolated from four healthy donors (two females age 27 and 34 years and two males age 28 and 39 years) who had been medically examined. Lymphoprep buffer (StemCell Technologies, Vancouver, Canada) was used to separate cell types. EDTA-blood was first diluted 1:1 with PBS and then put gently on top of a volume of Lymphoprep (blood-to-Lymphoprep ratio, 2:1). Samples were centrifuged at 2,500 rpm at room temperature (RT) for 20 min without brake so as not to disrupt the separated cell layers. The plasma, Lymphoprep, and peripheral blood mononuclear cells were removed, and a layer of erythrocytes and neutrophils remained. The erythrocytes in this mixture were lysed by adding ammonium chloride 0.8% and 1 mM EDTA (pH 7.4) and shaking for 10 min on ice. After another centrifugation at 2,500 rpm for 3 min, the lysed erythrocytes were removed. These two steps were repeated once more to obtain a pellet of purified neutrophils.

### OMV and PPAD preparation.

P. gingivalis cultures in late-exponential phase were used for OMV collection. A first centrifugation step at 8,000 × *g* and 4°C for 20 min was performed to separate cells from OMV-containing supernatant. The supernatant was subjected to ultracentrifugation at 100,000 × *g* and 4°C for 3 h in an Optima MAX-XP ultracentrifuge 261 (Beckman Coulter, Brea, CA, USA) using an MLA-80 fixed-angle rotor. The pellet containing the OMVs was resuspended in PBS, and aliquots were frozen at −80°C before use. Protein quantification was performed using a bicinchoninic acid (BCA) protein assay (Pierce, Waltham, MA, USA), according to the manufacturer’s instructions, with the addition of 2.0% SDS to solubilize proteins. Sixteen micrograms of protein was used for the phagocytosis rescue experiment. Protein precipitation with 10% tricarboxylic acid (TCA) was performed as described before (6) to concentrate vesicles for Western blot analysis. Recombinant PPAD was purified from Lactococcus lactis, as previously described (7, 8).

### Neutrophil infections.

For neutrophil infection experiments followed by Western blotting or mass spectrometry analyses, 3 × 10^6^ neutrophils in 2.5 ml of RPMI 1640 medium (Gibco, Waltham, MA, USA) with 2 mM l-glutamine and 10% autologous donor serum were seeded in each well of a 6-well plate. Phagocytosis experiments were carried out with 5 × 10^5^ neutrophils in 500 µl of medium in 24-well plates. The neutrophils were allowed to rest on the plate at 37°C and 5% CO_2_ for 1 h. When required, 100 µl supernatant of W83, 100 µl BHI medium, 2.5 µg recombinant PPAD, 16 µg OMVs of W83, or 100 µl PBS were added to the neutrophil suspension, and incubation was continued for 30 min. Subsequently, P. gingivalis was added at a multiplicity of infection (MOI) of 100. The neutrophils were exposed to the bacteria for 90 min. Extracellular bacteria were then removed, and the neutrophil layer was washed once with PBS before the addition of NP-40 lysis buffer (150 mM sodium chloride, 1.0% NP-40, 50 mM Tris [pH 8.0]) with cOmplete mini protease inhibitor (Roche, Basel, Switzerland).

### Phagocytosis assay.

To determine whether PPAD impacts the association and/or internalization of P. gingivalis in neutrophils as defined by Lei et al. (38), a flow cytometry-based method was used as described previously (39). Briefly, a liquid bacterial culture was centrifuged for 10 min at 7,000 × *g* and 4°C and washed once in PBS before resuspending the bacterial pellet in 0.5 M NaHCO_3_ (pH 8.0) to a concentration of 2.5 × 10^9^ CFU/ml before the addition of fluorescein isothiocyanate (FITC; Invitrogen, Carlsbad, USA). Bacterial concentrations were approximated by optical density readings at 600 nm according to a standard curve for each strain used.

An FITC concentration of 0.15 mg/ml was used for staining P. gingivalis W83 and W83 ΔPPAD (39, 40). The tubes with bacteria and FITC were subsequently incubated in the dark for 30 min at RT in a tube rotator. The bacteria were pelleted at 7,000 × *g* for 5 min, and the pellet was washed 3 times with PBS to remove unbound FITC. Finally, the bacteria were resuspended to the desired concentration in RPMI 1640–10% autologous donor serum–2 mM l-glutamine.

To measure the bacterial internalization rate, the extracellular fluorescence (representing associated but not internalized bacteria) was quenched using 0.2% trypan blue (Thermo Fisher Scientific, Waltham, MA, USA). Subsequently, two washing steps with PBS were performed to remove excessive trypan blue. Both quenched and nonquenched cell samples were fixed with 4% paraformaldehyde (PFA; Sigma-Aldrich, St. Louis, MO, USA) for 15 min prior to flow cytometric analyses and visualization by fluorescence microscopy.

An Accuri C6 flow cytometer was used to measure the mean fluorescence intensity (MFI) of the FITC-positive cells. The gating strategy to include only neutrophils in our analysis is shown in Fig. S1e to g. FITC-positive cells were identified by setting a fluorescence threshold in an uninfected neutrophil control sample, next to the autofluorescence peak, as shown in Fig. S1h to j. The association index of each P. gingivalis strain was calculated by multiplying the percentage of FITC-positive cells with associated bacteria (i.e. intracellular plus extracellularly bound bacteria) with the MFI of these cells, divided by 100, as previously described (41). The internalization index of each P. gingivalis strain was calculated by multiplying the percentage of cells with internalized bacteria (cells positive for FITC after trypan blue quenching) with the MFI of these cells, divided by 100 (38). For microscopic analyses, 10 µl of the fixed cells was mounted on microscopy slides and visualized with an Axio Observer.Z1 fluorescence microscope (Zeiss, Jena, Germany) using ×40 or ×65 magnification. Images were recorded using an Axio Cam MRm Rev. 3 camera with FireWire.

### LDS-PAGE.

Lithium dodecyl sulfate (LDS)-PAGE was performed using 10% NuPAGE gels (Invitrogen, Carlsbad, CA, USA). Protein concentrations of cell lysates were determined with the Pierce BCA protein assay kit (Thermo Fisher Scientific, Waltham, MA, USA) and frozen at −20°C until further use. Equal amounts of protein samples were incubated with LDS sample buffer for 10 min at 95°C, separated by LDS-PAGE, and either stained with SimplyBlue SafeStain (Life Technologies, Carlsbad, CA, USA) or processed further for Western blotting.

### Western blotting.

For Western blotting, proteins were transferred from the gel to a nitrocellulose membrane (Whatman, Buckinghamshire, UK) by semidry blotting. The transfer was performed at 200 mA for 75 min in the presence of methanol-containing buffers. Upon transfer, the nonspecific binding was blocked overnight at 4°C with 5% skim milk (Oxoid, Basingstoke, UK) in PBS. Afterwards, the blot was rinsed once with PBS-Tween 20 (PBS-T) to remove residual skim milk. Primary rabbit anti-RgpA/B, rabbit anti-PPAD antibodies (7, 8) or anti-histone H3 (ab18521; Abcam) in PBS-T (1:2,000) were added, and the blot was incubated for 1 h at RT. After removing the nonbound primary antibodies by 4 washes with PBS-T, the blot was incubated with IRDye 800CW goat anti-rabbit antibody (LI-COR Biosciences, Lincoln, NE, USA) in PBS-T (1:10,000) protected from light for 45 min. Last, background was reduced by washing 4 times with PBS-T and subsequently washing twice with PBS to remove the Tween. Fluorescence was measured with the LI-COR Odyssey infrared imaging system (LI-COR Biosciences, Lincoln, USA) and subsequently quantified using ImageJ (National Institutes of Health, Bethesda, MD, USA).

### Protease activity assay.

P. gingivalis was grown in BHI medium until stationary phase, and the growth medium was separated from the cells by centriguation at 7,000 × *g* for 10 min. Recombinant human histone H3 (0.5 µg; New England BioLabs, Ipswich, MA, USA) was incubated with 7.5 µl of the growth medium fraction for 1, 5, 10, 15, 20, and 30 min at 37°C. Fresh BHI medium (7.5 µl) was used as a negative control. The resulting protein samples were analyzed by Western blotting, as described above.

### Mass spectrometry of neutrophils.

Neutrophil lysates were processed for mass spectrometry analysis, as described before (42). Briefly, proteins were bound to StrataClean resins (Agilent Technologies, Santa Clara, CA, USA) and subsequently alkylated, reduced, and digested by trypsin. The resulting peptides were purified by C_18_ stage-tip purification (Thermo Fisher Scientific, Waltham, MA, USA), according to the manufacturer’s protocol, and dried until further use.

Purified peptides were analyzed by reversed-phase liquid chromatography (LC) electrospray ionization-tandem mass spectrometry (ESI-MS/MS) using an Orbitrap Elite mass spectrometer (Thermo Fisher Scientific, Waltham, MA, USA). In brief, in-house self-packed nano-LC columns (20 cm) were used to perform LC with an EASY-nLC 1200 system (Thermo Fisher Scientific). The peptides were loaded with buffer A (0.1% [vol/vol] acetic acid) and subsequently eluted in 156 min using a 1% to 99% nonlinear gradient with buffer B (0.1% [vol/vol] acetic acid, 94.9% acetonitrile). After injection into the MS, a full scan was recorded in the Orbitrap MS with a resolution of 60,000. The 20 most abundant precursor ions were consecutively isolated in the linear ion trap and fragmented via collision-induced dissociation (CID). Unassigned charge states as well as singly charged ions were rejected, and the lock mass option was enabled.

Database searching was done with Sorcerer-Sequest 4 (Sage-N Research, Milpitas, CA, USA). After extraction from the raw files, *.dta files were searched with Sequest against a target-decoy database with a set of common laboratory contaminants. A database for the respective peptide/protein search was created from the published genome sequences of the W83 strain and the human genome, which were downloaded from UniProt (http://www.uniprot.org) on 14 July 2016. The created database contained a total of 148,472 proteins. Database search was based on a strict trypsin digestion with two missed cleavages permitted. No fixed modifications were considered. Oxidation of methionine, carbamidomethylation of cysteine, and citrullination of arginine were considered variable modifications. The mass tolerance for precursor ions was set to 10 ppm and the mass tolerance for fragment ions to 1 Da. Validation of MS/MS-based peptide and protein identification was performed with Scaffold version 4 (Proteome Software, Portland, OR, USA). A false-discovery rate (FDR) of 0.1% was set for filtering the data. Protein identifications were accepted if at least 2 identified peptides were detected with the above-mentioned filter criteria in 2 out of 3 biological replicates. Protein data were exported from Scaffold and further curated in Microsoft Excel 2013 before further analysis.

Quantitative values of protein abundances in neutrophil samples were obtained by summing up all spectra associated with a specific protein within a sample, which includes also those spectra that are shared with other proteins. To allow comparisons, spectral counts were normalized by applying a scaling factor for each sample to each protein adjusting the values to normalized quantitative values.

### Mass spectrometry of histone H3 and LP9.

Recombinant human histone H3 (0.5 µg; New England BioLabs, Ipswich, MA, USA) was incubated with recombinant PPAD (0.25 µg) overnight at 37°C. Proteins were separated by LDS-PAGE and stained with SimplyBlue SafeStain, as described above. Histone H3-corresponding bands (Fig. S3b) were excised from the gel, dried, and further processed by trypsin digestion as described above.

10.1128/mBio.01704-18.3 FIG S3 Histone H3 citrullination by PPAD. (a) Recombinant human histone H3 becomes citrullinated by PPAD in a time-dependent manner, as determined by Western blotting. Human PAD2 was used as a positive control for citrullination. (b) Recombinant human histone H3 was incubated with purified recombinant PPAD or human PAD2 and separated by LDS-PAGE for subsequent citrullination assessment by mass spectrometry. Protein bands were stained with SimplyBlue SafeStain. Download FIG S3, PDF file, 2.1 MB. Copyright © 2018 Stobernack et al. 2018 Stobernack et al. This content is distributed under the terms of the Creative Commons Attribution 4.0 International license.

10.1128/mBio.01704-18.4 FIG S4 Resistance of P. gingivalis to LP9. LP9 does not inhibit the growth of PPAD-deficient P. gingivalis. The results are representative of two independent experiments, with three technical replicates per experiment. Download FIG S4, PDF file, 0.8 MB. Copyright © 2018 Stobernack et al. 2018 Stobernack et al. This content is distributed under the terms of the Creative Commons Attribution 4.0 International license.

LP9 was synthesized at EMC microcollections GmbH (Tübingen, Germany). The LP9 peptide (0.5 µg) was incubated with recombinant PPAD (0.25 µg) overnight at 37°C. Subsequently, the samples were processed by three different methods, as follows: (i) trypsin digestion, in which samples were alkylated, reduced, digested by trypsin, and purified by C_18_ ZipTip purification, as described above; (ii) C_4_ Exclusion of PPAD by C_4_ ZipTip filtration using a slight modification of the manufacturer’s protocol, where upon binding of PPAD to the ZipTip, the PPAD-containing tip was discarded and the LP9-containing flowthrough was further processed by C_18_ ZipTip filtration; and (iii) C_18_ inclusion of LP9, where the LP9 peptides were immediately purified by C_18_ ZipTip filtration following the manufacturer’s protocol.

Purified peptides were analyzed by reversed-phase LC-ESI-MS/MS using an Orbitrap Elite spectrometer (Thermo Fisher Scientific, Waltham, MA, USA). In brief, in-house self-packed nano-LC columns (20 cm; packed with Aeris peptide material, 3.6-µm XB-C_18_-100Å) were used to perform LC with an Easy-nLC 1200 system (Thermo Fisher Scientific). The peptides were loaded with buffer A (0.1% [vol/vol] acetic acid) and subsequently eluted in 80 min using a nonlinear gradient of 1% to 99% with buffer B (0.1% [vol/vol] acetic acid, 94.9% acetonitrile). After injection into the MS, a full scan was recorded in the Orbitrap spectrometer with a resolution of 60,000. The 20 most abundant precursor ions were consecutively isolated in the linear ion trap and fragmented via CID. Unassigned charge states and singly charged ions were rejected, and the lock mass option was enabled.

Database searching for the histone H3 and LP9 analyses was done with Sorcerer-Sequest 4 (Sage-N Research, Milpitas, CA, USA). After extraction from the raw files, *.dta files were searched with Sequest against a target-decoy database with a set of common laboratory contaminants. For the peptide/protein search, the sequence of LP9 was added to the database that was used for analysis of the neutrophil MS data, and the database search was performed based on the same criteria as described above. For the histone H3 analysis, Sequest identifications required XCorr scores of greater than 2.2, 3.3, and 3.8 for doubly, triply, and all higher-charged peptides, respectively. For the LP9 analysis, Sequest identifications required XCorr scores of greater than 2.7, 3.5, and 3.5 for doubly, triply, and all higher-charged peptides, respectively. Protein data were exported from Scaffold. Spectra and fragmentation tables of the peptides identified to be citrullinated are presented in Fig. S5.

10.1128/mBio.01704-18.5 FIG S5 Spectra and fragmentation tables of the peptides of the human histone H3 and the CAMP LP9, which were identified as being citrullinated. These data were obtained from Proteome Software Scaffold version 4. Download FIG S5, PDF file, 45.6 MB. Copyright © 2018 Stobernack et al. 2018 Stobernack et al. This content is distributed under the terms of the Creative Commons Attribution 4.0 International license.

### Immunofluorescence microscopy of NET formation.

For microscopic analysis of infected neutrophils, sterile 12-mm-diameter coverslips (Menzel-Gläser, Braunschweig, Germany) were placed into 24-well plates (Corning, Corning, NY, USA). A total of 2.5 × 10^5^ neutrophils in 500 µl RPMI 1640 medium were added to each well. To let the neutrophils adhere to the coverslips, plates were incubated for 1 h at 37°C and 5% CO_2_. Subsequently, cells were stimulated for 1 h with 20 mM phorbol myristate acetate (PMA; Sigma-Aldrich, St. Louis, MO, USA) to induce NETosis and then infected with P. gingivalis at an MOI of 100 for 90 min. Upon infection, 500 µl of 8% PFA was added to each well to reach a final concentration of 4% PFA to fix the cells. Plates were stored at 4°C in the dark, and immunofluorescence staining was performed on the following day. For this, the fixative solution was removed, and the cell layer was washed carefully one time with PBS. A blocking step was performed by incubating cells at room temperature (RT) with 2% bovine serum albumin (BSA) in PBS for 1 h. Citrullinated histone H3 in NETs was stained with a rabbit anti-citrullinated histone H3 antibody (ab5103, 1:250; Abcam) and incubated for 1 h at RT in PBS, 0.05% Tween 20, and 0.5% BSA. Coverslips were washed with PBS before adding secondary antibodies. The Alexa Fluor 568 goat anti-rabbit antibody (catalog no. A11011, 1:400; Invitrogen) was used to visualize the primary antibodies. Secondary antibodies were added in PBS with 4′,6-diamidino-phenylindole (DAPI; product no. 10236276001, 1:5,000; Roche) and incubated for 30 min before mounting the coverslips in citifluor (CitiFluor, Hatfield, PA, USA). Slides were then analyzed using a Leica DFC450 C fluorescence microscope with the Leica Application Suite software version 4.2.0.

### NET survival assay.

NETosis was induced, and P. gingivalis was added to the NETs as described above, with the modification that no coverslips were placed into the wells. Upon 90 min of infection, NETs were isolated as described previously (43). Subsequently, different dilutions of bacteria trapped in the NETs were plated on blood agar base no. 2 (BA2) plates (Oxoid, Basingstoke, UK). The plates were incubated for 5 days at 37°C under anaerobic conditions, and P. gingivalis colonies were counted.

### Citrullination of LP9 and killing assay.

Bacillus subtilis strain 168 was grown overnight in BHI broth (Oxoid, Basingstoke, UK) with shaking at 37°C. The culture was diluted to an optical density at 600 nm of 0.1, and 100 µl of this suspension was pipetted in each well of a 96-well plate. Bacteria were grown for 2 h shaking at 37°C in a Biotek Synergy 2 microplate reader (Biotek Instruments, Inc., Winooski, VT, USA) until they reached exponential phase, and LP9 (in PBS) was added at a final concentration of 200 µg/ml. To investigate the effect of citrullination on its activity, LP9 was preincubated with PPAD or human peptidylarginine deiminase 2 (hPAD2) overnight at 37°C before its addition. Bacterial growth was monitored until stationary phase, and the respective growth curves were plotted with GraphPad Prism version 6 (GraphPad Software, La Jolla, CA, USA). The effect of LP9 on exponentially growing cells was determined by measuring the growth delay of B. subtilis upon the addition of LP9. The same procedure was applied for the killing assay of P. gingivalis. However, for P. gingivalis, standing cultures were grown for 48 h at 37°C.

### *In vivo* Galleria mellonella survival assay.

Larvae of G. mellonella were injected with the P. gingivalis W83 strain or the respective PPAD-deficient mutant. Bacteria were injected into the last proleg at a volume of 10 µl using a HumaPen Luxura HD pen (Eli Lilly, Indianapolis, IN, USA). Viability was scored by one trained person at 24 h and 48 h postinfection based on pigmentation and mobility. To assess the virulence of the investigated P. gingivalis strains, the larvae were infected with 10^8^ PBS-washed bacteria. Heat-killed bacteria (30 min, 90°C) were used as a negative control.

### Statistical analyses.

Statistical analyses were performed with GraphPad Prism version 6 (GraphPad Software, La Jolla, CA, USA) or with Scaffold version 4 (Proteome Software, Portland, OR, USA). Two groups were compared by performing an unpaired two-tailed Student’s *t* test. Fisher’s exact test was used to assess the significance of differences in normalized spectral counts of neutrophil proteins detected by MS. Significance was defined as a *P* value lower than or equal to 0.05.

### Medical ethics committee approval.

Blood donations from healthy volunteers were collected with approval of the medical ethics committee of the University Medical Center Groningen (UMCG; approval no. Metc2012-375). All blood donations were obtained after written informed consent from all volunteers and adhering to the Declaration of Helsinki guidelines.

### Biological and chemical safety.

P. gingivalis was handled following appropriate safety and containment procedures for biosafety level 2 microbiological agents. All experiments involving human cells were performed under appropriate safety conditions. All chemicals and reagents applied in this study were handled according to local guidelines for safe usage and protection of the environment.

### Data availability.

The mass spectrometry data are deposited in the ProteomeXchange repository PRIDE: https://www.ebi.ac.uk/pride/archive/projects/PXD010798 (neutrophil infection) and https://www.ebi.ac.uk/pride/archive/projects/PXD009081 (histone H3 and LP9).
