# Supplementary figures and images for: A Secreted Bacterial Peptidylarginine Deiminase Can Neutralize Human Innate Immune Defenses
Source: mBio. 2018 Oct 30;9(5):e01704-18. doi: 10.1128/mBio.01704-18 (PMC6212822; doi:10.1128/mBio.01704-18)

# Supplementary Figure S1

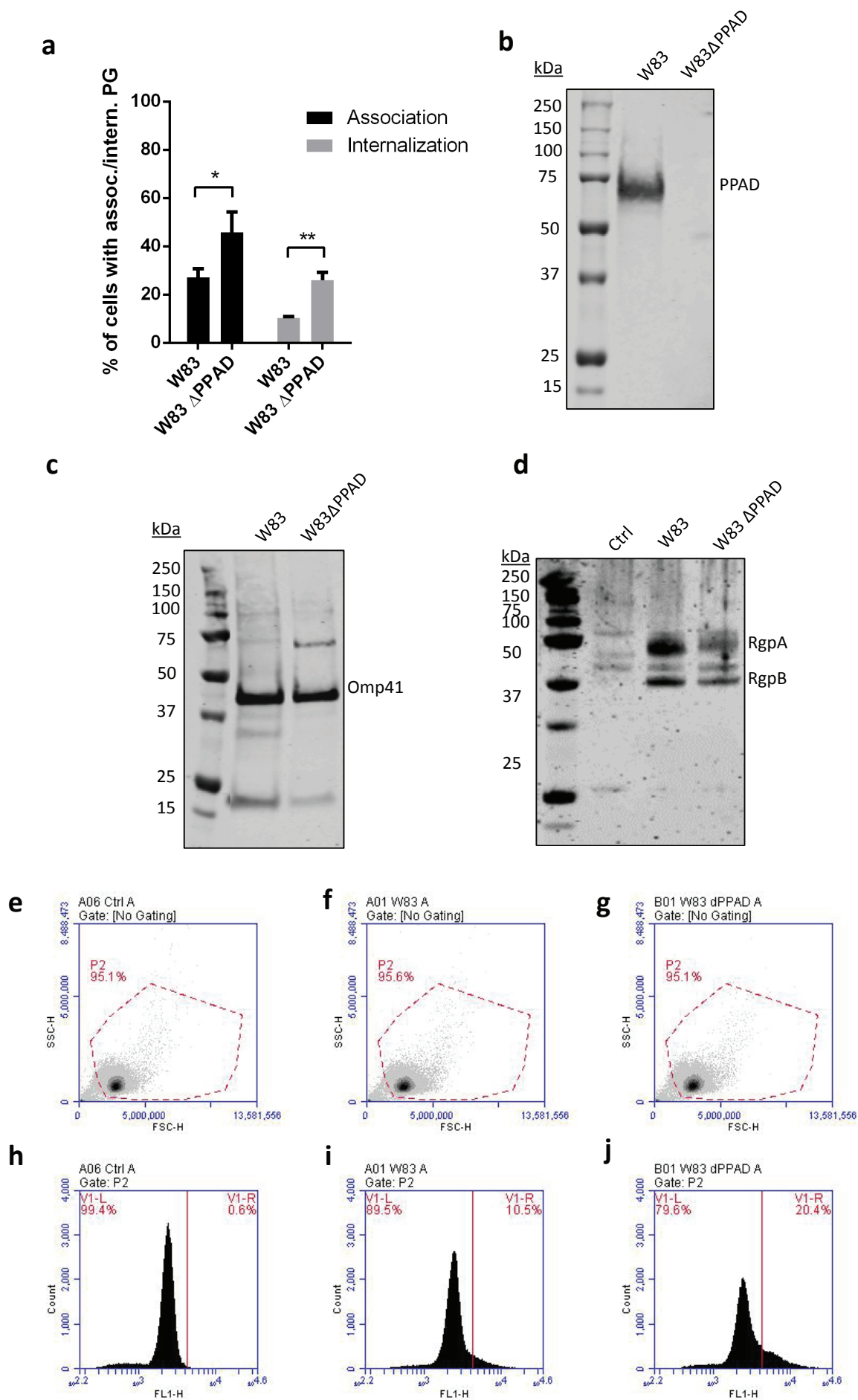

Supplement: FIG S1 [file mbo005184137sf1.pdf]

Supplementary Figure S2

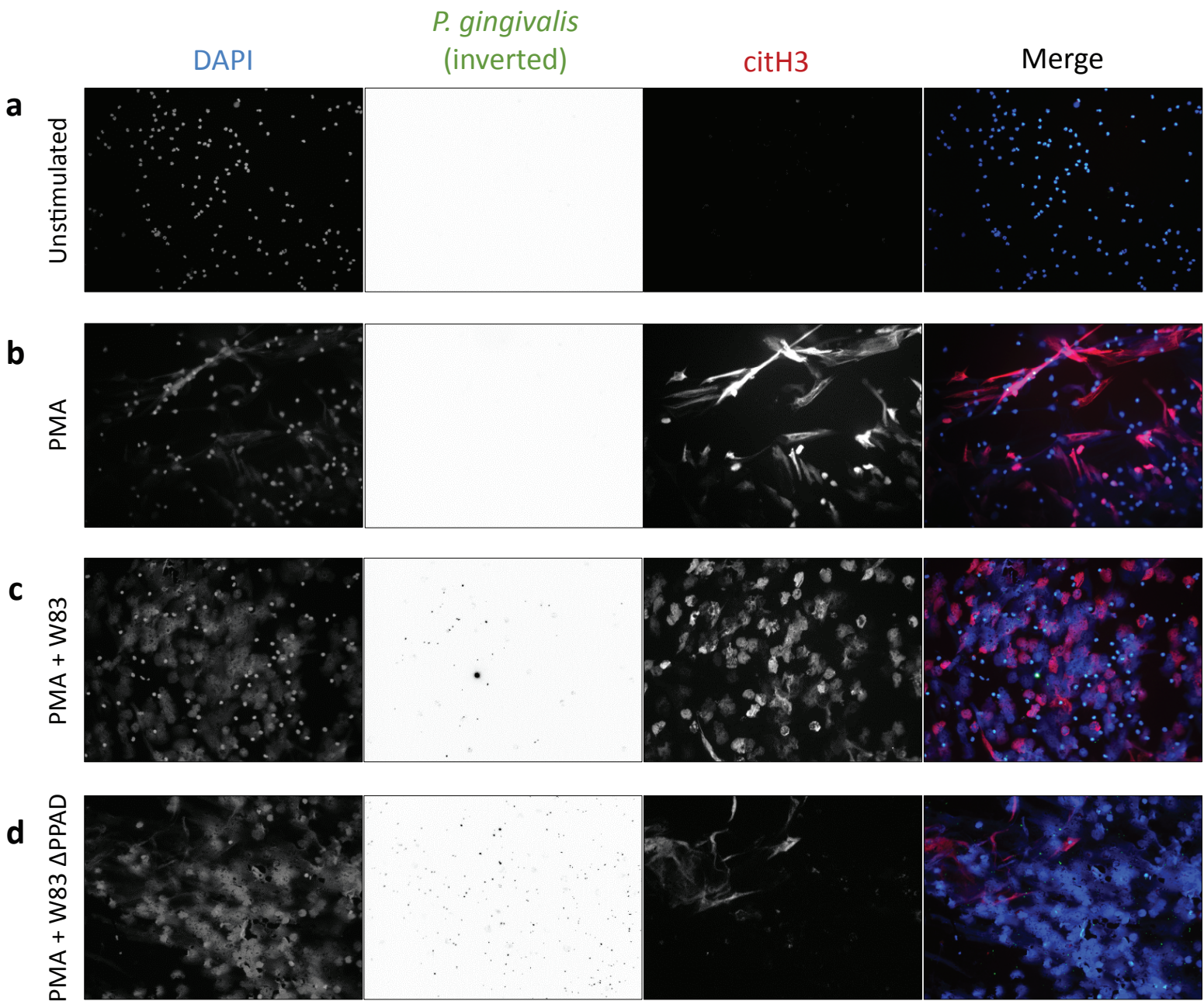

Supplement: FIG S2 [file mbo005184137sf2.pdf]

## Supplementary Figure S3

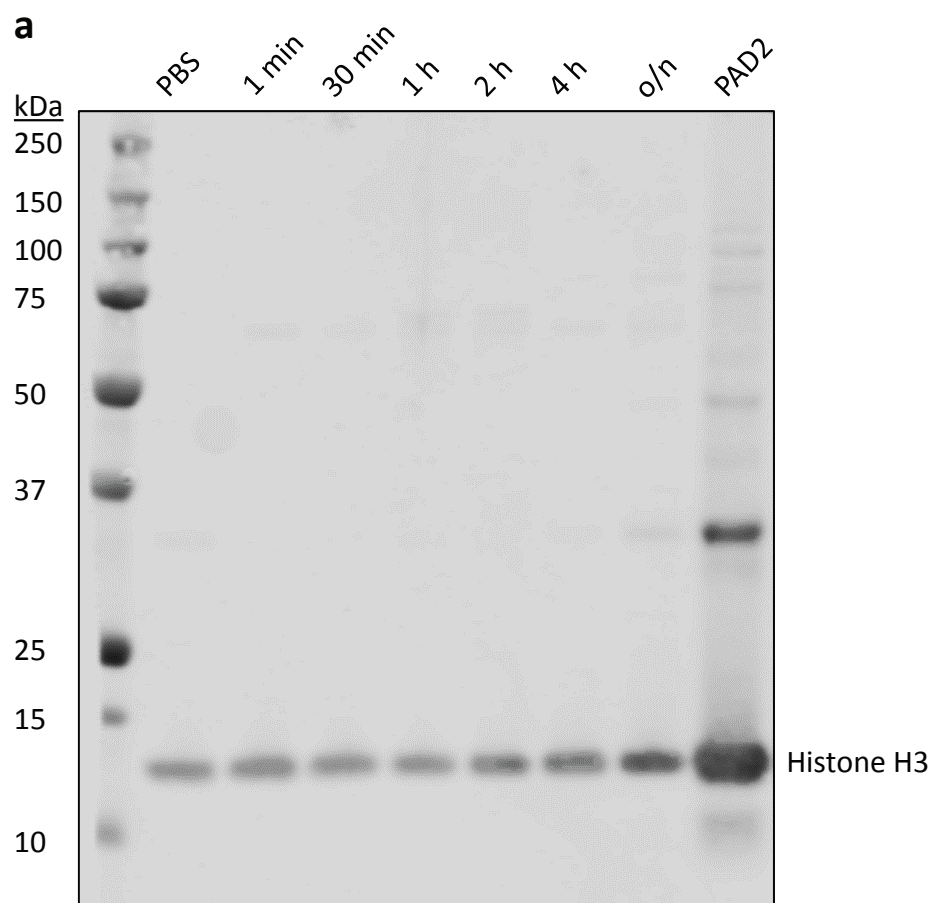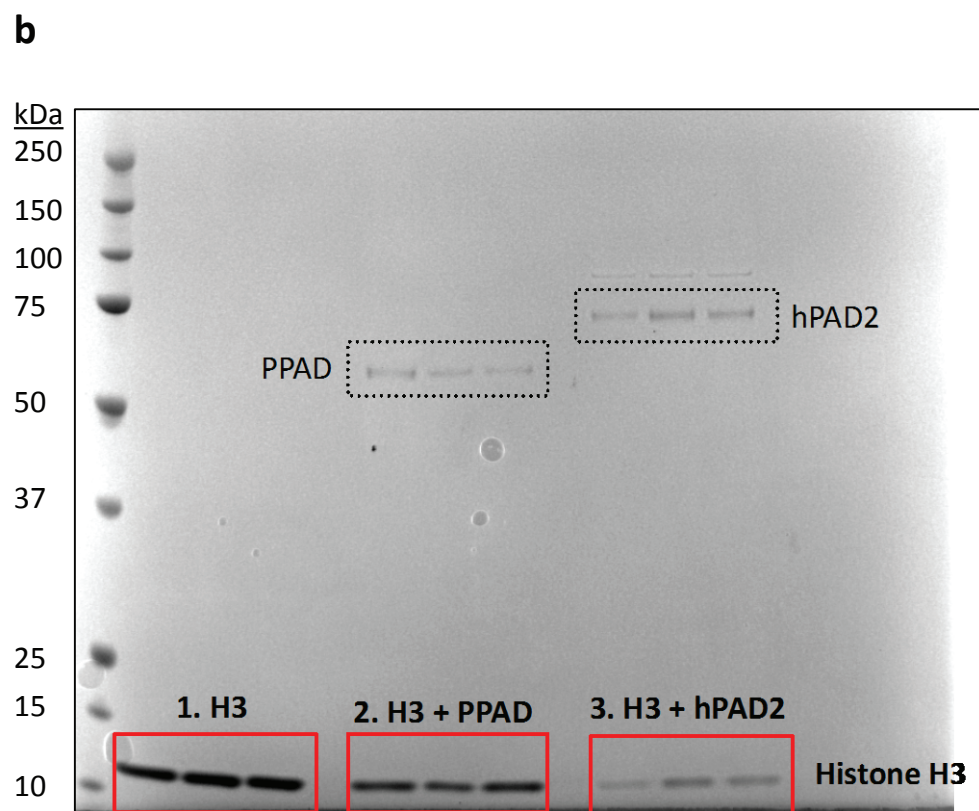

Supplement: FIG S3 [file mbo005184137sf3.pdf]

Supplementary Figure S4

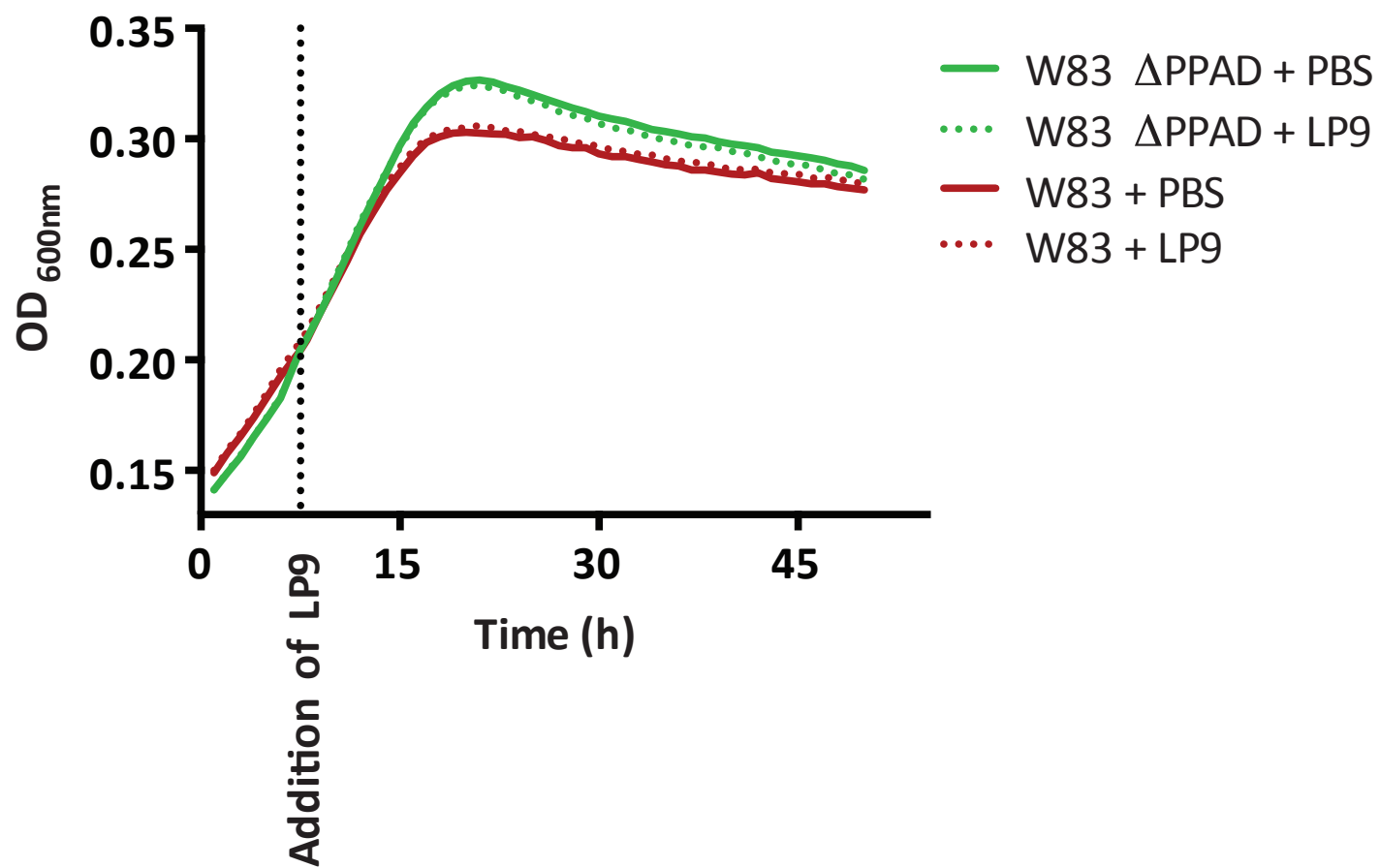

Supplement: FIG S4 [file mbo005184137sf4.pdf]
