# Supplementary material for: A Secreted Bacterial Peptidylarginine Deiminase Can Neutralize Human Innate Immune Defenses
Source: mBio. 2018 Oct 30;9(5):e01704-18. doi: 10.1128/mBio.01704-18 (PMC6212822; doi:10.1128/mBio.01704-18)
Supplement: TABLE S1 [file mbo005184137st1.pdf]

# Supplementary Table S1

| | | | Normalized Spectral Counts | |
|---|---|---|---|---|
| **Protein Name** | **Uniprot ID** | **Impact of PPAD** | **Neutrophils + W83** | **Neutrophils + W83 ΔPPAD** |
| Actin, cytoplasmic 2 | ACTG_HUMAN | ↓ | 23.23 | 26.12 |
| Actin-related protein 2/3 complex subunit 1B | ARC1B_HUMAN | → | 5.40 | 5.87 |
| Actin-related protein 2/3 complex subunit 2 | ARPC2_HUMAN | ↓ | 6.26 | 7.06 |
| Actin-related protein 2/3 complex subunit 3 | ARPC3_HUMAN | ↓* | 2.43 | 5.84 |
| Actin-related protein 3 | ARP3_HUMAN | → | 14.48 | 15.26 |
| Allograft inflammatory factor 1 | AIF1_HUMAN | ↓ | 2.15 | 3.77 |
| Annexin A1 | ANXA1_HUMAN | → | 33.67 | 34.03 |
| Annexin A11 | ANX11_HUMAN | → | 8.81 | 9.12 |
| Annexin A3 | ANXA3_HUMAN | ↑ | 4.67 | not identified |
| Azurocidin | CAP7_HUMAN | ↓ | 25.02 | 33.53 |
| Cell division control protein 42 homolog | CDC42_HUMAN | ↓* | 5.34 | 9.38 |
| Coronin-1A | COR1A_HUMAN | ↓ | 22.21 | 26.98 |
| Coronin-1C | COR1C_HUMAN | ↓ | 4.11 | 5.75 |
| Dynamin-2 | DYN2_HUMAN | ↓ | not identified | 3.86 |
| Gelsolin | GELS_HUMAN | → | 9.41 | 8.55 |
| Heat shock protein HSP 90-alpha | HS90A_HUMAN | → | 8.84 | 8.76 |
| Immunoglobulin kappa constant | IGKC_HUMAN | ↓ | not identified | 2.26 |
| Integrin alpha-M | ITAM_HUMAN | ↓* | 33.59 | 40.66 |
| Integrin beta-2 | ITB2_HUMAN | ↓* | 32.01 | 45.55 |
| Myeloblastin | PRTN3_HUMAN | ↓ | 23.44 | 27.27 |
| Myosin-9 | MYH9_HUMAN | ↓ | 129.68 | 147.04 |
| Neutrophil cytosol factor 2 | NCF2_HUMAN | → | 5.40 | 5.76 |
| Neutrophil cytosol factor 4 | NCF4_HUMAN | ↓ | not identified | 2.35 |
| Neutrophil elastase | ELNE_HUMAN | → | 56.21 | 54.89 |
| Sorting nexin-3 | SNX3_HUMAN | ↓ | not identified | 1.67 |
