# Supplementary material for: A Secreted Bacterial Peptidylarginine Deiminase Can Neutralize Human Innate Immune Defenses
Source: mBio. 2018 Oct 30;9(5):e01704-18. doi: 10.1128/mBio.01704-18 (PMC6212822; doi:10.1128/mBio.01704-18)
Supplement: FIG S5 [file mbo005184137sf5.pdf]

Supplementary Figure S5

Histone H3

Uniprot ID: P68431

Citrullinated peptides identified when incubated with PPAD

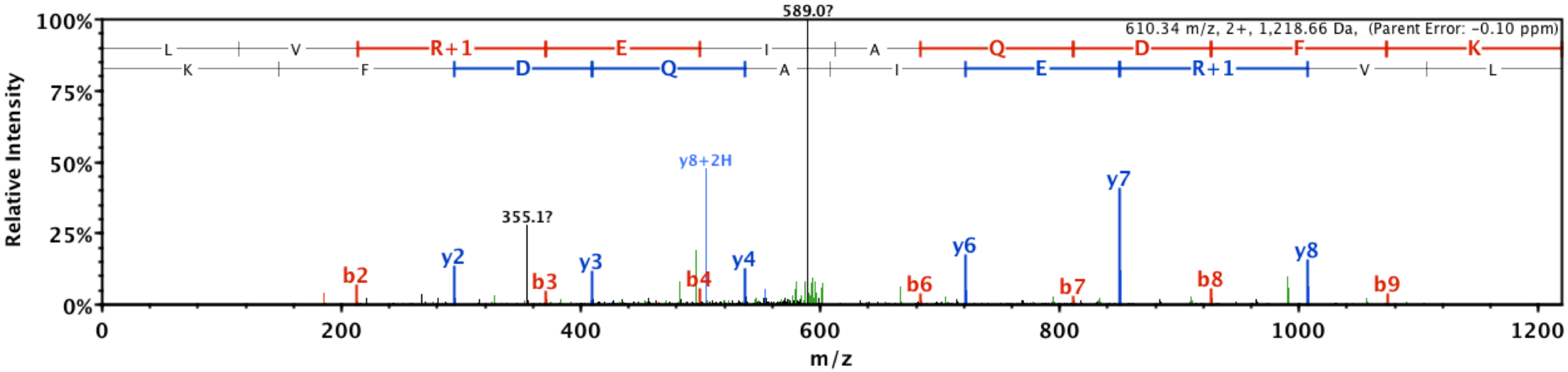

B	B Ions	B+2H	B-NH3	B-H2O	AA	Y Ions	Y+2H	Y-NH3	Y-H2O	Y
1	114.1				L	1,219.7	610.3	1,202.6	1,201.7	10
2	213.2				V	1,106.6	553.8	1,089.6	1,088.6	9
3	370.2	185.6	353.2		R+1	1,007.5	504.3	990.5	989.5	8
4	499.3	250.1	482.3	481.3	E	850.4	425.7	833.4	832.4	7
5	612.4	306.7	595.3	594.4	I	721.4	361.2	704.4	703.4	6
6	683.4	342.2	666.4	665.4	A	608.3		591.3	590.3	5
7	811.5	406.2	794.4	793.5	Q	537.3		520.2	519.3	4
8	926.5	463.8	909.5	908.5	D	409.2		392.2	391.2	3
9	1,073.6	537.3	1,056.5	1,055.6	F	294.2		277.2		2
10	1,219.7	610.3	1,202.6	1,201.7	K	147.1		130.1		1

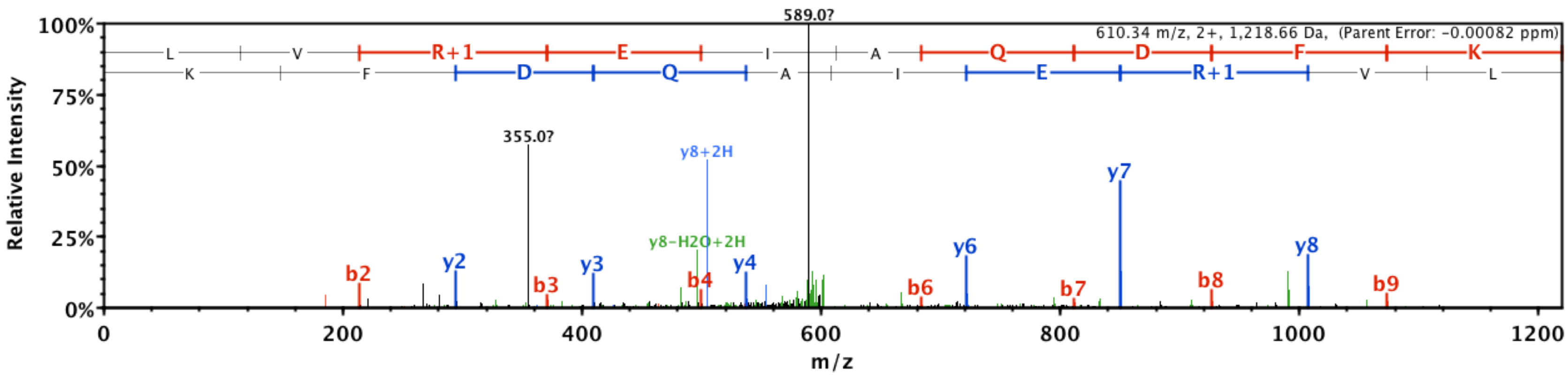

B	B Ions	B+2H	B-NH3	B-H2O	AA	Y Ions	Y+2H	Y-NH3	Y-H2O	Y
1	114.1				L	1,219.7	610.3	1,202.6	1,201.7	10
2	213.2				V	1,106.6	553.8	1,089.6	1,088.6	9
3	370.2	185.6	353.2		R+1	1,007.5	504.3	990.5	989.5	8
4	499.3	250.1	482.3	481.3	E	850.4	425.7	833.4	832.4	7
5	612.4	306.7	595.3	594.4	I	721.4	361.2	704.4	703.4	6
6	683.4	342.2	666.4	665.4	A	608.3		591.3	590.3	5
7	811.5	406.2	794.4	793.5	Q	537.3		520.2	519.3	4
8	926.5	463.8	909.5	908.5	D	409.2		392.2	391.2	3
9	1,073.6	537.3	1,056.5	1,055.6	F	294.2		277.2		2
10	1,219.7	610.3	1,202.6	1,201.7	K	147.1		130.1		1

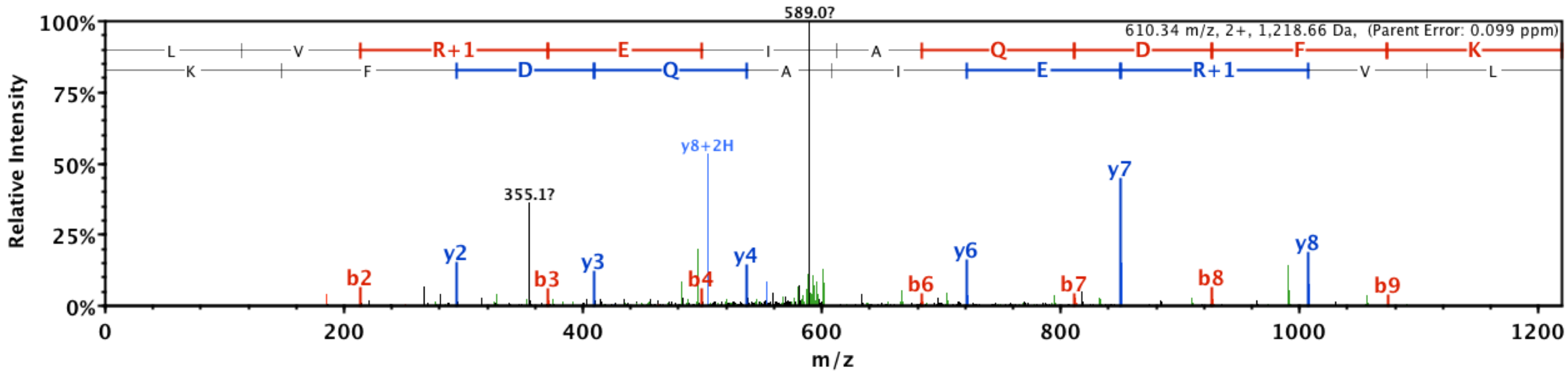

B	B Ions	B+2H	B-NH3	B-H2O	AA	Y Ions	Y+2H	Y-NH3	Y-H2O	Y
1	114.1				L	1,219.7	610.3	1,202.6	1,201.7	10
2	213.2				V	1,106.6	553.8	1,089.6	1,088.6	9
3	370.2	185.6	353.2		R+1	1,007.5	504.3	990.5	989.5	8
4	499.3	250.1	482.3	481.3	E	850.4	425.7	833.4	832.4	7
5	612.4	306.7	595.3	594.4	I	721.4	361.2	704.4	703.4	6
6	683.4	342.2	666.4	665.4	A	608.3		591.3	590.3	5
7	811.5	406.2	794.4	793.5	Q	537.3		520.2	519.3	4
8	926.5	463.8	909.5	908.5	D	409.2		392.2	391.2	3
9	1,073.6	537.3	1,056.5	1,055.6	F	294.2		277.2		2
10	1,219.7	610.3	1,202.6	1,201.7	K	147.1		130.1		1

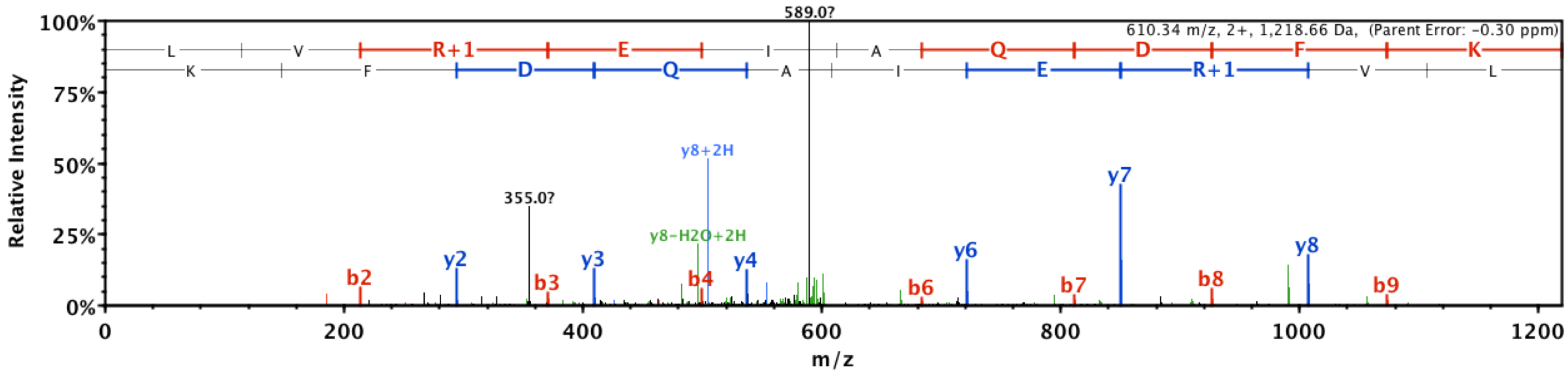

B	B Ions	B+2H	B-NH3	B-H2O	AA	Y Ions	Y+2H	Y-NH3	Y-H2O	Y
1	114.1				L	1,219.7	610.3	1,202.6	1,201.7	10
2	213.2				V	1,106.6	553.8	1,089.6	1,088.6	9
3	370.2	185.6	353.2		R+1	1,007.5	504.3	990.5	989.5	8
4	499.3	250.1	482.3	481.3	E	850.4	425.7	833.4	832.4	7
5	612.4	306.7	595.3	594.4	I	721.4	361.2	704.4	703.4	6
6	683.4	342.2	666.4	665.4	A	608.3		591.3	590.3	5
7	811.5	406.2	794.4	793.5	Q	537.3		520.2	519.3	4
8	926.5	463.8	909.5	908.5	D	409.2		392.2	391.2	3
9	1,073.6	537.3	1,056.5	1,055.6	F	294.2		277.2		2
10	1,219.7	610.3	1,202.6	1,201.7	K	147.1		130.1		1

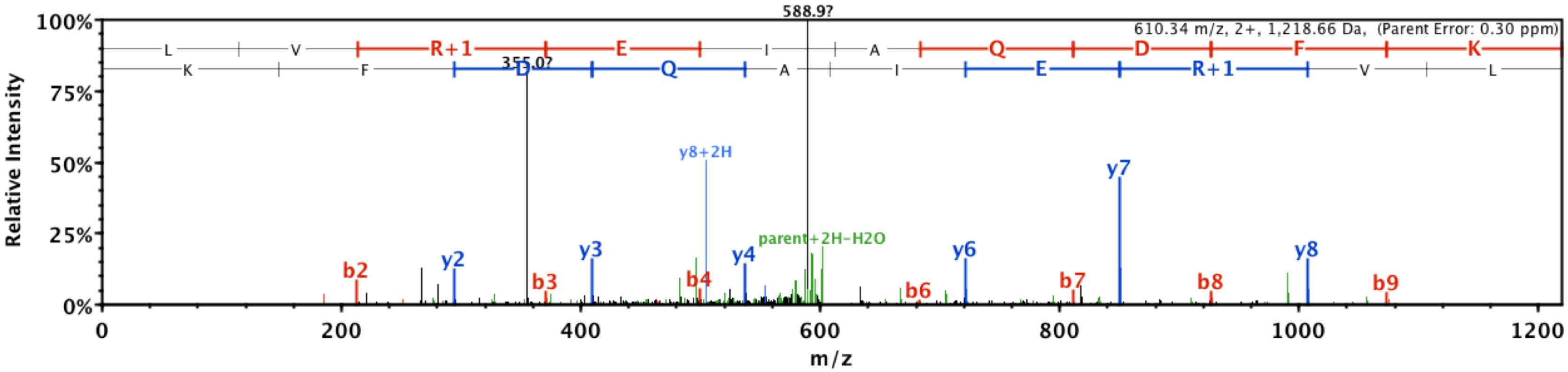

B	B Ions	B+2H	B-NH3	B-H2O	AA	Y Ions	Y+2H	Y-NH3	Y-H2O	Y
1	114.1				L	1,219.7	610.3	1,202.6	1,201.7	10
2	213.2				V	1,106.6	553.8	1,089.6	1,088.6	9
3	370.2	185.6	353.2		R+1	1,007.5	504.3	990.5	989.5	8
4	499.3	250.1	482.3	481.3	E	850.4	425.7	833.4	832.4	7
5	612.4	306.7	595.3	594.4	I	721.4	361.2	704.4	703.4	6
6	683.4	342.2	666.4	665.4	A	608.3		591.3	590.3	5
7	811.5	406.2	794.4	793.5	Q	537.3		520.2	519.3	4
8	926.5	463.8	909.5	908.5	D	409.2		392.2	391.2	3
9	1,073.6	537.3	1,056.5	1,055.6	F	294.2		277.2		2
10	1,219.7	610.3	1,202.6	1,201.7	K	147.1		130.1		1

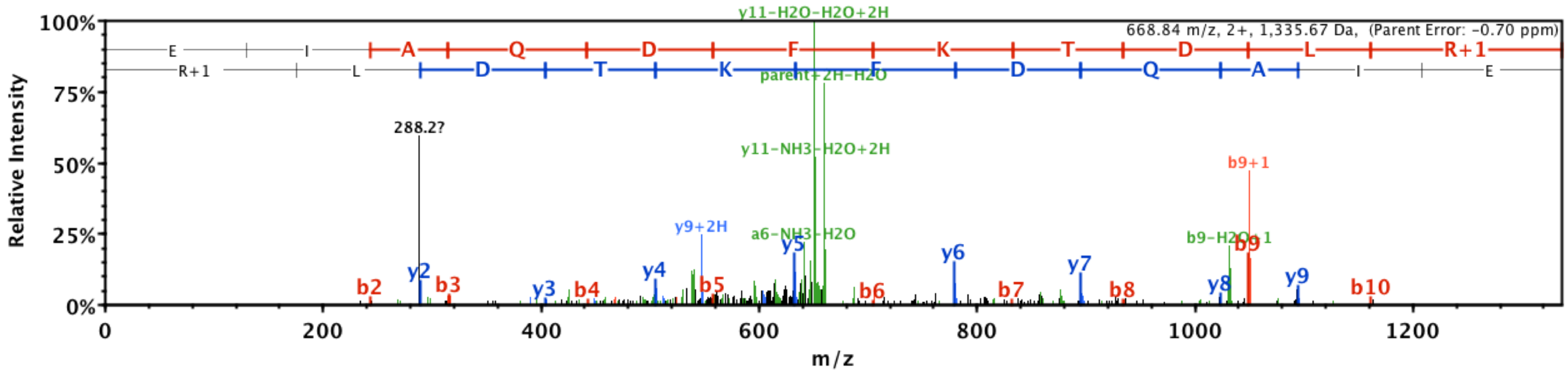

B	B Ions	B+2H	B-NH3	B-H2O	AA	Y Ions	Y+2H	Y-NH3	Y-H2O	Y
1	130.0			112.0	E	1,336.7	668.8	1,319.6	1,318.7	11
2	243.1			225.1	I	1,207.6	604.3	1,190.6	1,189.6	10
3	314.2			296.2	A	1,094.5	547.8	1,077.5	1,076.5	9
4	442.2		425.2	424.2	Q	1,023.5	512.3	1,006.5	1,005.5	8
5	557.3		540.2	539.2	D	895.5	448.2	878.4	877.4	7
6	704.3	352.7	687.3	686.3	F	780.4	390.7	763.4	762.4	6
7	832.4	416.7	815.4	814.4	K	633.4	317.2	616.3	615.3	5
8	933.5	467.2	916.4	915.5	T	505.3		488.2	487.3	4
9	1,048.5	524.8	1,031.5	1,030.5	D	404.2		387.2	386.2	3
10	1,161.6	581.3	1,144.6	1,143.6	L	289.2		272.2		2
11	1,336.7	668.8	1,319.6	1,318.7	R+1	176.1		159.1		1

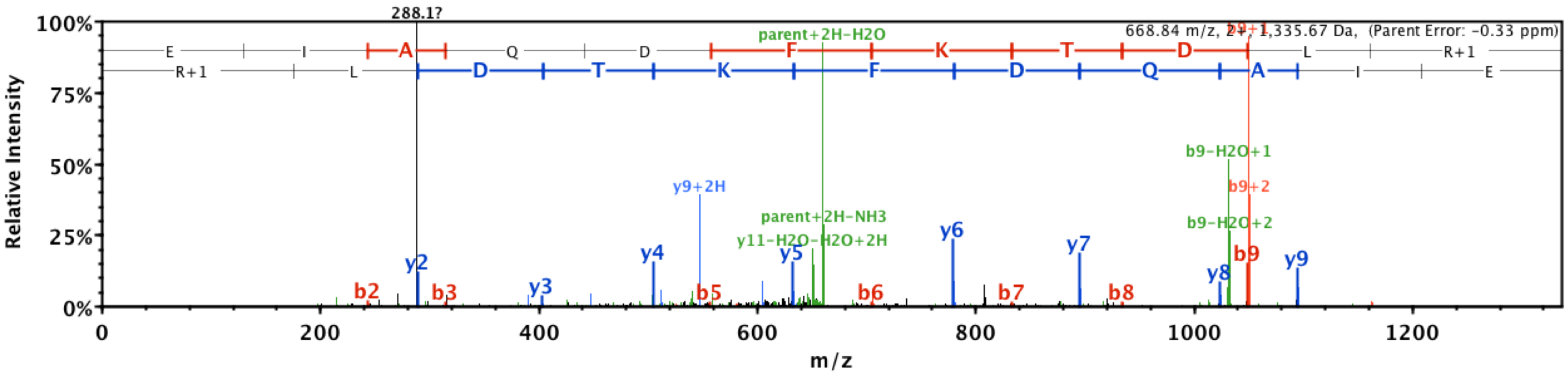

B	B Ions	B+2H	B-NH3	B-H2O	AA	Y Ions	Y+2H	Y-NH3	Y-H2O	Y
1	130.0			112.0	E	1,336.7	668.8	1,319.6	1,318.7	11
2	243.1			225.1	I	1,207.6	604.3	1,190.6	1,189.6	10
3	314.2			296.2	A	1,094.5	547.8	1,077.5	1,076.5	9
4	442.2		425.2	424.2	Q	1,023.5	512.3	1,006.5	1,005.5	8
5	557.3		540.2	539.2	D	895.5	448.2	878.4	877.4	7
6	704.3	352.7	687.3	686.3	F	780.4	390.7	763.4	762.4	6
7	832.4	416.7	815.4	814.4	K	633.4	317.2	616.3	615.3	5
8	933.5	467.2	916.4	915.5	T	505.3		488.2	487.3	4
9	1,048.5	524.8	1,031.5	1,030.5	D	404.2		387.2	386.2	3
10	1,161.6	581.3	1,144.6	1,143.6	L	289.2		272.2		2
11	1,336.7	668.8	1,319.6	1,318.7	R+1	176.1		159.1		1

Histone H3

Uniprot ID: P68431

Citrullinated peptides identified when incubated with human PAD2

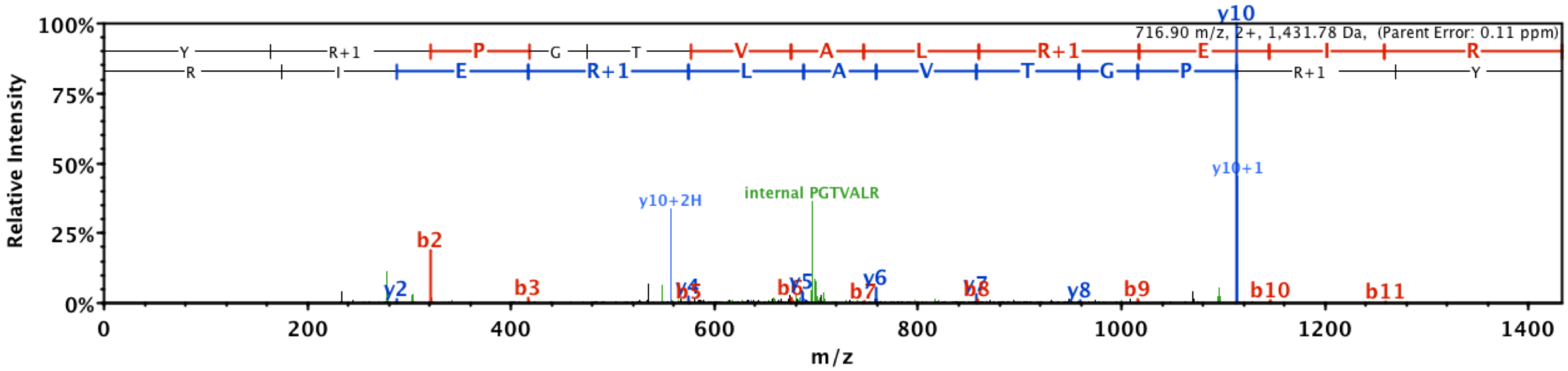

B	B Ions	B+2H	B-NH3	B-H2O	AA	Y Ions	Y+2H	Y-NH3	Y-H2O	Y
1	164.1			146.1	Y	1,432.8	716.9	1,415.8	1,414.8	12
2	321.2	161.1	304.1	303.1	R+1	1,269.7	635.4	1,252.7	1,251.7	11
3	418.2	209.6	401.2	400.2	P	1,112.6	556.8	1,095.6	1,094.6	10
4	475.2	238.1	458.2	457.2	G	1,015.6	508.3	998.6	997.6	9
5	576.3	288.6	559.3	558.3	T	958.6	479.8	941.5	940.6	8
6	675.3	338.2	658.3	657.3	V	857.5	429.3	840.5	839.5	7
7	746.4	373.7	729.4	728.4	A	758.5	379.7	741.4	740.4	6
8	859.5	430.2	842.4	841.5	L	687.4	344.2	670.4	669.4	5
9	1,016.6	508.8	999.5	998.5	R+1	574.3	287.7	557.3	556.3	4
10	1,145.6	573.3	1,128.6	1,127.6	E	417.2		400.2	399.2	3
11	1,258.7	629.8	1,241.7	1,240.7	I	288.2		271.2	270.2	2
12	1,432.8	716.9	1,415.8	1,414.8	R	175.1		158.1	157.1	1

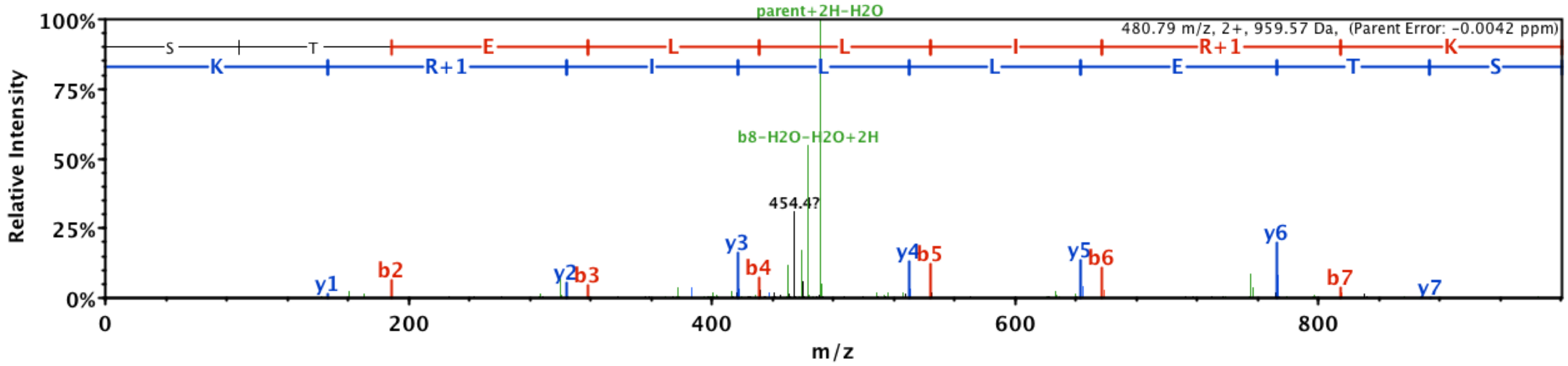

B	B Ions	B+2H	B-NH3	B-H2O	AA	Y Ions	Y+2H	Y-NH3	Y-H2O	Y
1	88.0			70.0	S	960.6	480.8	943.5	942.6	8
2	189.1			171.1	T	873.5	437.3	856.5	855.5	7
3	318.1			300.1	E	772.5	386.8	755.5	754.5	6
4	431.2			413.2	L	643.5	322.2	626.4		5
5	544.3			526.3	L	530.4	265.7	513.3		4
6	657.4	329.2		639.4	I	417.3	209.1	400.3		3
7	814.5	407.7	797.4	796.5	R+1	304.2	152.6	287.2		2
8	960.6	480.8	943.5	942.6	K	147.1		130.1		1

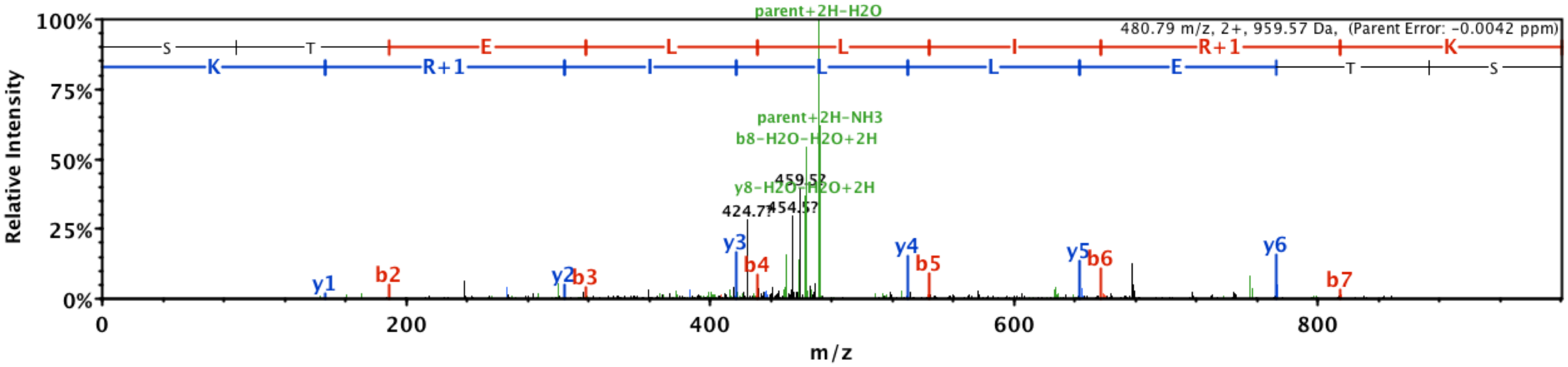

B	B Ions	B+2H	B-NH3	B-H2O	AA	Y Ions	Y+2H	Y-NH3	Y-H2O	Y
1	88.0			70.0	S	960.6	480.8	943.5	942.6	8
2	189.1			171.1	T	873.5	437.3	856.5	855.5	7
3	318.1			300.1	E	772.5	386.8	755.5	754.5	6
4	431.2			413.2	L	643.5	322.2	626.4		5
5	544.3			526.3	L	530.4	265.7	513.3		4
6	657.4	329.2		639.4	I	417.3	209.1	400.3		3
7	814.5	407.7	797.4	796.5	R+1	304.2	152.6	287.2		2
8	960.6	480.8	943.5	942.6	K	147.1		130.1		1

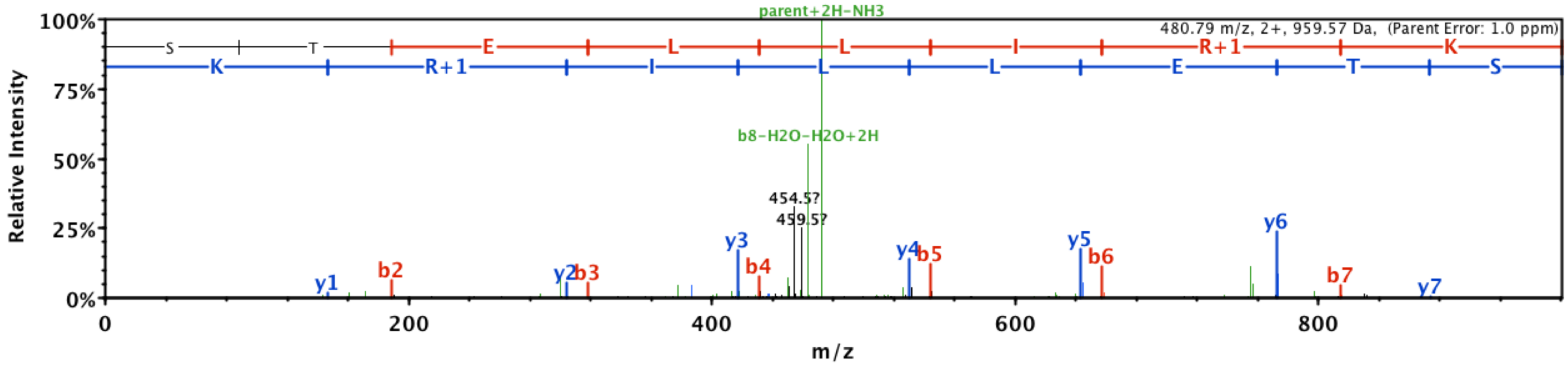

B	B Ions	B+2H	B-NH3	B-H2O	AA	Y Ions	Y+2H	Y-NH3	Y-H2O	Y
1	88.0			70.0	S	960.6	480.8	943.5	942.6	8
2	189.1			171.1	T	873.5	437.3	856.5	855.5	7
3	318.1			300.1	E	772.5	386.8	755.5	754.5	6
4	431.2			413.2	L	643.5	322.2	626.4		5
5	544.3			526.3	L	530.4	265.7	513.3		4
6	657.4	329.2		639.4	I	417.3	209.1	400.3		3
7	814.5	407.7	797.4	796.5	R+1	304.2	152.6	287.2		2
8	960.6	480.8	943.5	942.6	K	147.1		130.1		1

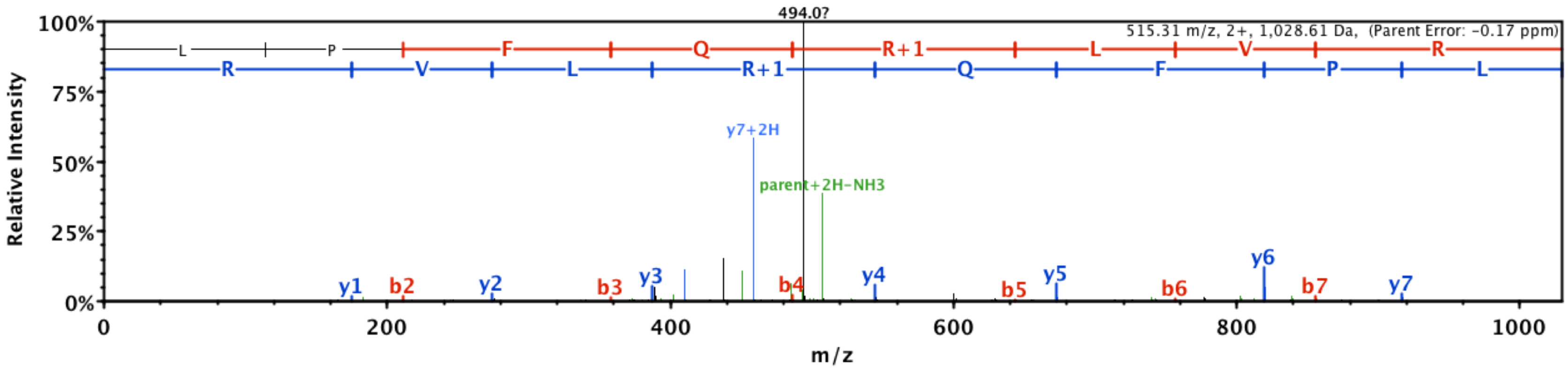

B	B Ions	B+2H	B-NH3	B-H2O	AA	Y Ions	Y+2H	Y-NH3	Y-H2O	Y
1	114.1				L	1,029.6	515.3	1,012.6		8
2	211.1				P	916.5	458.8	899.5		7
3	358.2				F	819.5	410.2	802.5		6
4	486.3		469.2		Q	672.4	336.7	655.4		5
5	643.4	322.2	626.3		R+1	544.4	272.7	527.3		4
6	756.4	378.7	739.4		L	387.3		370.2		3
7	855.5	428.3	838.5		V	274.2		257.2		2
8	1,029.6	515.3	1,012.6		R	175.1		158.1		1

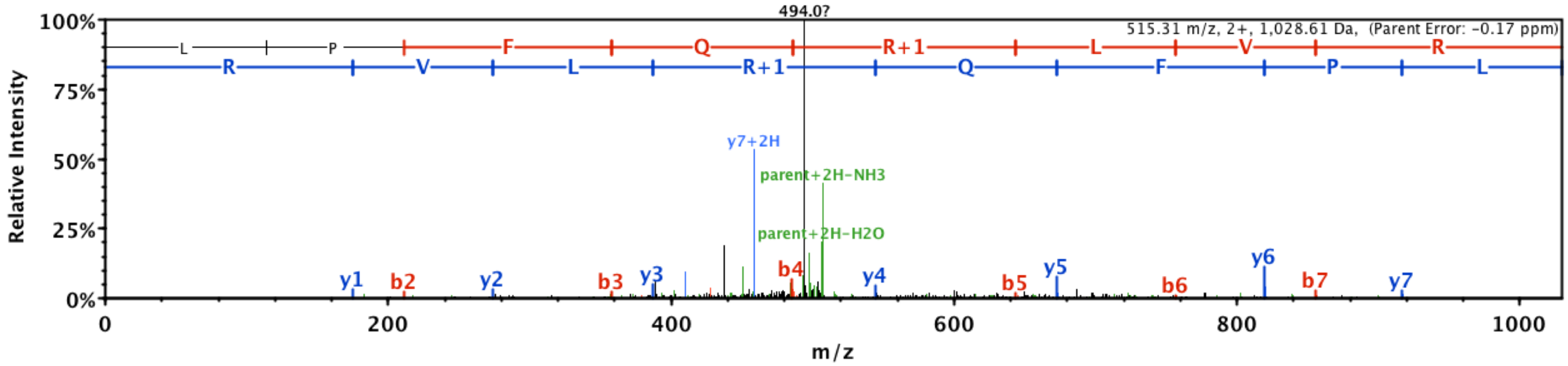

B	B Ions	B+2H	B-NH3	B-H2O	AA	Y Ions	Y+2H	Y-NH3	Y-H2O	Y
1	114.1				L	1,029.6	515.3	1,012.6		8
2	211.1				P	916.5	458.8	899.5		7
3	358.2				F	819.5	410.2	802.5		6
4	486.3		469.2		Q	672.4	336.7	655.4		5
5	643.4	322.2	626.3		R+1	544.4	272.7	527.3		4
6	756.4	378.7	739.4		L	387.3		370.2		3
7	855.5	428.3	838.5		V	274.2		257.2		2
8	1,029.6	515.3	1,012.6		R	175.1		158.1		1

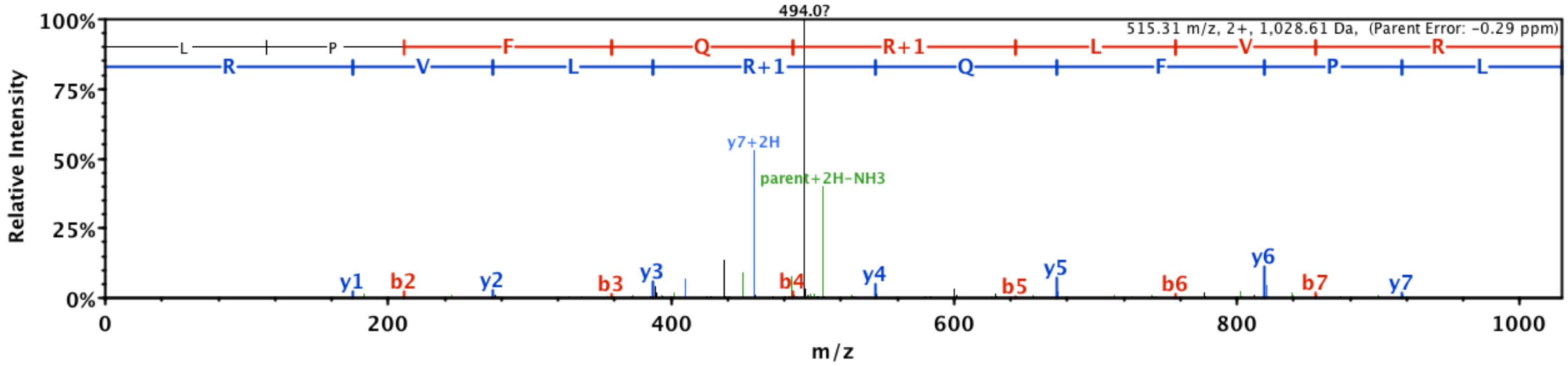

B	B Ions	B+2H	B-NH3	B-H2O	AA	Y Ions	Y+2H	Y-NH3	Y-H2O	Y
1	114.1				L	1,029.6	515.3	1,012.6		8
2	211.1				P	916.5	458.8	899.5		7
3	358.2				F	819.5	410.2	802.5		6
4	486.3		469.2		Q	672.4	336.7	655.4		5
5	643.4	322.2	626.3		R+1	544.4	272.7	527.3		4
6	756.4	378.7	739.4		L	387.3		370.2		3
7	855.5	428.3	838.5		V	274.2		257.2		2
8	1,029.6	515.3	1,012.6		R	175.1		158.1		1

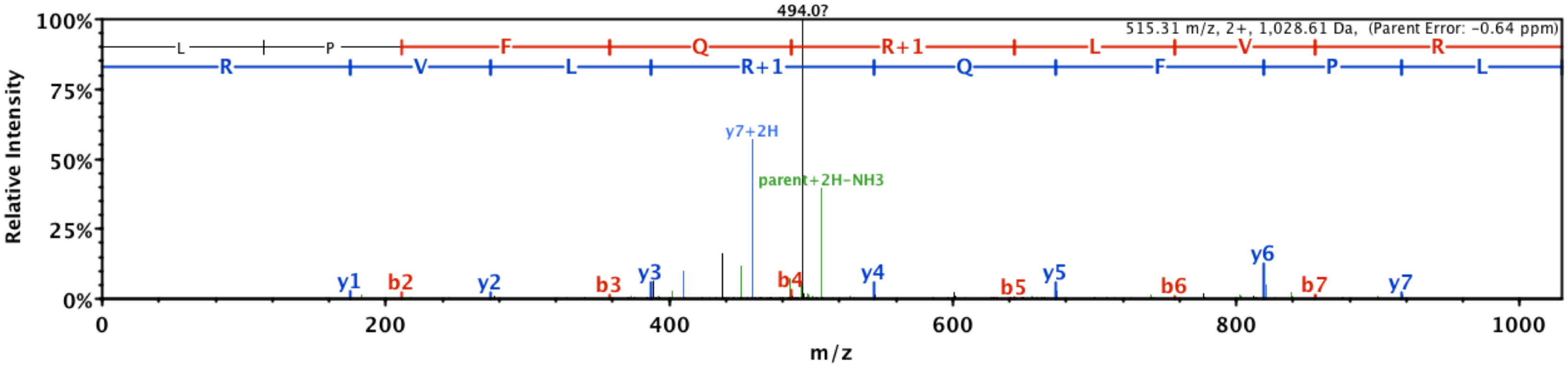

B	B Ions	B+2H	B-NH3	B-H2O	AA	Y Ions	Y+2H	Y-NH3	Y-H2O	Y
1	114.1				L	1,029.6	515.3	1,012.6		8
2	211.1				P	916.5	458.8	899.5		7
3	358.2				F	819.5	410.2	802.5		6
4	486.3		469.2		Q	672.4	336.7	655.4		5
5	643.4	322.2	626.3		R+1	544.4	272.7	527.3		4
6	756.4	378.7	739.4		L	387.3		370.2		3
7	855.5	428.3	838.5		V	274.2		257.2		2
8	1,029.6	515.3	1,012.6		R	175.1		158.1		1

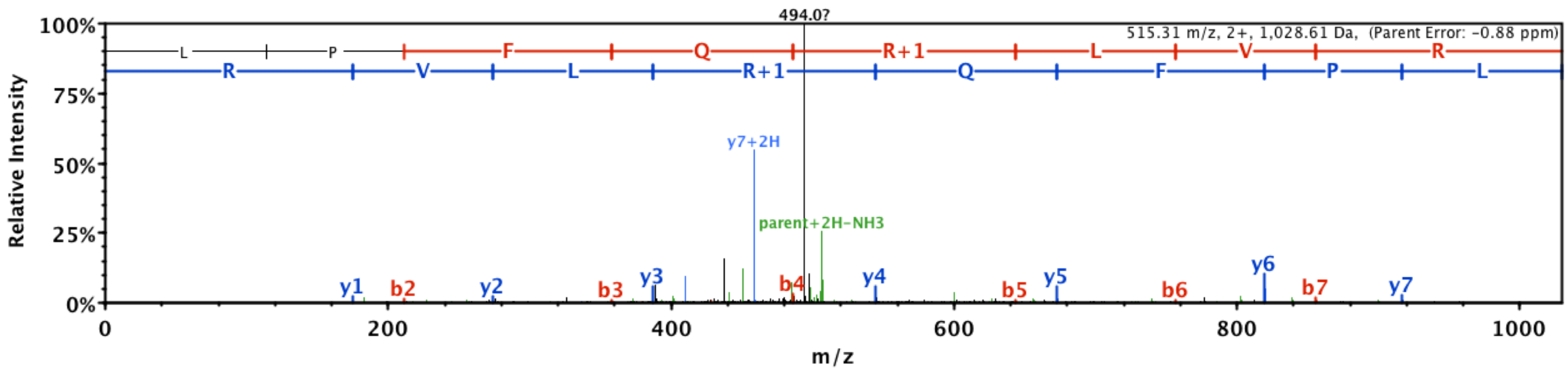

B	B Ions	B+2H	B-NH3	B-H2O	AA	Y Ions	Y+2H	Y-NH3	Y-H2O	Y
1	114.1				L	1,029.6	515.3	1,012.6		8
2	211.1				P	916.5	458.8	899.5		7
3	358.2				F	819.5	410.2	802.5		6
4	486.3		469.2		Q	672.4	336.7	655.4		5
5	643.4	322.2	626.3		R+1	544.4	272.7	527.3		4
6	756.4	378.7	739.4		L	387.3		370.2		3
7	855.5	428.3	838.5		V	274.2		257.2		2
8	1,029.6	515.3	1,012.6		R	175.1		158.1		1

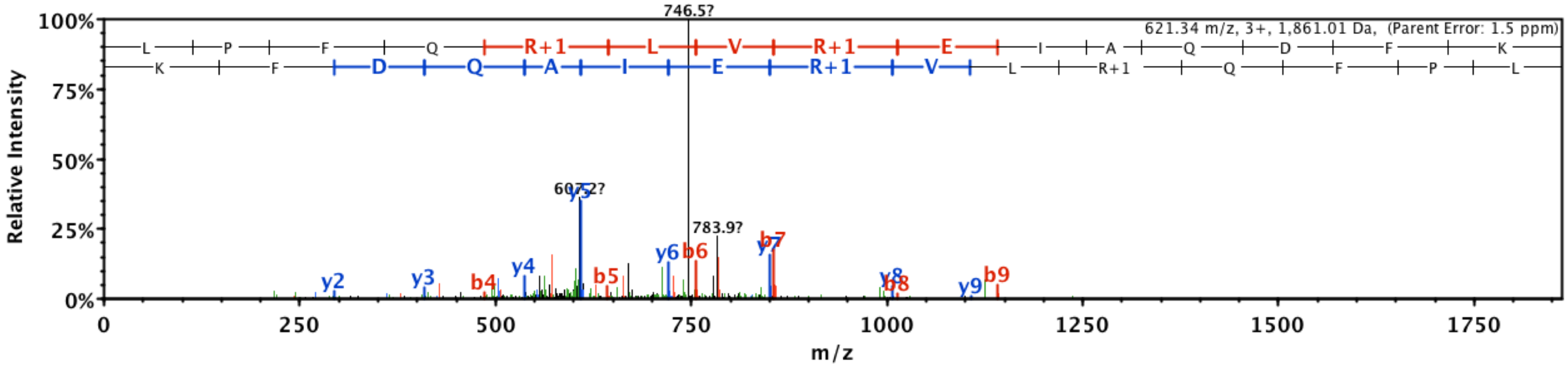

B	B Ions	B+2H	B-NH3	B-H2O	AA	Y Ions	Y+2H	Y-NH3	Y-H2O	Y
1	114.1	57.5			L	1,862.0	931.5	1,845.0	1,844.0	15
2	211.1	106.1			P	1,748.9	875.0	1,731.9	1,730.9	14
3	358.2	179.6			F	1,651.9	826.4	1,634.9	1,633.9	13
4	486.3	243.6	469.2		Q	1,504.8	752.9	1,487.8	1,486.8	12
5	643.4	322.2	626.3		R+1	1,376.8	688.9	1,359.7	1,358.7	11
6	756.4	378.7	739.4		L	1,219.7	610.3	1,202.6	1,201.7	10
7	855.5	428.3	838.5		V	1,106.6	553.8	1,089.6	1,088.6	9
8	1,012.6	506.8	995.6		R+1	1,007.5	504.3	990.5	989.5	8
9	1,141.6	571.3	1,124.6	1,123.6	E	850.4	425.7	833.4	832.4	7
10	1,254.7	627.9	1,237.7	1,236.7	I	721.4	361.2	704.4	703.4	6
11	1,325.8	663.4	1,308.7	1,307.7	A	608.3	304.7	591.3	590.3	5
12	1,453.8	727.4	1,436.8	1,435.8	Q	537.3	269.1	520.2	519.3	4
13	1,568.8	784.9	1,551.8	1,550.8	D	409.2	205.1	392.2	391.2	3
14	1,715.9	858.5	1,698.9	1,697.9	F	294.2	147.6	277.2		2
15	1,862.0	931.5	1,845.0	1,844.0	K	147.1	74.1	130.1		1

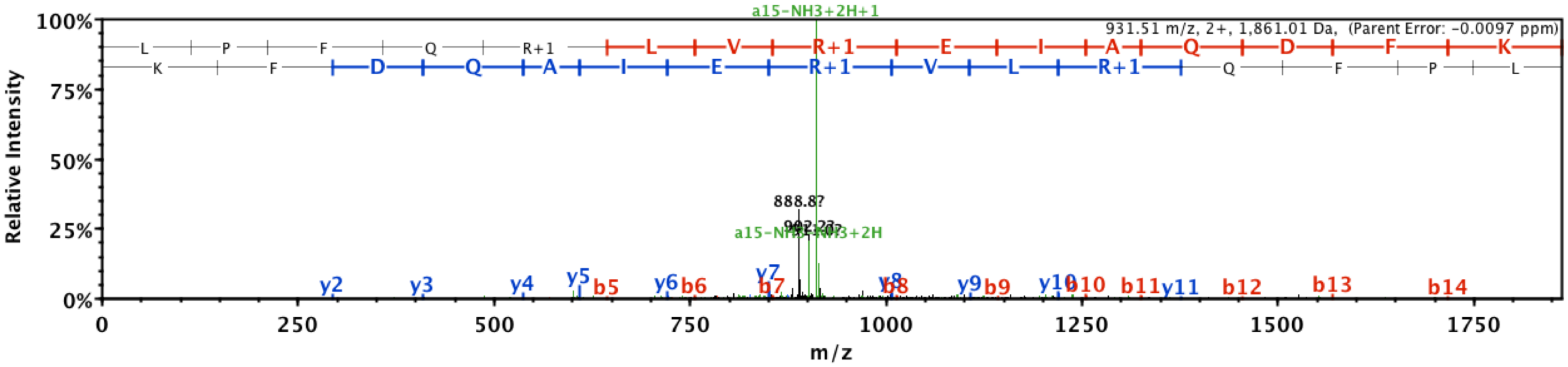

B	B Ions	B+2H	B-NH3	B-H2O	AA	Y Ions	Y+2H	Y-NH3	Y-H2O	Y
1	114.1				L	1,862.0	931.5	1,845.0	1,844.0	15
2	211.1				P	1,748.9	875.0	1,731.9	1,730.9	14
3	358.2				F	1,651.9	826.4	1,634.9	1,633.9	13
4	486.3		469.2		Q	1,504.8	752.9	1,487.8	1,486.8	12
5	643.4	322.2	626.3		R+1	1,376.8	688.9	1,359.7	1,358.7	11
6	756.4	378.7	739.4		L	1,219.7	610.3	1,202.6	1,201.7	10
7	855.5	428.3	838.5		V	1,106.6	553.8	1,089.6	1,088.6	9
8	1,012.6	506.8	995.6		R+1	1,007.5	504.3	990.5	989.5	8
9	1,141.6	571.3	1,124.6	1,123.6	E	850.4	425.7	833.4	832.4	7
10	1,254.7	627.9	1,237.7	1,236.7	I	721.4	361.2	704.4	703.4	6
11	1,325.8	663.4	1,308.7	1,307.7	A	608.3		591.3	590.3	5
12	1,453.8	727.4	1,436.8	1,435.8	Q	537.3		520.2	519.3	4
13	1,568.8	784.9	1,551.8	1,550.8	D	409.2		392.2	391.2	3
14	1,715.9	858.5	1,698.9	1,697.9	F	294.2		277.2		2
15	1,862.0	931.5	1,845.0	1,844.0	K	147.1		130.1		1

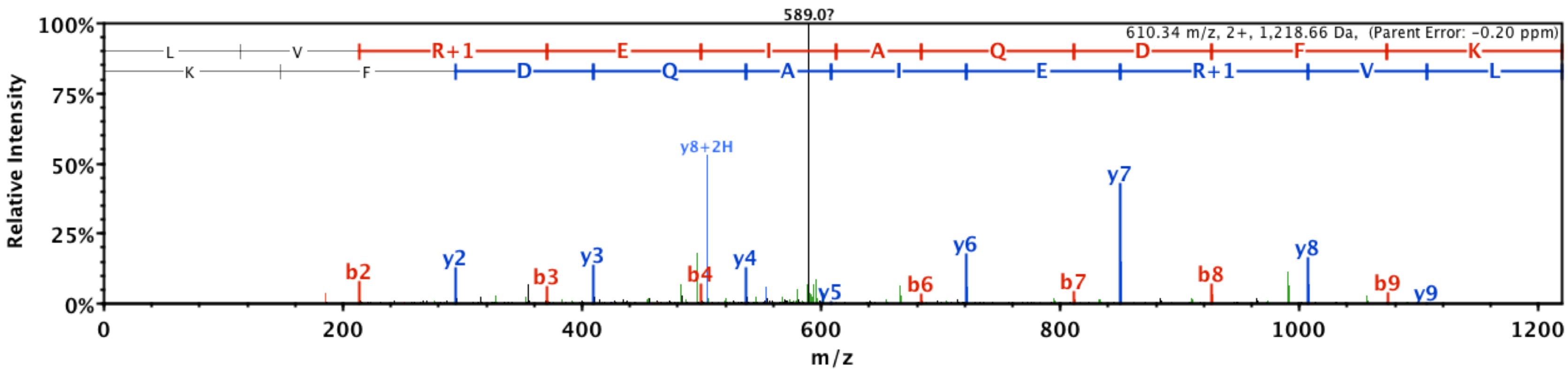

B	B Ions	B+2H	B-NH3	B-H2O	AA	Y Ions	Y+2H	Y-NH3	Y-H2O	Y
1	114.1				L	1,219.7	610.3	1,202.6	1,201.7	10
2	213.2				V	1,106.6	553.8	1,089.6	1,088.6	9
3	370.2	185.6	353.2		R+1	1,007.5	504.3	990.5	989.5	8
4	499.3	250.1	482.3	481.3	E	850.4	425.7	833.4	832.4	7
5	612.4	306.7	595.3	594.4	I	721.4	361.2	704.4	703.4	6
6	683.4	342.2	666.4	665.4	A	608.3		591.3	590.3	5
7	811.5	406.2	794.4	793.5	Q	537.3		520.2	519.3	4
8	926.5	463.8	909.5	908.5	D	409.2		392.2	391.2	3
9	1,073.6	537.3	1,056.5	1,055.6	F	294.2		277.2		2
10	1,219.7	610.3	1,202.6	1,201.7	K	147.1		130.1		1

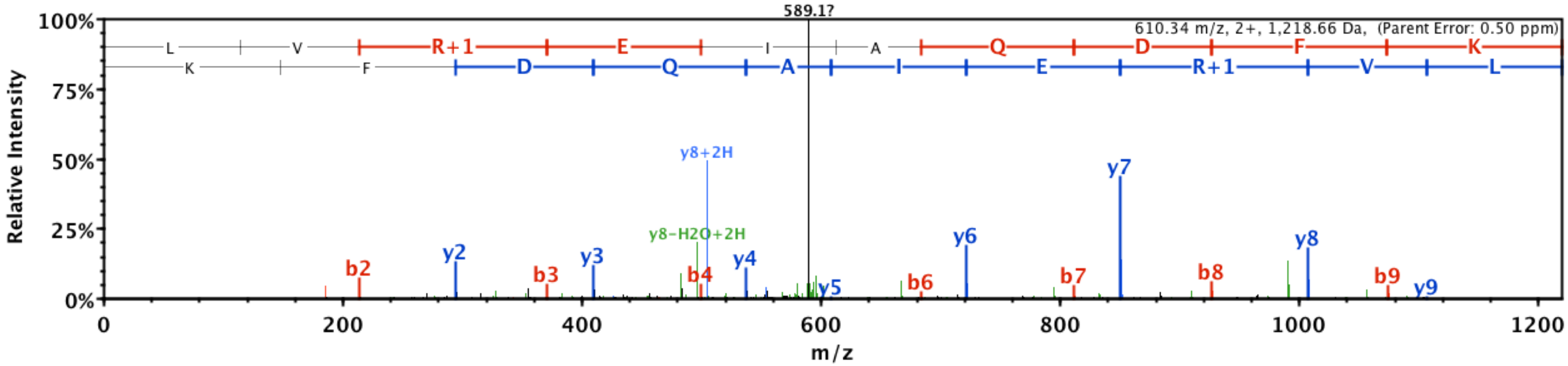

B	B Ions	B+2H	B-NH3	B-H2O	AA	Y Ions	Y+2H	Y-NH3	Y-H2O	Y
1	114.1				L	1,219.7	610.3	1,202.6	1,201.7	10
2	213.2				V	1,106.6	553.8	1,089.6	1,088.6	9
3	370.2	185.6	353.2		R+1	1,007.5	504.3	990.5	989.5	8
4	499.3	250.1	482.3	481.3	E	850.4	425.7	833.4	832.4	7
5	612.4	306.7	595.3	594.4	I	721.4	361.2	704.4	703.4	6
6	683.4	342.2	666.4	665.4	A	608.3		591.3	590.3	5
7	811.5	406.2	794.4	793.5	Q	537.3		520.2	519.3	4
8	926.5	463.8	909.5	908.5	D	409.2		392.2	391.2	3
9	1,073.6	537.3	1,056.5	1,055.6	F	294.2		277.2		2
10	1,219.7	610.3	1,202.6	1,201.7	K	147.1		130.1		1

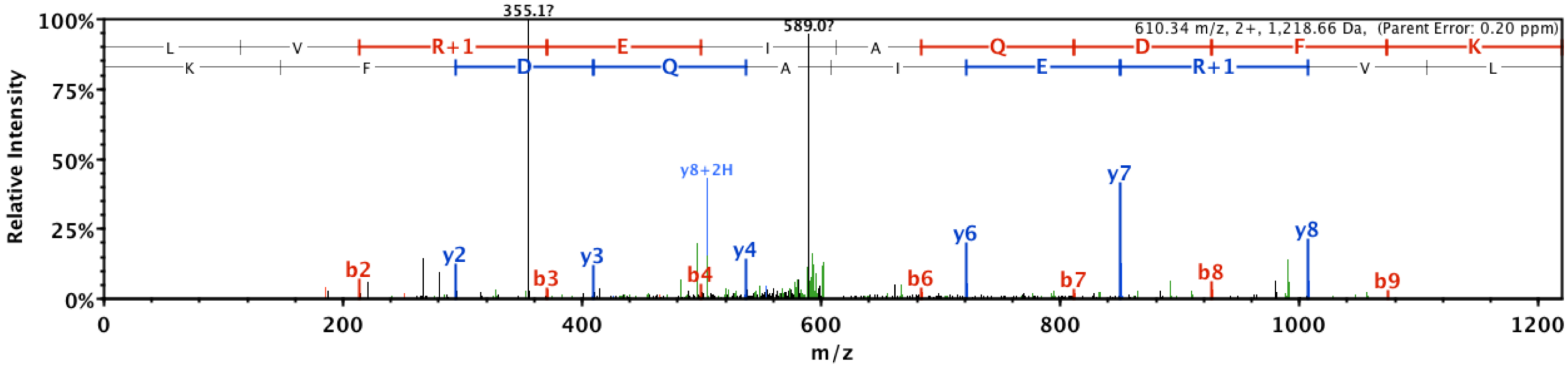

B	B Ions	B+2H	B-NH3	B-H2O	AA	Y Ions	Y+2H	Y-NH3	Y-H2O	Y
1	114.1				L	1,219.7	610.3	1,202.6	1,201.7	10
2	213.2				V	1,106.6	553.8	1,089.6	1,088.6	9
3	370.2	185.6	353.2		R+1	1,007.5	504.3	990.5	989.5	8
4	499.3	250.1	482.3	481.3	E	850.4	425.7	833.4	832.4	7
5	612.4	306.7	595.3	594.4	I	721.4	361.2	704.4	703.4	6
6	683.4	342.2	666.4	665.4	A	608.3		591.3	590.3	5
7	811.5	406.2	794.4	793.5	Q	537.3		520.2	519.3	4
8	926.5	463.8	909.5	908.5	D	409.2		392.2	391.2	3
9	1,073.6	537.3	1,056.5	1,055.6	F	294.2		277.2		2
10	1,219.7	610.3	1,202.6	1,201.7	K	147.1		130.1		1

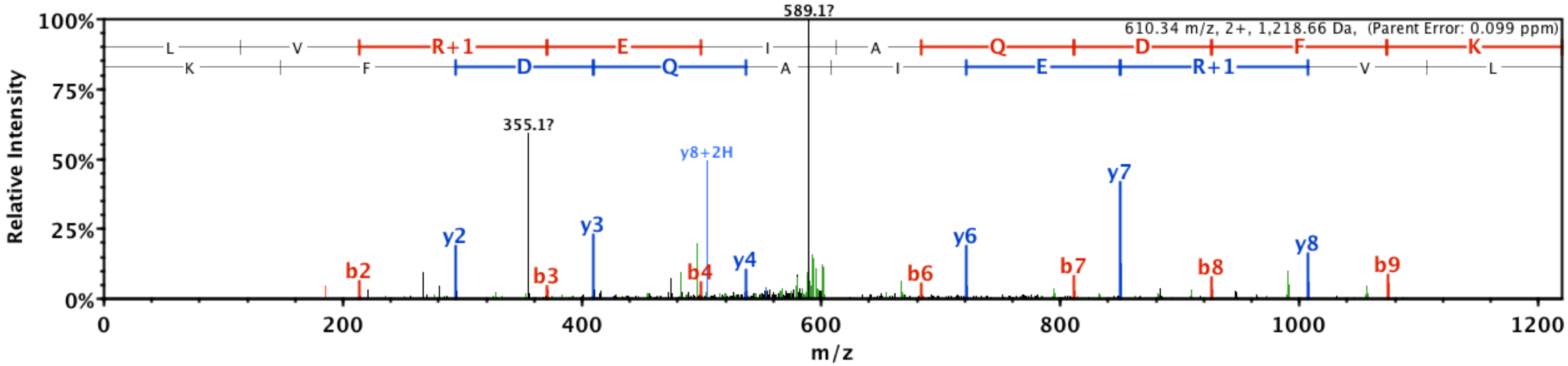

B	B Ions	B+2H	B-NH3	B-H2O	AA	Y Ions	Y+2H	Y-NH3	Y-H2O	Y
1	114.1				L	1,219.7	610.3	1,202.6	1,201.7	10
2	213.2				V	1,106.6	553.8	1,089.6	1,088.6	9
3	370.2	185.6	353.2		R+1	1,007.5	504.3	990.5	989.5	8
4	499.3	250.1	482.3	481.3	E	850.4	425.7	833.4	832.4	7
5	612.4	306.7	595.3	594.4	I	721.4	361.2	704.4	703.4	6
6	683.4	342.2	666.4	665.4	A	608.3		591.3	590.3	5
7	811.5	406.2	794.4	793.5	Q	537.3		520.2	519.3	4
8	926.5	463.8	909.5	908.5	D	409.2		392.2	391.2	3
9	1,073.6	537.3	1,056.5	1,055.6	F	294.2		277.2		2
10	1,219.7	610.3	1,202.6	1,201.7	K	147.1		130.1		1

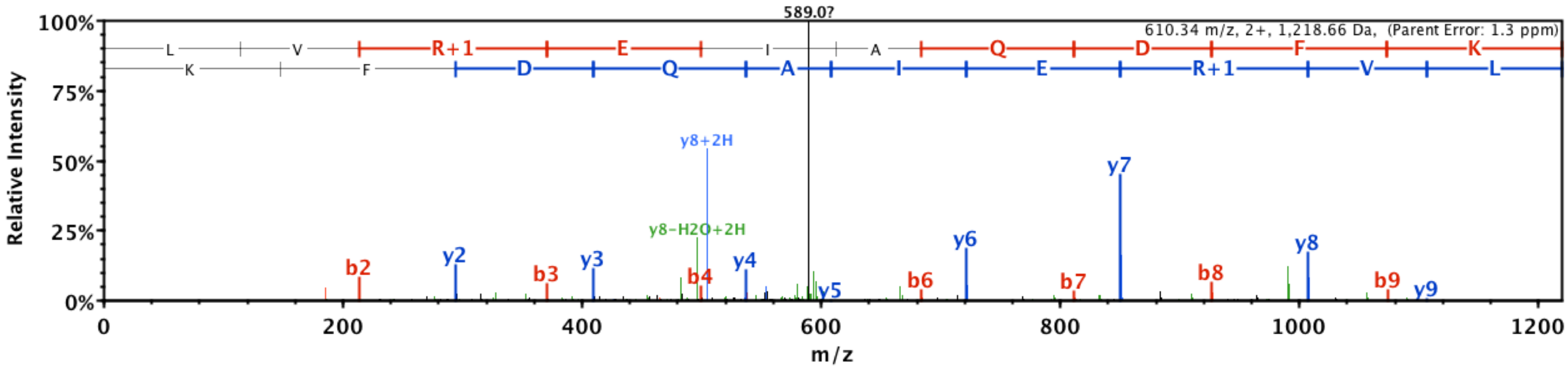

B	B Ions	B+2H	B-NH3	B-H2O	AA	Y Ions	Y+2H	Y-NH3	Y-H2O	Y
1	114.1				L	1,219.7	610.3	1,202.6	1,201.7	10
2	213.2				V	1,106.6	553.8	1,089.6	1,088.6	9
3	370.2	185.6	353.2		R+1	1,007.5	504.3	990.5	989.5	8
4	499.3	250.1	482.3	481.3	E	850.4	425.7	833.4	832.4	7
5	612.4	306.7	595.3	594.4	I	721.4	361.2	704.4	703.4	6
6	683.4	342.2	666.4	665.4	A	608.3		591.3	590.3	5
7	811.5	406.2	794.4	793.5	Q	537.3		520.2	519.3	4
8	926.5	463.8	909.5	908.5	D	409.2		392.2	391.2	3
9	1,073.6	537.3	1,056.5	1,055.6	F	294.2		277.2		2
10	1,219.7	610.3	1,202.6	1,201.7	K	147.1		130.1		1

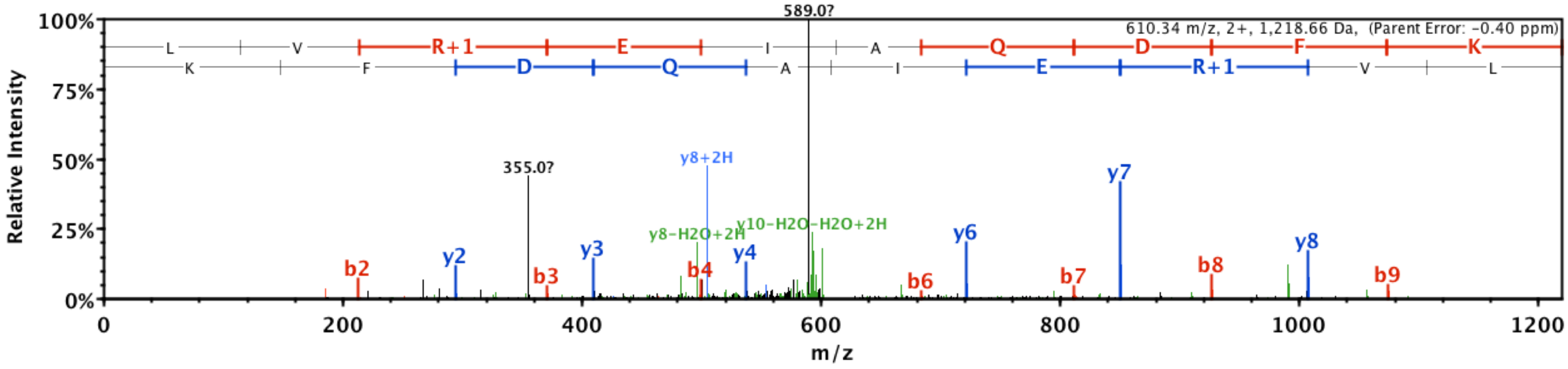

B	B Ions	B+2H	B-NH3	B-H2O	AA	Y Ions	Y+2H	Y-NH3	Y-H2O	Y
1	114.1				L	1,219.7	610.3	1,202.6	1,201.7	10
2	213.2				V	1,106.6	553.8	1,089.6	1,088.6	9
3	370.2	185.6	353.2		R+1	1,007.5	504.3	990.5	989.5	8
4	499.3	250.1	482.3	481.3	E	850.4	425.7	833.4	832.4	7
5	612.4	306.7	595.3	594.4	I	721.4	361.2	704.4	703.4	6
6	683.4	342.2	666.4	665.4	A	608.3		591.3	590.3	5
7	811.5	406.2	794.4	793.5	Q	537.3		520.2	519.3	4
8	926.5	463.8	909.5	908.5	D	409.2		392.2	391.2	3
9	1,073.6	537.3	1,056.5	1,055.6	F	294.2		277.2		2
10	1,219.7	610.3	1,202.6	1,201.7	K	147.1		130.1		1

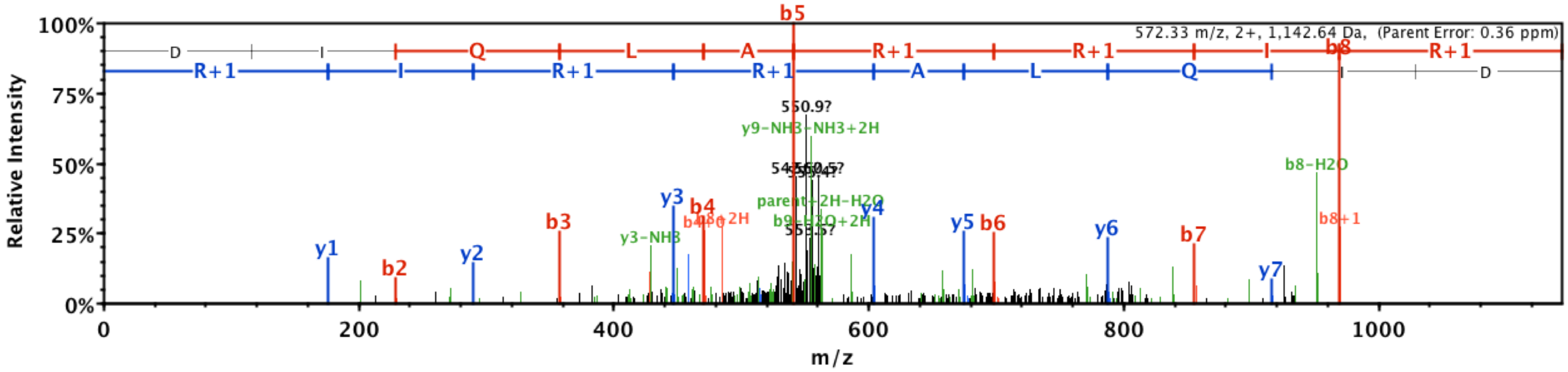

B	B Ions	B+2H	B-NH3	B-H2O	AA	Y Ions	Y+2H	Y-NH3	Y-H2O	Y
1	116.0			98.0	D	1,143.6	572.3	1,126.6	1,125.6	9
2	229.1			211.1	I	1,028.6	514.8	1,011.6		8
3	357.2		340.2	339.2	Q	915.5	458.3	898.5		7
4	470.3		453.2	452.3	L	787.5	394.2	770.5		6
5	541.3		524.3	523.3	A	674.4	337.7	657.4		5
6	698.4	349.7	681.4	680.4	R+1	603.4	302.2	586.3		4
7	855.5	428.2	838.4	837.5	R+1	446.3	223.6	429.2		3
8	968.6	484.8	951.5	950.5	I	289.2		272.2		2
9	1,143.6	572.3	1,126.6	1,125.6	R+1	176.1		159.1		1

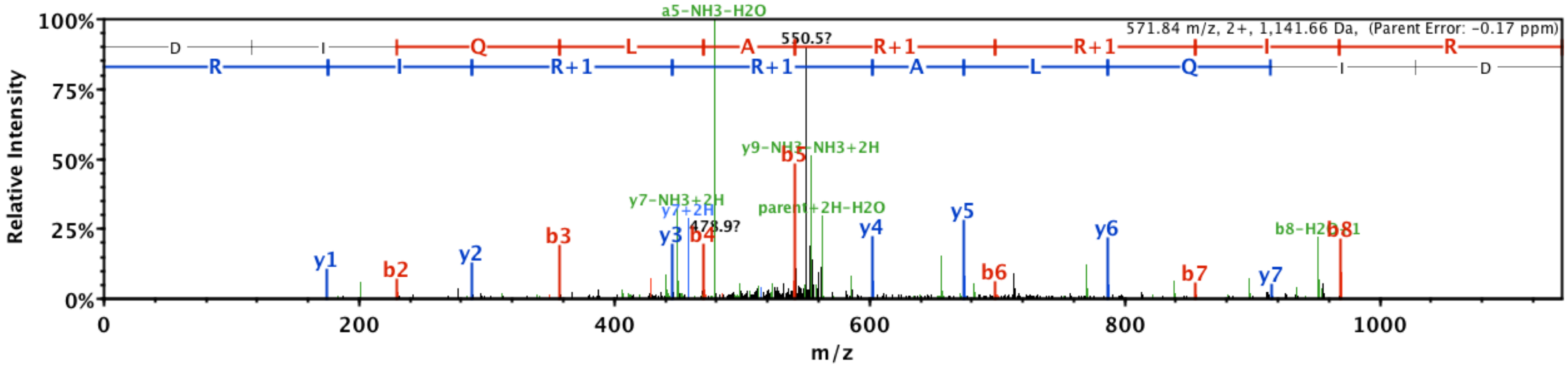

B	B Ions	B+2H	B-NH3	B-H2O	AA	Y Ions	Y+2H	Y-NH3	Y-H2O	Y
1	116.0			98.0	D	1,142.7	571.8	1,125.6	1,124.7	9
2	229.1			211.1	I	1,027.6	514.3	1,010.6		8
3	357.2		340.2	339.2	Q	914.6	457.8	897.5		7
4	470.3		453.2	452.3	L	786.5	393.8	769.5		6
5	541.3		524.3	523.3	A	673.4	337.2	656.4		5
6	698.4	349.7	681.4	680.4	R+1	602.4	301.7	585.3		4
7	855.5	428.2	838.4	837.5	R+1	445.3	223.1	428.3		3
8	968.6	484.8	951.5	950.5	I	288.2		271.2		2
9	1,142.7	571.8	1,125.6	1,124.7	R	175.1		158.1		1

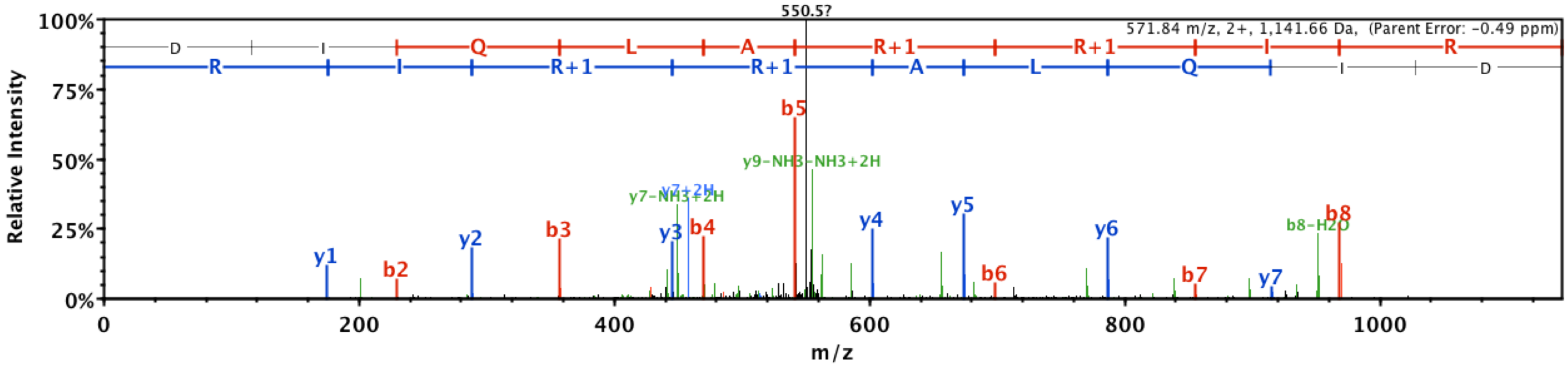

B	B Ions	B+2H	B-NH3	B-H2O	AA	Y Ions	Y+2H	Y-NH3	Y-H2O	Y
1	116.0			98.0	D	1,142.7	571.8	1,125.6	1,124.7	9
2	229.1			211.1	I	1,027.6	514.3	1,010.6		8
3	357.2		340.2	339.2	Q	914.6	457.8	897.5		7
4	470.3		453.2	452.3	L	786.5	393.8	769.5		6
5	541.3		524.3	523.3	A	673.4	337.2	656.4		5
6	698.4	349.7	681.4	680.4	R+1	602.4	301.7	585.3		4
7	855.5	428.2	838.4	837.5	R+1	445.3	223.1	428.3		3
8	968.6	484.8	951.5	950.5	I	288.2		271.2		2
9	1,142.7	571.8	1,125.6	1,124.7	R	175.1		158.1		1

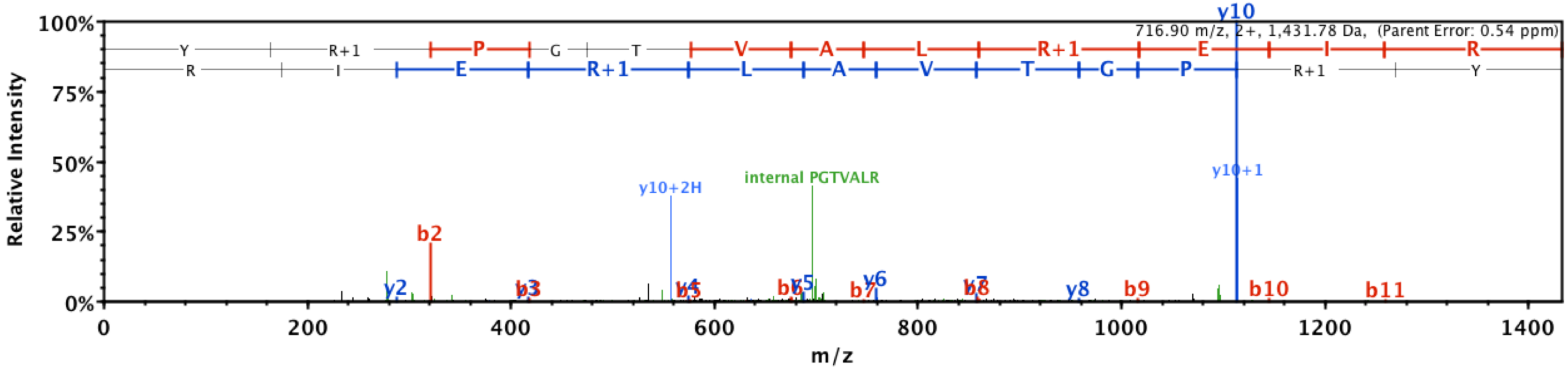

B	B Ions	B+2H	B-NH3	B-H2O	AA	Y Ions	Y+2H	Y-NH3	Y-H2O	Y
1	164.1			146.1	Y	1,432.8	716.9	1,415.8	1,414.8	12
2	321.2	161.1	304.1	303.1	R+1	1,269.7	635.4	1,252.7	1,251.7	11
3	418.2	209.6	401.2	400.2	P	1,112.6	556.8	1,095.6	1,094.6	10
4	475.2	238.1	458.2	457.2	G	1,015.6	508.3	998.6	997.6	9
5	576.3	288.6	559.3	558.3	T	958.6	479.8	941.5	940.6	8
6	675.3	338.2	658.3	657.3	V	857.5	429.3	840.5	839.5	7
7	746.4	373.7	729.4	728.4	A	758.5	379.7	741.4	740.4	6
8	859.5	430.2	842.4	841.5	L	687.4	344.2	670.4	669.4	5
9	1,016.6	508.8	999.5	998.5	R+1	574.3	287.7	557.3	556.3	4
10	1,145.6	573.3	1,128.6	1,127.6	E	417.2		400.2	399.2	3
11	1,258.7	629.8	1,241.7	1,240.7	I	288.2		271.2	270.2	2
12	1,432.8	716.9	1,415.8	1,414.8	R	175.1		158.1	157.1	1

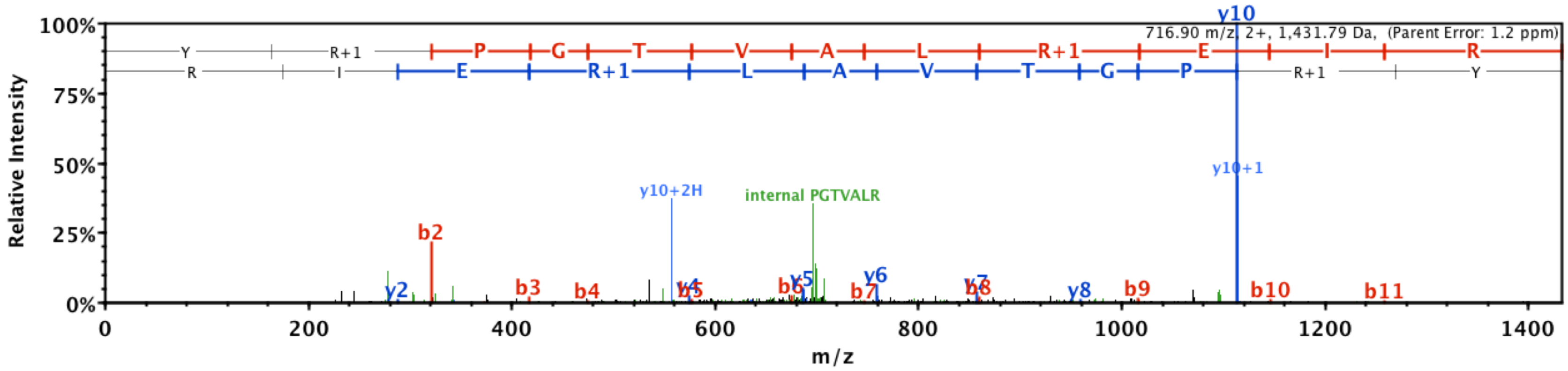

B	B Ions	B+2H	B-NH3	B-H2O	AA	Y Ions	Y+2H	Y-NH3	Y-H2O	Y
1	164.1			146.1	Y	1,432.8	716.9	1,415.8	1,414.8	12
2	321.2	161.1	304.1	303.1	R+1	1,269.7	635.4	1,252.7	1,251.7	11
3	418.2	209.6	401.2	400.2	P	1,112.6	556.8	1,095.6	1,094.6	10
4	475.2	238.1	458.2	457.2	G	1,015.6	508.3	998.6	997.6	9
5	576.3	288.6	559.3	558.3	T	958.6	479.8	941.5	940.6	8
6	675.3	338.2	658.3	657.3	V	857.5	429.3	840.5	839.5	7
7	746.4	373.7	729.4	728.4	A	758.5	379.7	741.4	740.4	6
8	859.5	430.2	842.4	841.5	L	687.4	344.2	670.4	669.4	5
9	1,016.6	508.8	999.5	998.5	R+1	574.3	287.7	557.3	556.3	4
10	1,145.6	573.3	1,128.6	1,127.6	E	417.2		400.2	399.2	3
11	1,258.7	629.8	1,241.7	1,240.7	I	288.2		271.2	270.2	2
12	1,432.8	716.9	1,415.8	1,414.8	R	175.1		158.1	157.1	1

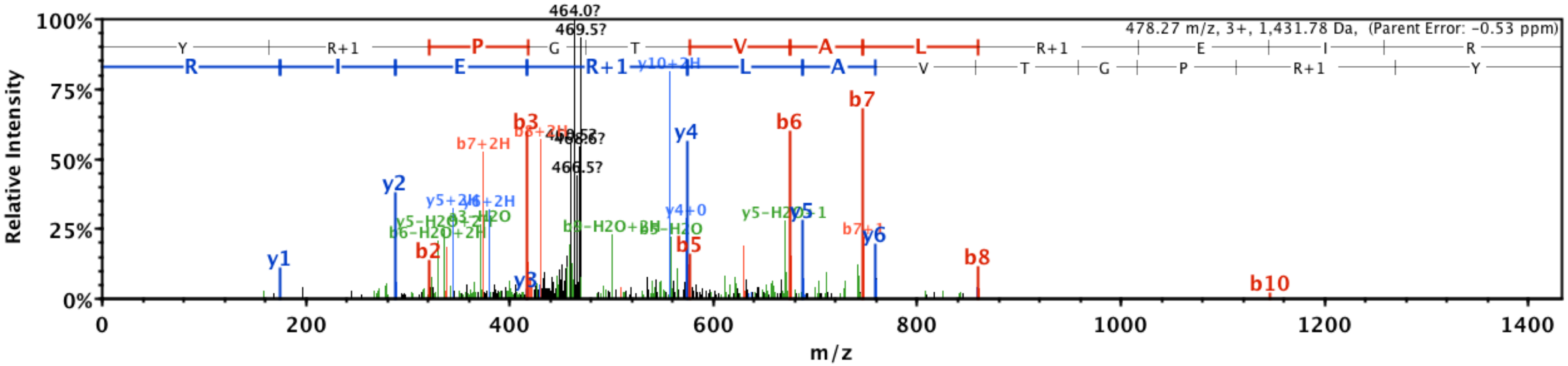

B	B Ions	B+2H	B-NH3	B-H2O	AA	Y Ions	Y+2H	Y-NH3	Y-H2O	Y
1	164.1	82.5		146.1	Y	1,432.8	716.9	1,415.8	1,414.8	12
2	321.2	161.1	304.1	303.1	R+1	1,269.7	635.4	1,252.7	1,251.7	11
3	418.2	209.6	401.2	400.2	P	1,112.6	556.8	1,095.6	1,094.6	10
4	475.2	238.1	458.2	457.2	G	1,015.6	508.3	998.6	997.6	9
5	576.3	288.6	559.3	558.3	T	958.6	479.8	941.5	940.6	8
6	675.3	338.2	658.3	657.3	V	857.5	429.3	840.5	839.5	7
7	746.4	373.7	729.4	728.4	A	758.5	379.7	741.4	740.4	6
8	859.5	430.2	842.4	841.5	L	687.4	344.2	670.4	669.4	5
9	1,016.6	508.8	999.5	998.5	R+1	574.3	287.7	557.3	556.3	4
10	1,145.6	573.3	1,128.6	1,127.6	E	417.2	209.1	400.2	399.2	3
11	1,258.7	629.8	1,241.7	1,240.7	I	288.2	144.6	271.2	270.2	2
12	1,432.8	716.9	1,415.8	1,414.8	R	175.1	88.1	158.1	157.1	1

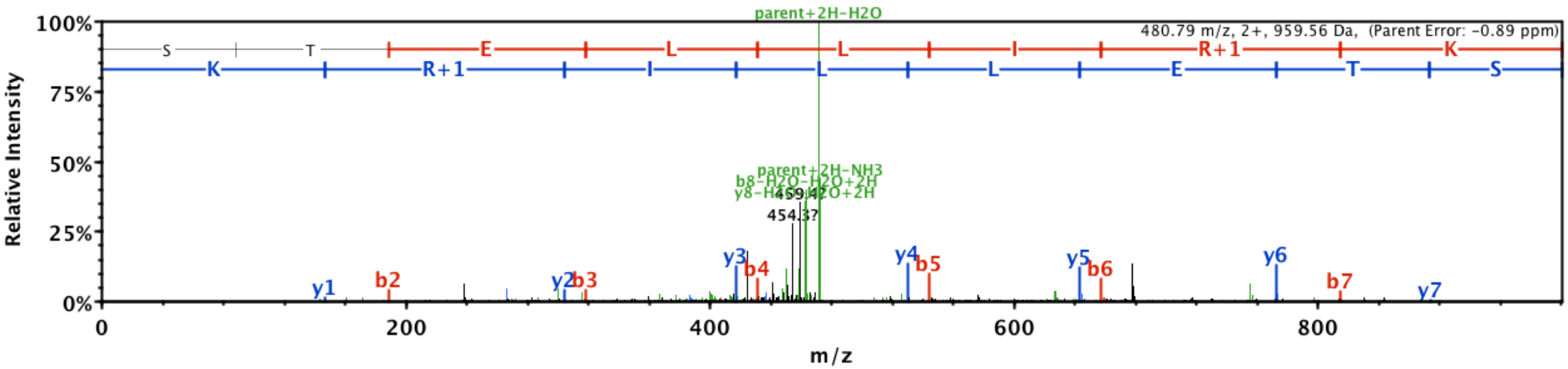

B	B Ions	B+2H	B-NH3	B-H2O	AA	Y Ions	Y+2H	Y-NH3	Y-H2O	Y
1	88.0			70.0	S	960.6	480.8	943.5	942.6	8
2	189.1			171.1	T	873.5	437.3	856.5	855.5	7
3	318.1			300.1	E	772.5	386.8	755.5	754.5	6
4	431.2			413.2	L	643.5	322.2	626.4		5
5	544.3			526.3	L	530.4	265.7	513.3		4
6	657.4	329.2		639.4	I	417.3	209.1	400.3		3
7	814.5	407.7	797.4	796.5	R+1	304.2	152.6	287.2		2
8	960.6	480.8	943.5	942.6	K	147.1		130.1		1

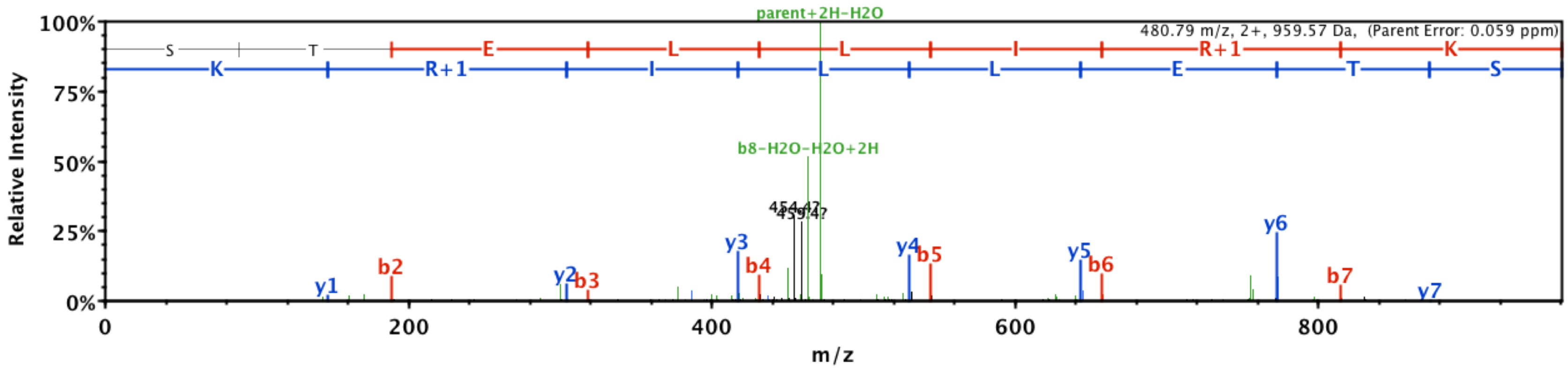

B	B Ions	B+2H	B-NH3	B-H2O	AA	Y Ions	Y+2H	Y-NH3	Y-H2O	Y
1	88.0			70.0	S	960.6	480.8	943.5	942.6	8
2	189.1			171.1	T	873.5	437.3	856.5	855.5	7
3	318.1			300.1	E	772.5	386.8	755.5	754.5	6
4	431.2			413.2	L	643.5	322.2	626.4		5
5	544.3			526.3	L	530.4	265.7	513.3		4
6	657.4	329.2		639.4	I	417.3	209.1	400.3		3
7	814.5	407.7	797.4	796.5	R+1	304.2	152.6	287.2		2
8	960.6	480.8	943.5	942.6	K	147.1		130.1		1

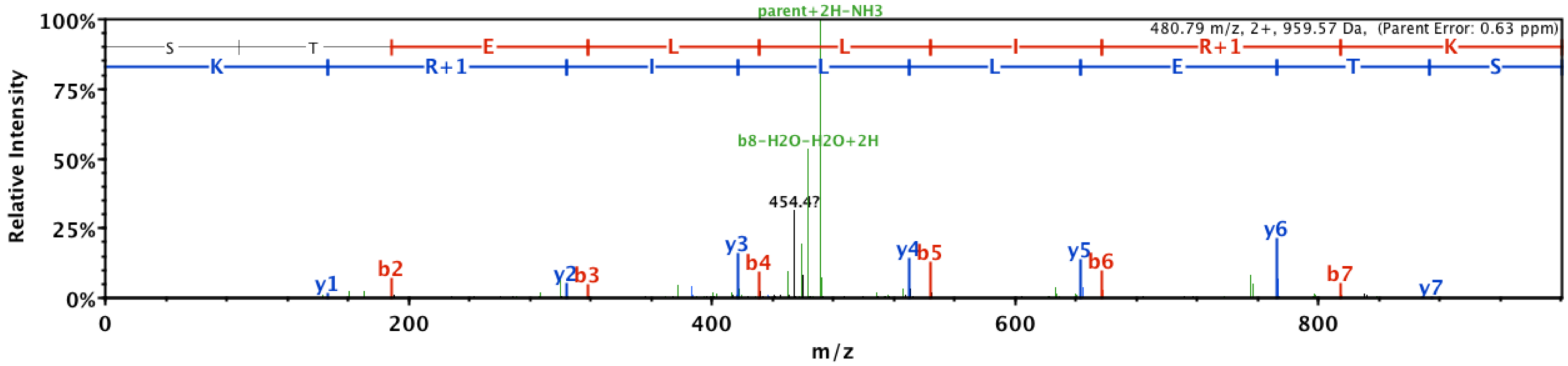

B	B Ions	B+2H	B-NH3	B-H2O	AA	Y Ions	Y+2H	Y-NH3	Y-H2O	Y
1	88.0			70.0	S	960.6	480.8	943.5	942.6	8
2	189.1			171.1	T	873.5	437.3	856.5	855.5	7
3	318.1			300.1	E	772.5	386.8	755.5	754.5	6
4	431.2			413.2	L	643.5	322.2	626.4		5
5	544.3			526.3	L	530.4	265.7	513.3		4
6	657.4	329.2		639.4	I	417.3	209.1	400.3		3
7	814.5	407.7	797.4	796.5	R+1	304.2	152.6	287.2		2
8	960.6	480.8	943.5	942.6	K	147.1		130.1		1

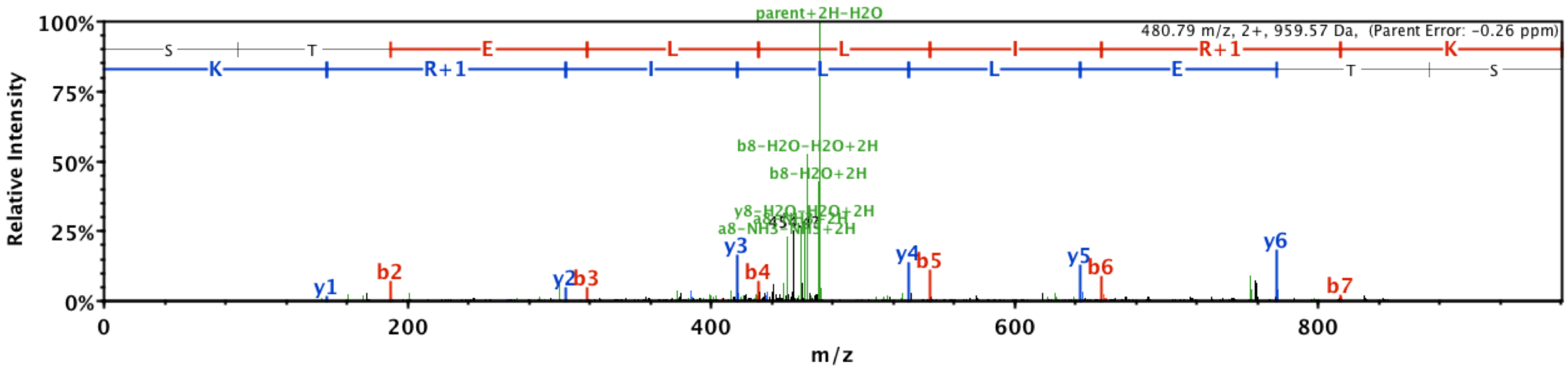

B	B Ions	B+2H	B-NH3	B-H2O	AA	Y Ions	Y+2H	Y-NH3	Y-H2O	Y
1	88.0			70.0	S	960.6	480.8	943.5	942.6	8
2	189.1			171.1	T	873.5	437.3	856.5	855.5	7
3	318.1			300.1	E	772.5	386.8	755.5	754.5	6
4	431.2			413.2	L	643.5	322.2	626.4		5
5	544.3			526.3	L	530.4	265.7	513.3		4
6	657.4	329.2		639.4	I	417.3	209.1	400.3		3
7	814.5	407.7	797.4	796.5	R+1	304.2	152.6	287.2		2
8	960.6	480.8	943.5	942.6	K	147.1		130.1		1

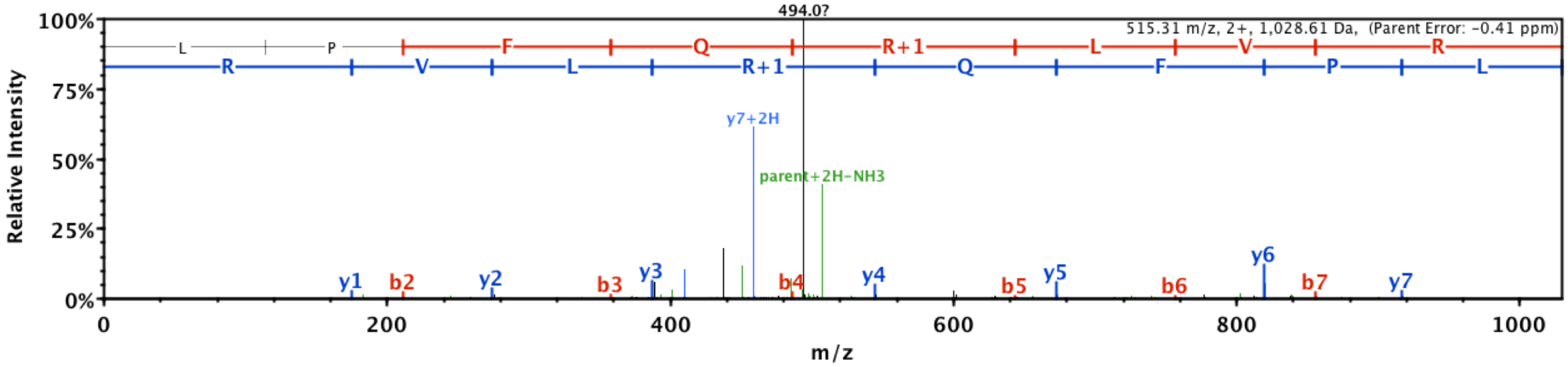

B	B Ions	B+2H	B-NH3	B-H2O	AA	Y Ions	Y+2H	Y-NH3	Y-H2O	Y
1	114.1				L	1,029.6	515.3	1,012.6		8
2	211.1				P	916.5	458.8	899.5		7
3	358.2				F	819.5	410.2	802.5		6
4	486.3		469.2		Q	672.4	336.7	655.4		5
5	643.4	322.2	626.3		R+1	544.4	272.7	527.3		4
6	756.4	378.7	739.4		L	387.3		370.2		3
7	855.5	428.3	838.5		V	274.2		257.2		2
8	1,029.6	515.3	1,012.6		R	175.1		158.1		1

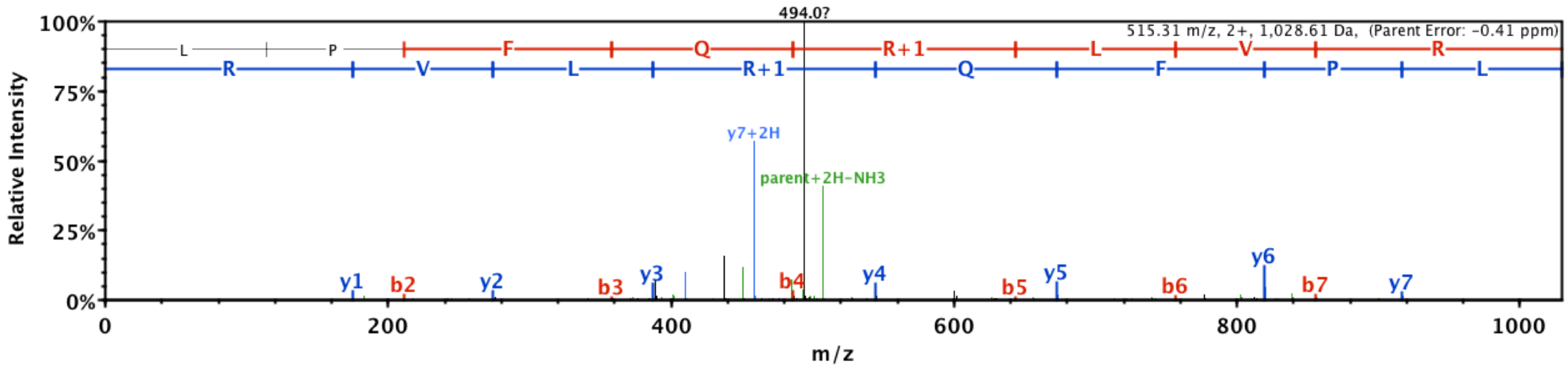

B	B Ions	B+2H	B-NH3	B-H2O	AA	Y Ions	Y+2H	Y-NH3	Y-H2O	Y
1	114.1				L	1,029.6	515.3	1,012.6		8
2	211.1				P	916.5	458.8	899.5		7
3	358.2				F	819.5	410.2	802.5		6
4	486.3		469.2		Q	672.4	336.7	655.4		5
5	643.4	322.2	626.3		R+1	544.4	272.7	527.3		4
6	756.4	378.7	739.4		L	387.3		370.2		3
7	855.5	428.3	838.5		V	274.2		257.2		2
8	1,029.6	515.3	1,012.6		R	175.1		158.1		1

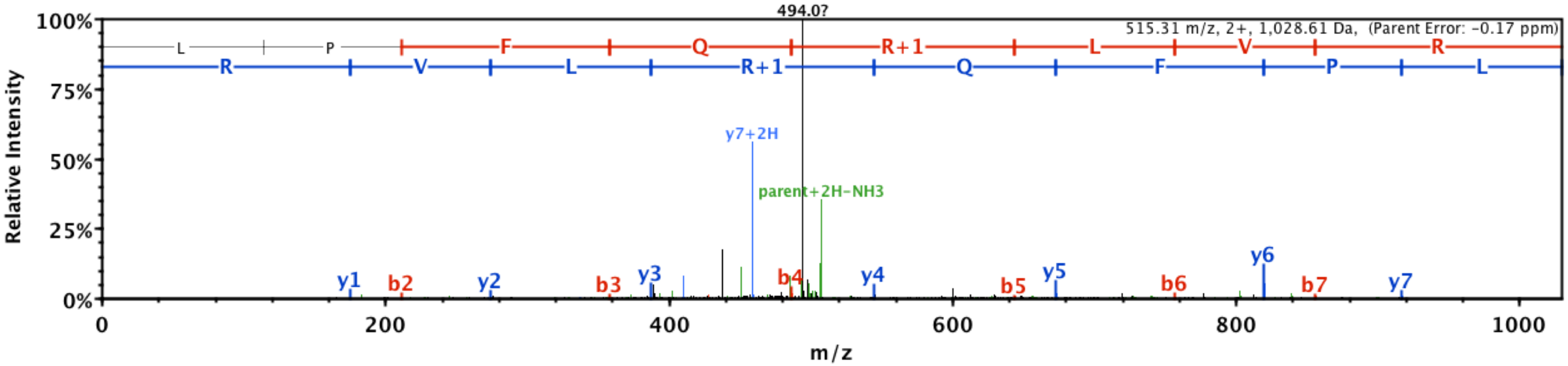

B	B Ions	B+2H	B-NH3	B-H2O	AA	Y Ions	Y+2H	Y-NH3	Y-H2O	Y
1	114.1				L	1,029.6	515.3	1,012.6		8
2	211.1				P	916.5	458.8	899.5		7
3	358.2				F	819.5	410.2	802.5		6
4	486.3		469.2		Q	672.4	336.7	655.4		5
5	643.4	322.2	626.3		R+1	544.4	272.7	527.3		4
6	756.4	378.7	739.4		L	387.3		370.2		3
7	855.5	428.3	838.5		V	274.2		257.2		2
8	1,029.6	515.3	1,012.6		R	175.1		158.1		1

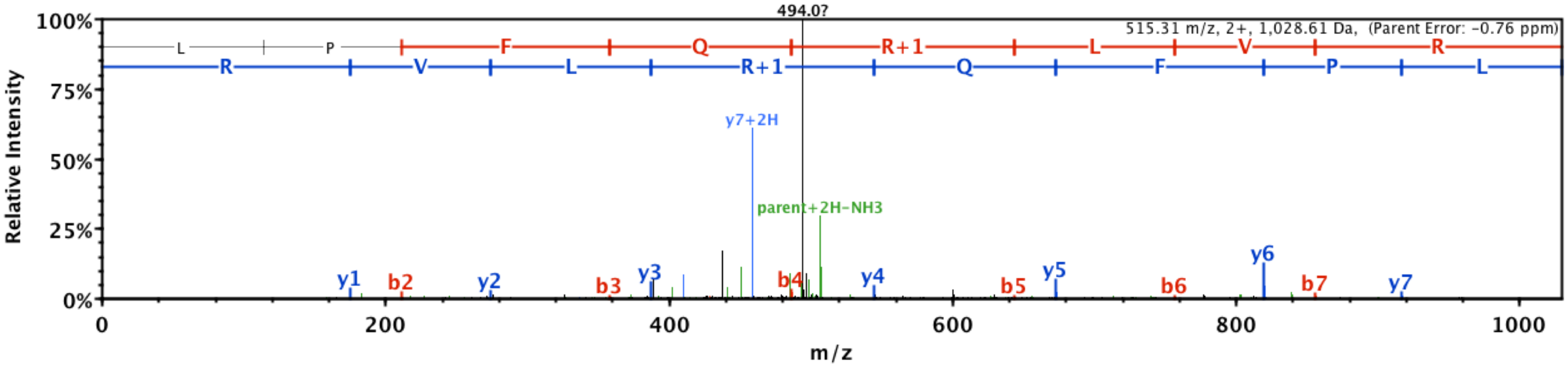

B	B Ions	B+2H	B-NH3	B-H2O	AA	Y Ions	Y+2H	Y-NH3	Y-H2O	Y
1	114.1				L	1,029.6	515.3	1,012.6		8
2	211.1				P	916.5	458.8	899.5		7
3	358.2				F	819.5	410.2	802.5		6
4	486.3		469.2		Q	672.4	336.7	655.4		5
5	643.4	322.2	626.3		R+1	544.4	272.7	527.3		4
6	756.4	378.7	739.4		L	387.3		370.2		3
7	855.5	428.3	838.5		V	274.2		257.2		2
8	1,029.6	515.3	1,012.6		R	175.1		158.1		1

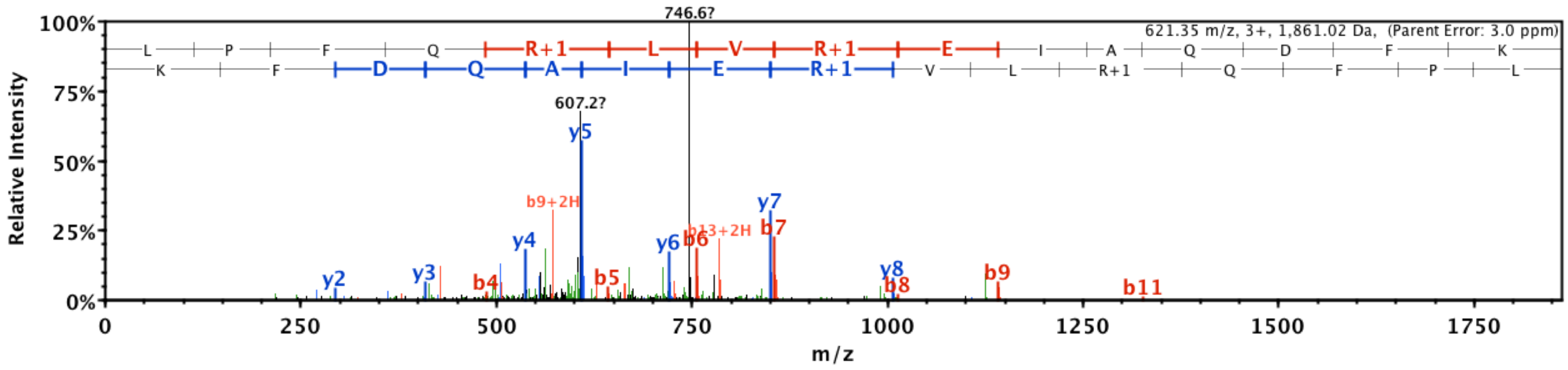

B	B Ions	B+2H	B-NH3	B-H2O	AA	Y Ions	Y+2H	Y-NH3	Y-H2O	Y
1	114.1	57.5			L	1,862.0	931.5	1,845.0	1,844.0	15
2	211.1	106.1			P	1,748.9	875.0	1,731.9	1,730.9	14
3	358.2	179.6			F	1,651.9	826.4	1,634.9	1,633.9	13
4	486.3	243.6	469.2		Q	1,504.8	752.9	1,487.8	1,486.8	12
5	643.4	322.2	626.3		R+1	1,376.8	688.9	1,359.7	1,358.7	11
6	756.4	378.7	739.4		L	1,219.7	610.3	1,202.6	1,201.7	10
7	855.5	428.3	838.5		V	1,106.6	553.8	1,089.6	1,088.6	9
8	1,012.6	506.8	995.6		R+1	1,007.5	504.3	990.5	989.5	8
9	1,141.6	571.3	1,124.6	1,123.6	E	850.4	425.7	833.4	832.4	7
10	1,254.7	627.9	1,237.7	1,236.7	I	721.4	361.2	704.4	703.4	6
11	1,325.8	663.4	1,308.7	1,307.7	A	608.3	304.7	591.3	590.3	5
12	1,453.8	727.4	1,436.8	1,435.8	Q	537.3	269.1	520.2	519.3	4
13	1,568.8	784.9	1,551.8	1,550.8	D	409.2	205.1	392.2	391.2	3
14	1,715.9	858.5	1,698.9	1,697.9	F	294.2	147.6	277.2		2
15	1,862.0	931.5	1,845.0	1,844.0	K	147.1	74.1	130.1		1

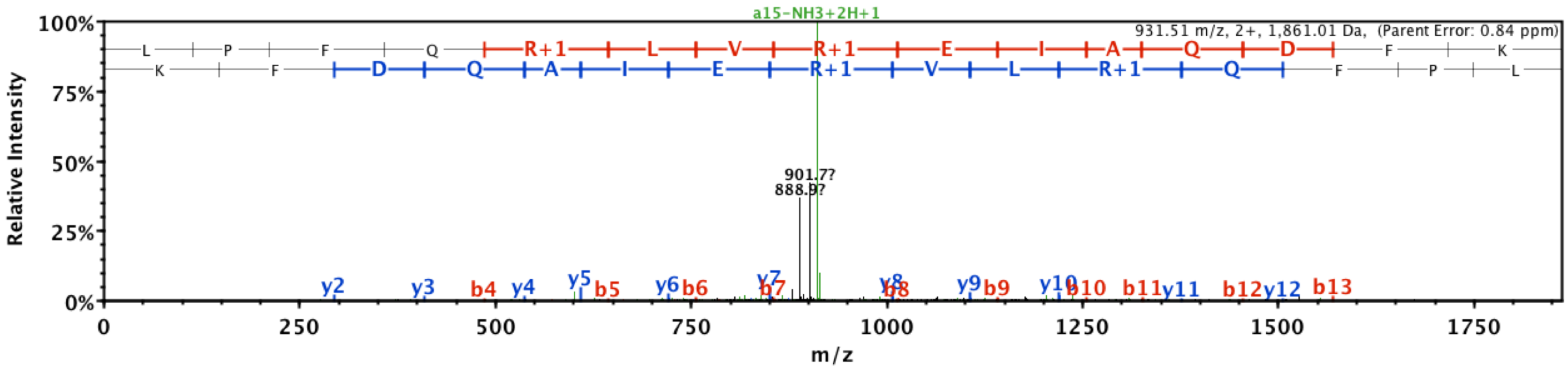

B	B Ions	B+2H	B-NH3	B-H2O	AA	Y Ions	Y+2H	Y-NH3	Y-H2O	Y
1	114.1				L	1,862.0	931.5	1,845.0	1,844.0	15
2	211.1				P	1,748.9	875.0	1,731.9	1,730.9	14
3	358.2				F	1,651.9	826.4	1,634.9	1,633.9	13
4	486.3		469.2		Q	1,504.8	752.9	1,487.8	1,486.8	12
5	643.4	322.2	626.3		R+1	1,376.8	688.9	1,359.7	1,358.7	11
6	756.4	378.7	739.4		L	1,219.7	610.3	1,202.6	1,201.7	10
7	855.5	428.3	838.5		V	1,106.6	553.8	1,089.6	1,088.6	9
8	1,012.6	506.8	995.6		R+1	1,007.5	504.3	990.5	989.5	8
9	1,141.6	571.3	1,124.6	1,123.6	E	850.4	425.7	833.4	832.4	7
10	1,254.7	627.9	1,237.7	1,236.7	I	721.4	361.2	704.4	703.4	6
11	1,325.8	663.4	1,308.7	1,307.7	A	608.3		591.3	590.3	5
12	1,453.8	727.4	1,436.8	1,435.8	Q	537.3		520.2	519.3	4
13	1,568.8	784.9	1,551.8	1,550.8	D	409.2		392.2	391.2	3
14	1,715.9	858.5	1,698.9	1,697.9	F	294.2		277.2		2
15	1,862.0	931.5	1,845.0	1,844.0	K	147.1		130.1		1

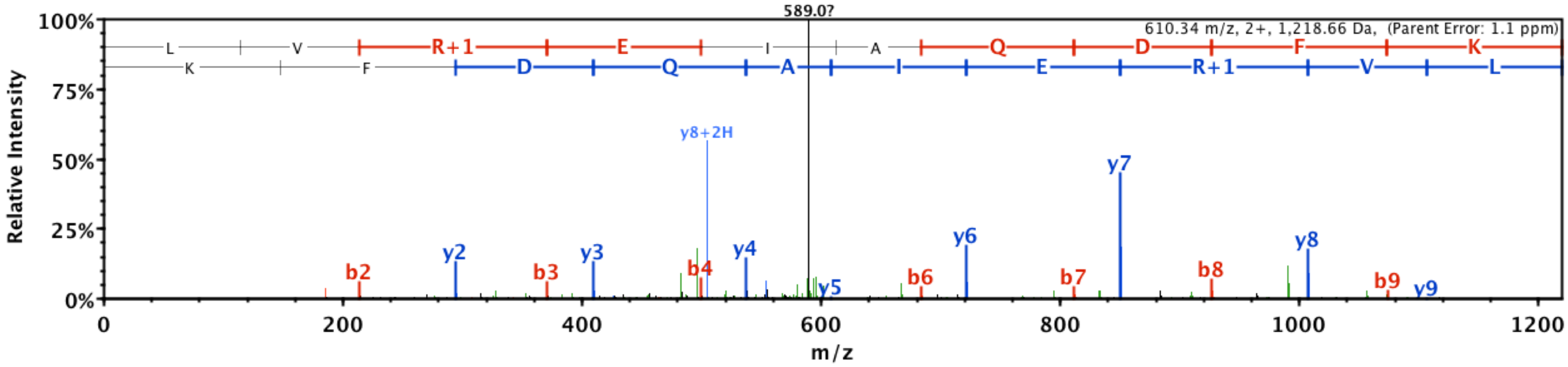

B	B Ions	B+2H	B-NH3	B-H2O	AA	Y Ions	Y+2H	Y-NH3	Y-H2O	Y
1	114.1				L	1,219.7	610.3	1,202.6	1,201.7	10
2	213.2				V	1,106.6	553.8	1,089.6	1,088.6	9
3	370.2	185.6	353.2		R+1	1,007.5	504.3	990.5	989.5	8
4	499.3	250.1	482.3	481.3	E	850.4	425.7	833.4	832.4	7
5	612.4	306.7	595.3	594.4	I	721.4	361.2	704.4	703.4	6
6	683.4	342.2	666.4	665.4	A	608.3		591.3	590.3	5
7	811.5	406.2	794.4	793.5	Q	537.3		520.2	519.3	4
8	926.5	463.8	909.5	908.5	D	409.2		392.2	391.2	3
9	1,073.6	537.3	1,056.5	1,055.6	F	294.2		277.2		2
10	1,219.7	610.3	1,202.6	1,201.7	K	147.1		130.1		1

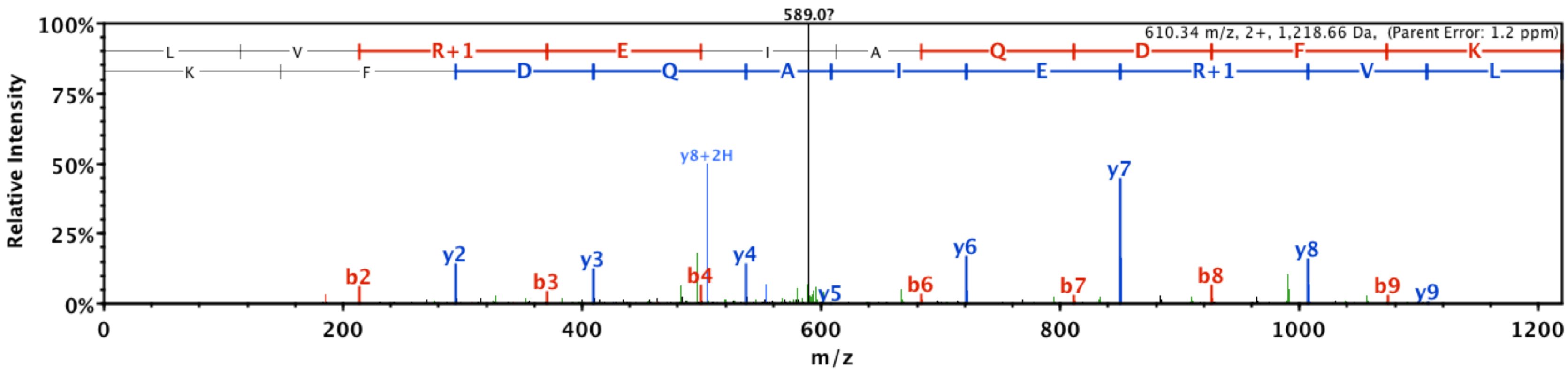

B	B Ions	B+2H	B-NH3	B-H2O	AA	Y Ions	Y+2H	Y-NH3	Y-H2O	Y
1	114.1				L	1,219.7	610.3	1,202.6	1,201.7	10
2	213.2				V	1,106.6	553.8	1,089.6	1,088.6	9
3	370.2	185.6	353.2		R+1	1,007.5	504.3	990.5	989.5	8
4	499.3	250.1	482.3	481.3	E	850.4	425.7	833.4	832.4	7
5	612.4	306.7	595.3	594.4	I	721.4	361.2	704.4	703.4	6
6	683.4	342.2	666.4	665.4	A	608.3		591.3	590.3	5
7	811.5	406.2	794.4	793.5	Q	537.3		520.2	519.3	4
8	926.5	463.8	909.5	908.5	D	409.2		392.2	391.2	3
9	1,073.6	537.3	1,056.5	1,055.6	F	294.2		277.2		2
10	1,219.7	610.3	1,202.6	1,201.7	K	147.1		130.1		1

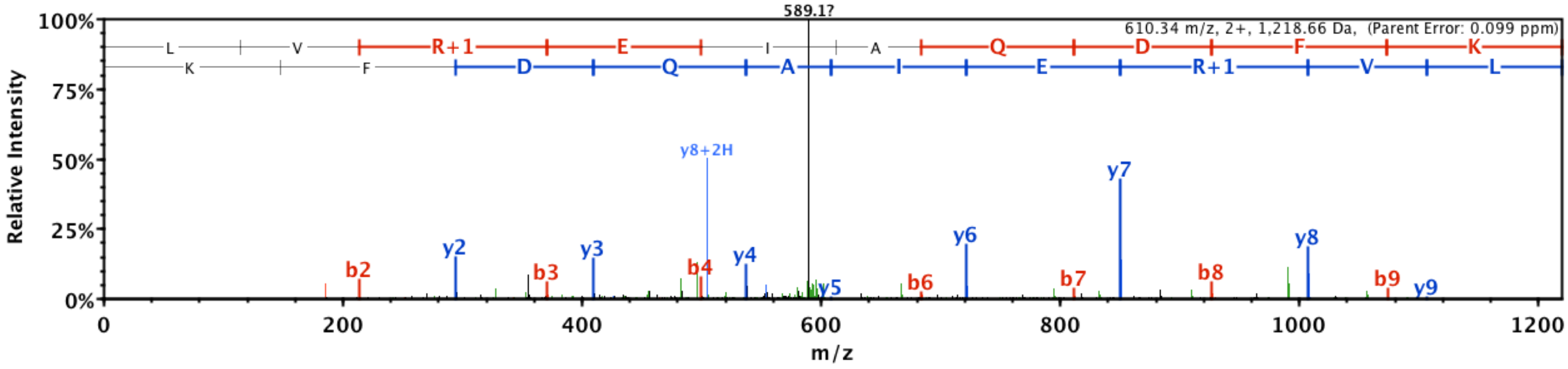

B	B Ions	B+2H	B-NH3	B-H2O	AA	Y Ions	Y+2H	Y-NH3	Y-H2O	Y
1	114.1				L	1,219.7	610.3	1,202.6	1,201.7	10
2	213.2				V	1,106.6	553.8	1,089.6	1,088.6	9
3	370.2	185.6	353.2		R+1	1,007.5	504.3	990.5	989.5	8
4	499.3	250.1	482.3	481.3	E	850.4	425.7	833.4	832.4	7
5	612.4	306.7	595.3	594.4	I	721.4	361.2	704.4	703.4	6
6	683.4	342.2	666.4	665.4	A	608.3		591.3	590.3	5
7	811.5	406.2	794.4	793.5	Q	537.3		520.2	519.3	4
8	926.5	463.8	909.5	908.5	D	409.2		392.2	391.2	3
9	1,073.6	537.3	1,056.5	1,055.6	F	294.2		277.2		2
10	1,219.7	610.3	1,202.6	1,201.7	K	147.1		130.1		1

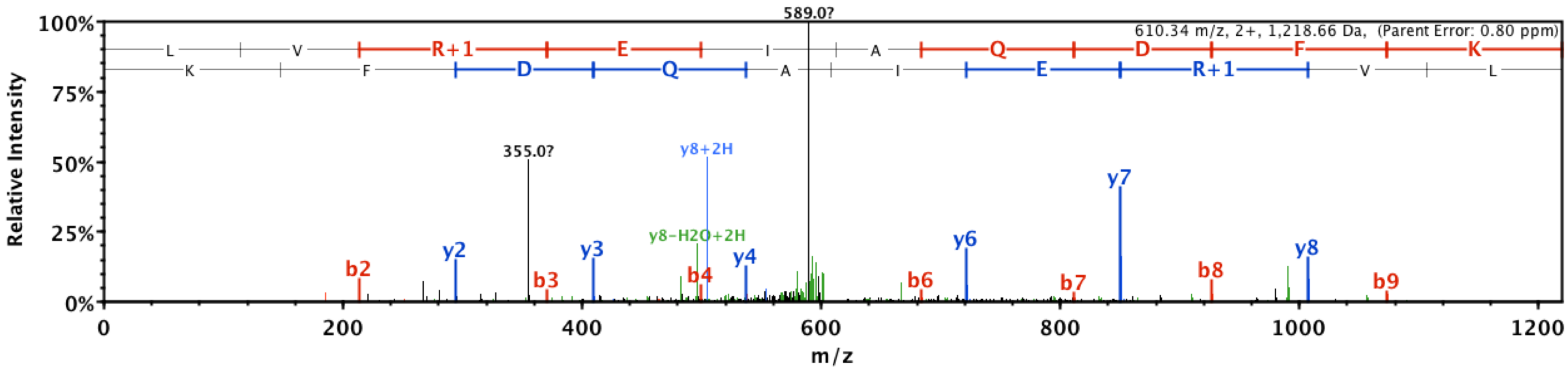

B	B Ions	B+2H	B-NH3	B-H2O	AA	Y Ions	Y+2H	Y-NH3	Y-H2O	Y
1	114.1				L	1,219.7	610.3	1,202.6	1,201.7	10
2	213.2				V	1,106.6	553.8	1,089.6	1,088.6	9
3	370.2	185.6	353.2		R+1	1,007.5	504.3	990.5	989.5	8
4	499.3	250.1	482.3	481.3	E	850.4	425.7	833.4	832.4	7
5	612.4	306.7	595.3	594.4	I	721.4	361.2	704.4	703.4	6
6	683.4	342.2	666.4	665.4	A	608.3		591.3	590.3	5
7	811.5	406.2	794.4	793.5	Q	537.3		520.2	519.3	4
8	926.5	463.8	909.5	908.5	D	409.2		392.2	391.2	3
9	1,073.6	537.3	1,056.5	1,055.6	F	294.2		277.2		2
10	1,219.7	610.3	1,202.6	1,201.7	K	147.1		130.1		1

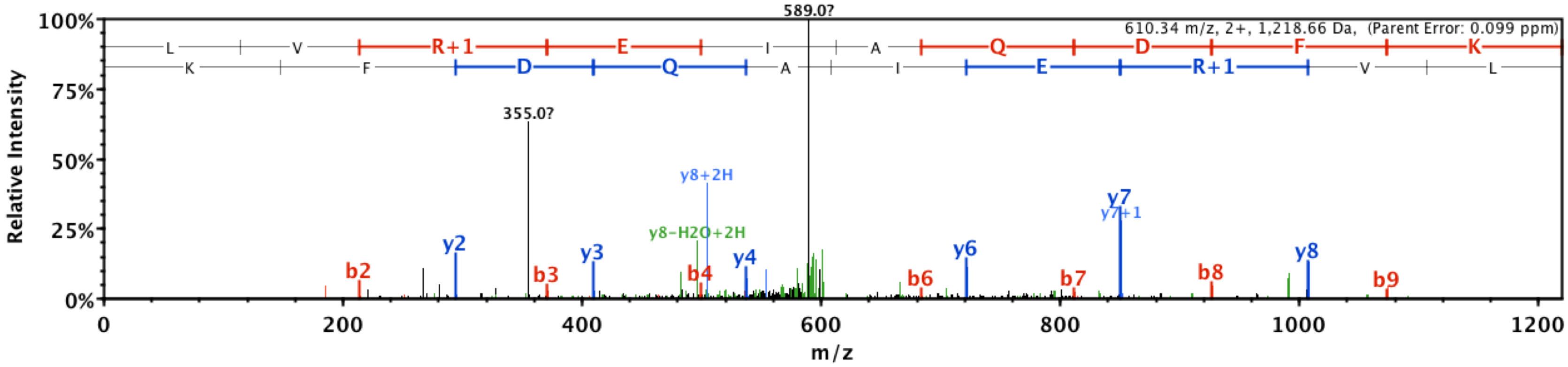

B	B Ions	B+2H	B-NH3	B-H2O	AA	Y Ions	Y+2H	Y-NH3	Y-H2O	Y
1	114.1				L	1,219.7	610.3	1,202.6	1,201.7	10
2	213.2				V	1,106.6	553.8	1,089.6	1,088.6	9
3	370.2	185.6	353.2		R+1	1,007.5	504.3	990.5	989.5	8
4	499.3	250.1	482.3	481.3	E	850.4	425.7	833.4	832.4	7
5	612.4	306.7	595.3	594.4	I	721.4	361.2	704.4	703.4	6
6	683.4	342.2	666.4	665.4	A	608.3		591.3	590.3	5
7	811.5	406.2	794.4	793.5	Q	537.3		520.2	519.3	4
8	926.5	463.8	909.5	908.5	D	409.2		392.2	391.2	3
9	1,073.6	537.3	1,056.5	1,055.6	F	294.2		277.2		2
10	1,219.7	610.3	1,202.6	1,201.7	K	147.1		130.1		1

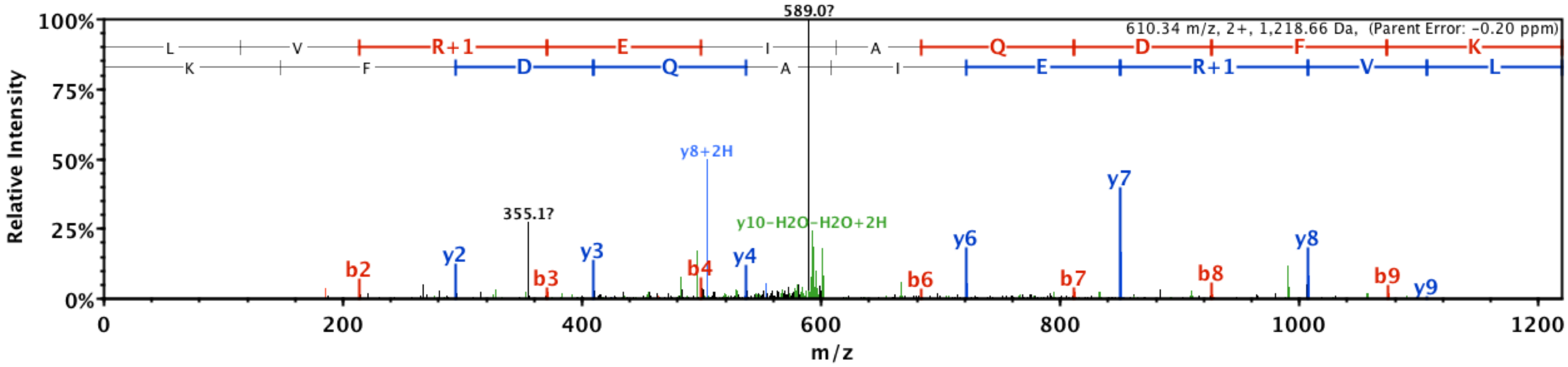

B	B Ions	B+2H	B-NH3	B-H2O	AA	Y Ions	Y+2H	Y-NH3	Y-H2O	Y
1	114.1				L	1,219.7	610.3	1,202.6	1,201.7	10
2	213.2				V	1,106.6	553.8	1,089.6	1,088.6	9
3	370.2	185.6	353.2		R+1	1,007.5	504.3	990.5	989.5	8
4	499.3	250.1	482.3	481.3	E	850.4	425.7	833.4	832.4	7
5	612.4	306.7	595.3	594.4	I	721.4	361.2	704.4	703.4	6
6	683.4	342.2	666.4	665.4	A	608.3		591.3	590.3	5
7	811.5	406.2	794.4	793.5	Q	537.3		520.2	519.3	4
8	926.5	463.8	909.5	908.5	D	409.2		392.2	391.2	3
9	1,073.6	537.3	1,056.5	1,055.6	F	294.2		277.2		2
10	1,219.7	610.3	1,202.6	1,201.7	K	147.1		130.1		1

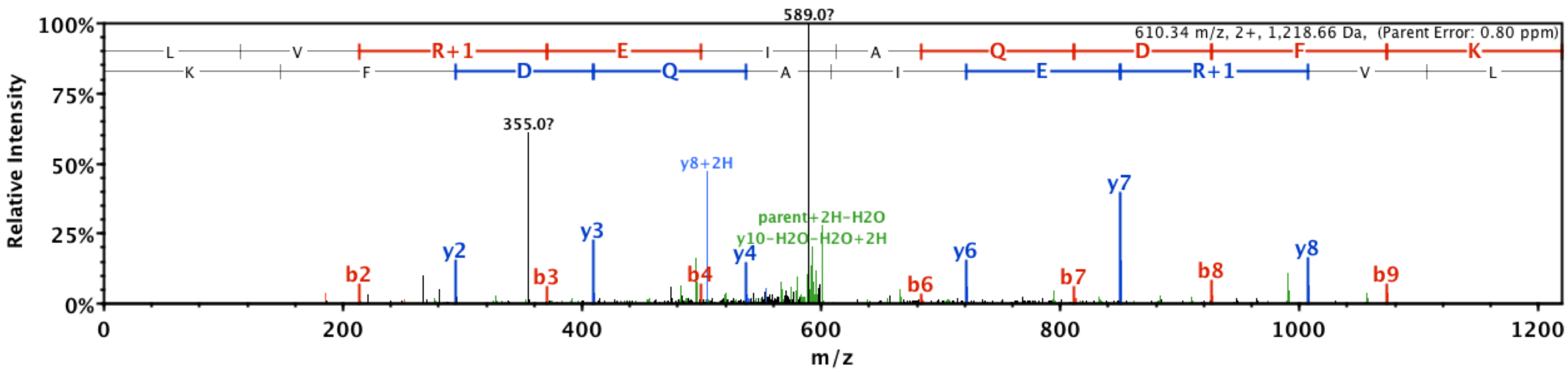

B	B Ions	B+2H	B-NH3	B-H2O	AA	Y Ions	Y+2H	Y-NH3	Y-H2O	Y
1	114.1				L	1,219.7	610.3	1,202.6	1,201.7	10
2	213.2				V	1,106.6	553.8	1,089.6	1,088.6	9
3	370.2	185.6	353.2		R+1	1,007.5	504.3	990.5	989.5	8
4	499.3	250.1	482.3	481.3	E	850.4	425.7	833.4	832.4	7
5	612.4	306.7	595.3	594.4	I	721.4	361.2	704.4	703.4	6
6	683.4	342.2	666.4	665.4	A	608.3		591.3	590.3	5
7	811.5	406.2	794.4	793.5	Q	537.3		520.2	519.3	4
8	926.5	463.8	909.5	908.5	D	409.2		392.2	391.2	3
9	1,073.6	537.3	1,056.5	1,055.6	F	294.2		277.2		2
10	1,219.7	610.3	1,202.6	1,201.7	K	147.1		130.1		1

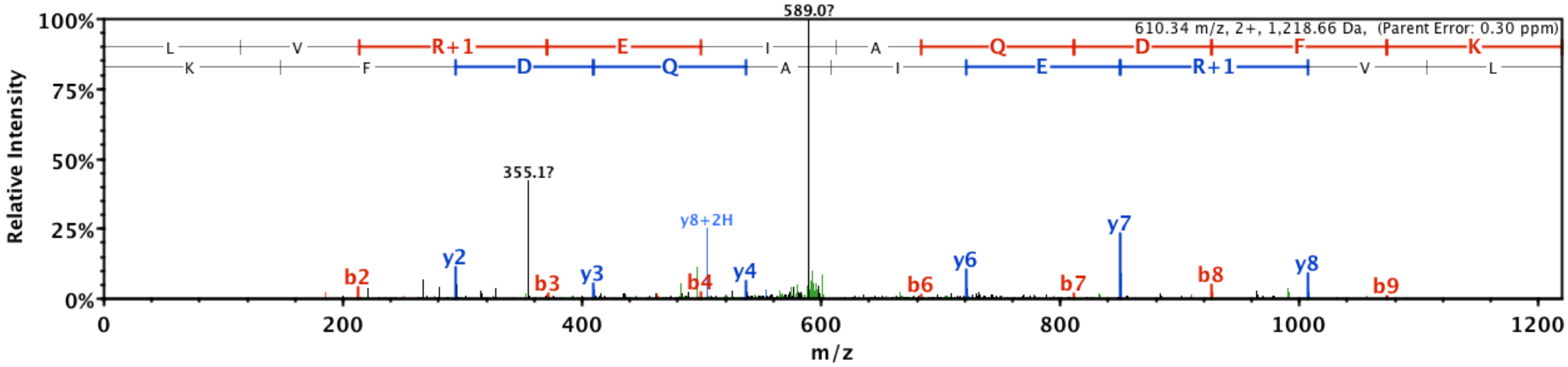

B	B Ions	B+2H	B-NH3	B-H2O	AA	Y Ions	Y+2H	Y-NH3	Y-H2O	Y
1	114.1				L	1,219.7	610.3	1,202.6	1,201.7	10
2	213.2				V	1,106.6	553.8	1,089.6	1,088.6	9
3	370.2	185.6	353.2		R+1	1,007.5	504.3	990.5	989.5	8
4	499.3	250.1	482.3	481.3	E	850.4	425.7	833.4	832.4	7
5	612.4	306.7	595.3	594.4	I	721.4	361.2	704.4	703.4	6
6	683.4	342.2	666.4	665.4	A	608.3		591.3	590.3	5
7	811.5	406.2	794.4	793.5	Q	537.3		520.2	519.3	4
8	926.5	463.8	909.5	908.5	D	409.2		392.2	391.2	3
9	1,073.6	537.3	1,056.5	1,055.6	F	294.2		277.2		2
10	1,219.7	610.3	1,202.6	1,201.7	K	147.1		130.1		1

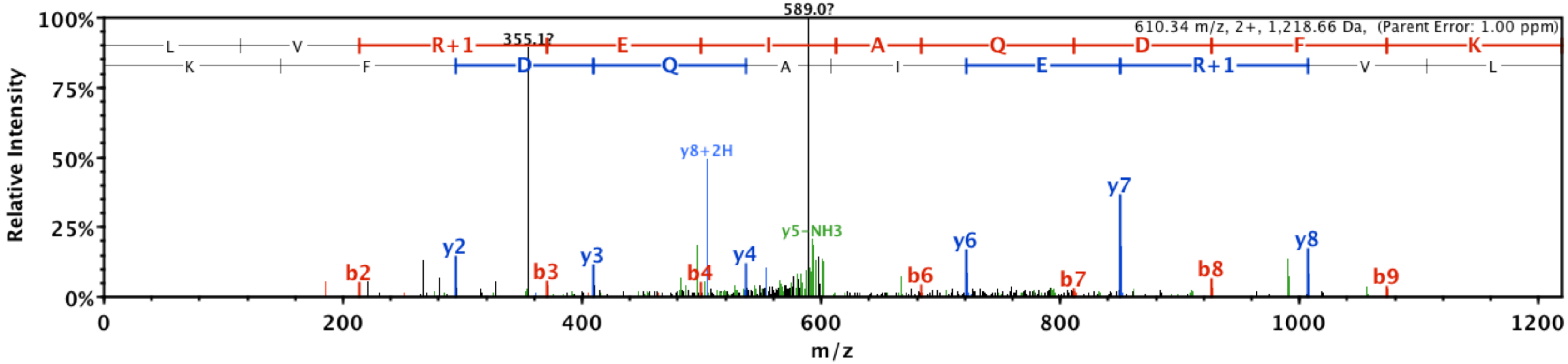

B	B Ions	B+2H	B-NH3	B-H2O	AA	Y Ions	Y+2H	Y-NH3	Y-H2O	Y
1	114.1				L	1,219.7	610.3	1,202.6	1,201.7	10
2	213.2				V	1,106.6	553.8	1,089.6	1,088.6	9
3	370.2	185.6	353.2		R+1	1,007.5	504.3	990.5	989.5	8
4	499.3	250.1	482.3	481.3	E	850.4	425.7	833.4	832.4	7
5	612.4	306.7	595.3	594.4	I	721.4	361.2	704.4	703.4	6
6	683.4	342.2	666.4	665.4	A	608.3		591.3	590.3	5
7	811.5	406.2	794.4	793.5	Q	537.3		520.2	519.3	4
8	926.5	463.8	909.5	908.5	D	409.2		392.2	391.2	3
9	1,073.6	537.3	1,056.5	1,055.6	F	294.2		277.2		2
10	1,219.7	610.3	1,202.6	1,201.7	K	147.1		130.1		1

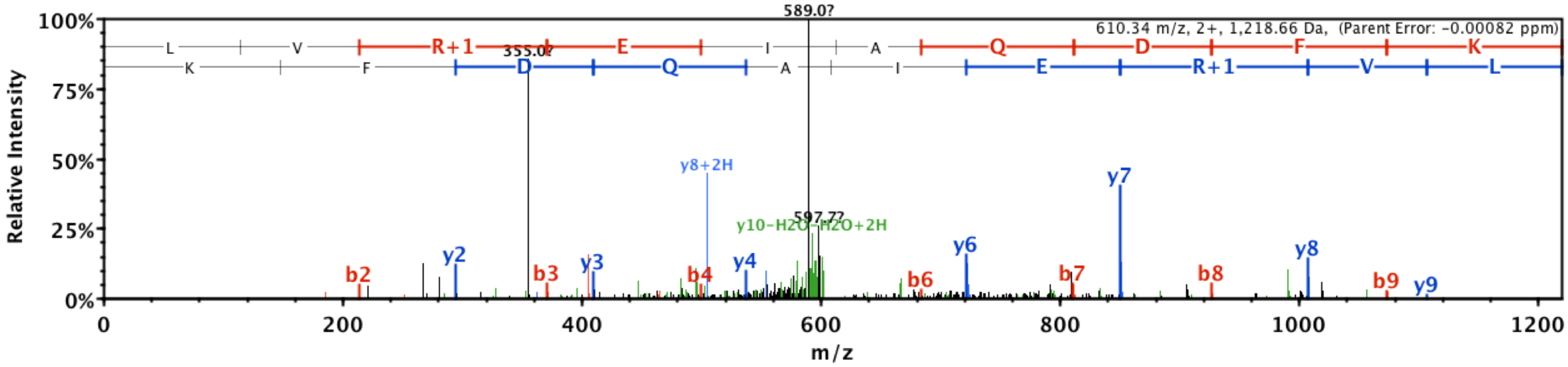

B	B Ions	B+2H	B-NH3	B-H2O	AA	Y Ions	Y+2H	Y-NH3	Y-H2O	Y
1	114.1				L	1,219.7	610.3	1,202.6	1,201.7	10
2	213.2				V	1,106.6	553.8	1,089.6	1,088.6	9
3	370.2	185.6	353.2		R+1	1,007.5	504.3	990.5	989.5	8
4	499.3	250.1	482.3	481.3	E	850.4	425.7	833.4	832.4	7
5	612.4	306.7	595.3	594.4	I	721.4	361.2	704.4	703.4	6
6	683.4	342.2	666.4	665.4	A	608.3		591.3	590.3	5
7	811.5	406.2	794.4	793.5	Q	537.3		520.2	519.3	4
8	926.5	463.8	909.5	908.5	D	409.2		392.2	391.2	3
9	1,073.6	537.3	1,056.5	1,055.6	F	294.2		277.2		2
10	1,219.7	610.3	1,202.6	1,201.7	K	147.1		130.1		1

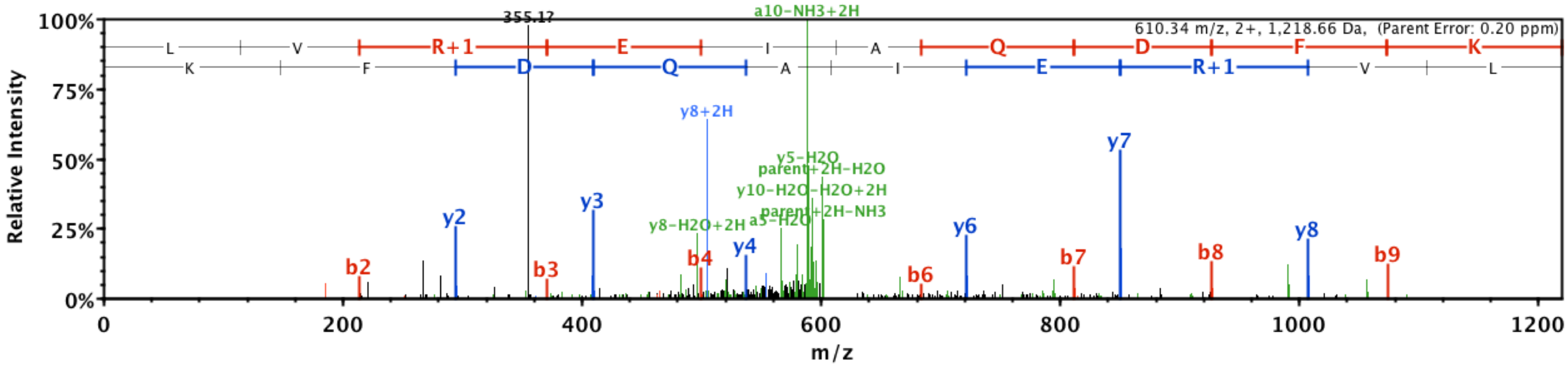

B	B Ions	B+2H	B-NH3	B-H2O	AA	Y Ions	Y+2H	Y-NH3	Y-H2O	Y
1	114.1				L	1,219.7	610.3	1,202.6	1,201.7	10
2	213.2				V	1,106.6	553.8	1,089.6	1,088.6	9
3	370.2	185.6	353.2		R+1	1,007.5	504.3	990.5	989.5	8
4	499.3	250.1	482.3	481.3	E	850.4	425.7	833.4	832.4	7
5	612.4	306.7	595.3	594.4	I	721.4	361.2	704.4	703.4	6
6	683.4	342.2	666.4	665.4	A	608.3		591.3	590.3	5
7	811.5	406.2	794.4	793.5	Q	537.3		520.2	519.3	4
8	926.5	463.8	909.5	908.5	D	409.2		392.2	391.2	3
9	1,073.6	537.3	1,056.5	1,055.6	F	294.2		277.2		2
10	1,219.7	610.3	1,202.6	1,201.7	K	147.1		130.1		1

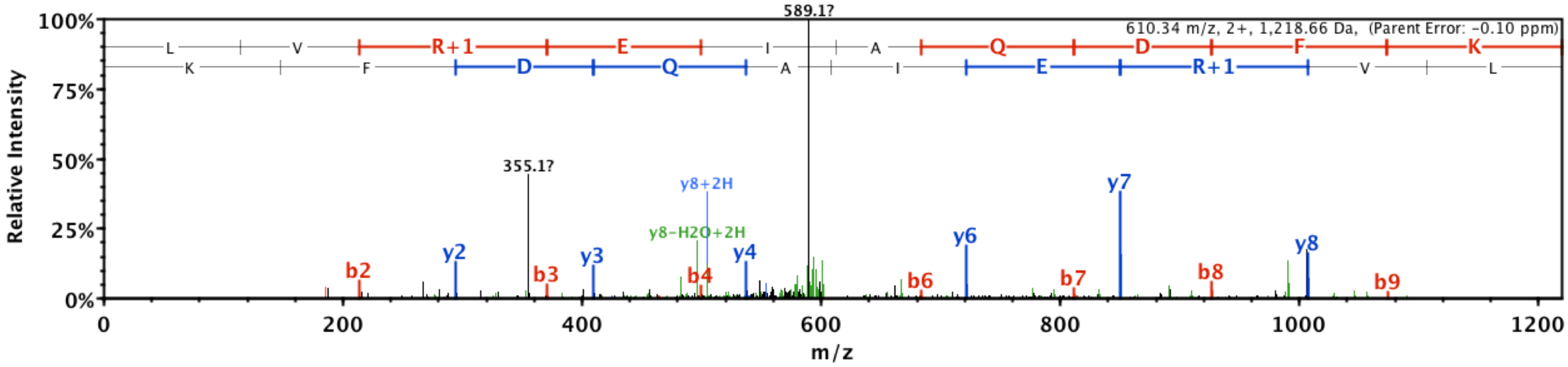

B	B Ions	B+2H	B-NH3	B-H2O	AA	Y Ions	Y+2H	Y-NH3	Y-H2O	Y
1	114.1				L	1,219.7	610.3	1,202.6	1,201.7	10
2	213.2				V	1,106.6	553.8	1,089.6	1,088.6	9
3	370.2	185.6	353.2		R+1	1,007.5	504.3	990.5	989.5	8
4	499.3	250.1	482.3	481.3	E	850.4	425.7	833.4	832.4	7
5	612.4	306.7	595.3	594.4	I	721.4	361.2	704.4	703.4	6
6	683.4	342.2	666.4	665.4	A	608.3		591.3	590.3	5
7	811.5	406.2	794.4	793.5	Q	537.3		520.2	519.3	4
8	926.5	463.8	909.5	908.5	D	409.2		392.2	391.2	3
9	1,073.6	537.3	1,056.5	1,055.6	F	294.2		277.2		2
10	1,219.7	610.3	1,202.6	1,201.7	K	147.1		130.1		1

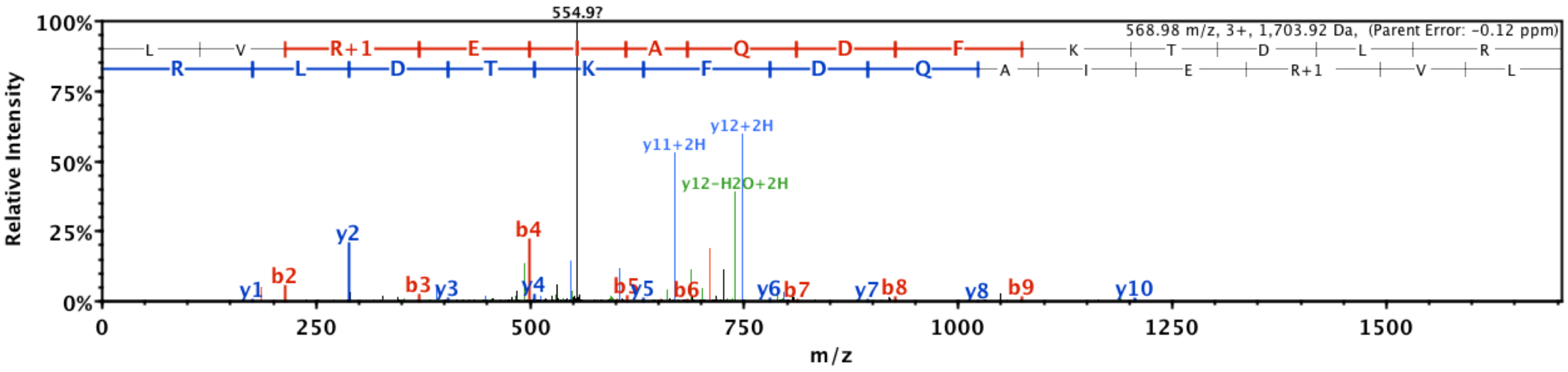

B	B Ions	B+2H	B-NH3	B-H2O	AA	Y Ions	Y+2H	Y-NH3	Y-H2O	Y
1	114.1	57.5			L	1,704.9	853.0	1,687.9	1,686.9	14
2	213.2	107.1			V	1,591.8	796.4	1,574.8	1,573.8	13
3	370.2	185.6	353.2		R+1	1,492.8	746.9	1,475.7	1,474.8	12
4	499.3	250.1	482.3	481.3	E	1,335.7	668.3	1,318.7	1,317.7	11
5	612.4	306.7	595.3	594.4	I	1,206.6	603.8	1,189.6	1,188.6	10
6	683.4	342.2	666.4	665.4	A	1,093.6	547.3	1,076.5	1,075.6	9
7	811.5	406.2	794.4	793.5	Q	1,022.5	511.8	1,005.5	1,004.5	8
8	926.5	463.8	909.5	908.5	D	894.5	447.7	877.4	876.5	7
9	1,073.6	537.3	1,056.5	1,055.6	F	779.4	390.2	762.4	761.4	6
10	1,201.7	601.3	1,184.6	1,183.6	K	632.4	316.7	615.3	614.4	5
11	1,302.7	651.9	1,285.7	1,284.7	T	504.3	252.6	487.3	486.3	4
12	1,417.7	709.4	1,400.7	1,399.7	D	403.2	202.1	386.2	385.2	3
13	1,530.8	765.9	1,513.8	1,512.8	L	288.2	144.6	271.2		2
14	1,704.9	853.0	1,687.9	1,686.9	R	175.1	88.1	158.1		1

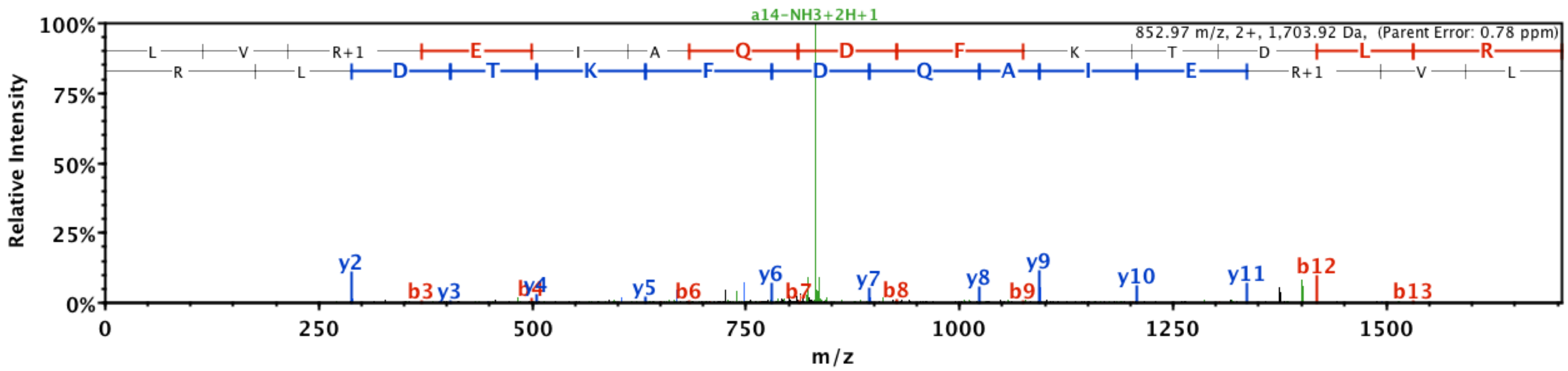

B	B Ions	B+2H	B-NH3	B-H2O	AA	Y Ions	Y+2H	Y-NH3	Y-H2O	Y
1	114.1				L	1,704.9	853.0	1,687.9	1,686.9	14
2	213.2				V	1,591.8	796.4	1,574.8	1,573.8	13
3	370.2	185.6	353.2		R+1	1,492.8	746.9	1,475.7	1,474.8	12
4	499.3	250.1	482.3	481.3	E	1,335.7	668.3	1,318.7	1,317.7	11
5	612.4	306.7	595.3	594.4	I	1,206.6	603.8	1,189.6	1,188.6	10
6	683.4	342.2	666.4	665.4	A	1,093.6	547.3	1,076.5	1,075.6	9
7	811.5	406.2	794.4	793.5	Q	1,022.5	511.8	1,005.5	1,004.5	8
8	926.5	463.8	909.5	908.5	D	894.5	447.7	877.4	876.5	7
9	1,073.6	537.3	1,056.5	1,055.6	F	779.4	390.2	762.4	761.4	6
10	1,201.7	601.3	1,184.6	1,183.6	K	632.4	316.7	615.3	614.4	5
11	1,302.7	651.9	1,285.7	1,284.7	T	504.3		487.3	486.3	4
12	1,417.7	709.4	1,400.7	1,399.7	D	403.2		386.2	385.2	3
13	1,530.8	765.9	1,513.8	1,512.8	L	288.2		271.2		2
14	1,704.9	853.0	1,687.9	1,686.9	R	175.1		158.1		1

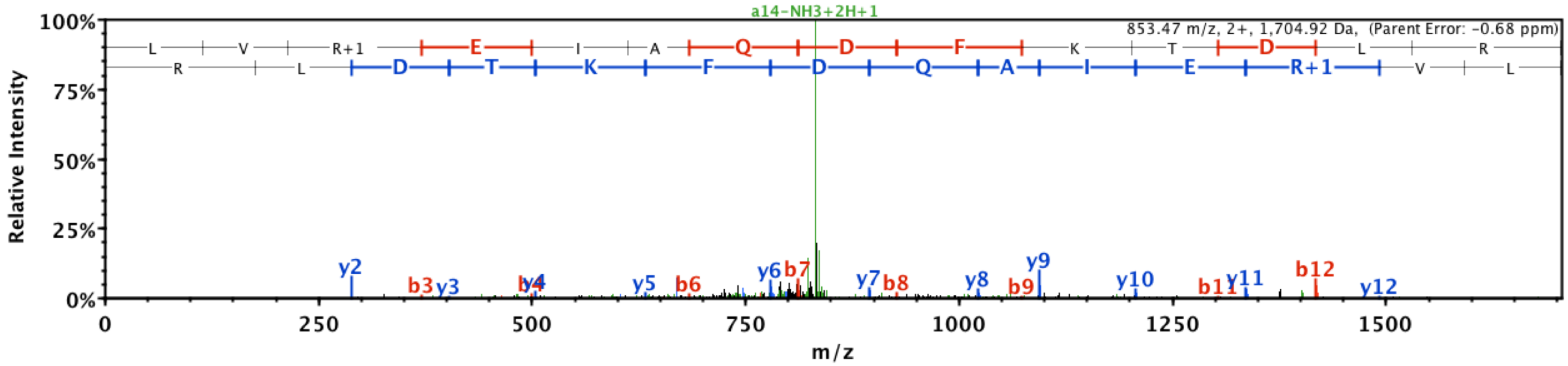

B	B Ions	B+2H	B-NH3	B-H2O	AA	Y Ions	Y+2H	Y-NH3	Y-H2O	Y
1	114.1				L	1,704.9	853.0	1,687.9	1,686.9	14
2	213.2				V	1,591.8	796.4	1,574.8	1,573.8	13
3	370.2	185.6	353.2		R+1	1,492.8	746.9	1,475.7	1,474.8	12
4	499.3	250.1	482.3	481.3	E	1,335.7	668.3	1,318.7	1,317.7	11
5	612.4	306.7	595.3	594.4	I	1,206.6	603.8	1,189.6	1,188.6	10
6	683.4	342.2	666.4	665.4	A	1,093.6	547.3	1,076.5	1,075.6	9
7	811.5	406.2	794.4	793.5	Q	1,022.5	511.8	1,005.5	1,004.5	8
8	926.5	463.8	909.5	908.5	D	894.5	447.7	877.4	876.5	7
9	1,073.6	537.3	1,056.5	1,055.6	F	779.4	390.2	762.4	761.4	6
10	1,201.7	601.3	1,184.6	1,183.6	K	632.4	316.7	615.3	614.4	5
11	1,302.7	651.9	1,285.7	1,284.7	T	504.3		487.3	486.3	4
12	1,417.7	709.4	1,400.7	1,399.7	D	403.2		386.2	385.2	3
13	1,530.8	765.9	1,513.8	1,512.8	L	288.2		271.2		2
14	1,704.9	853.0	1,687.9	1,686.9	R	175.1		158.1		1

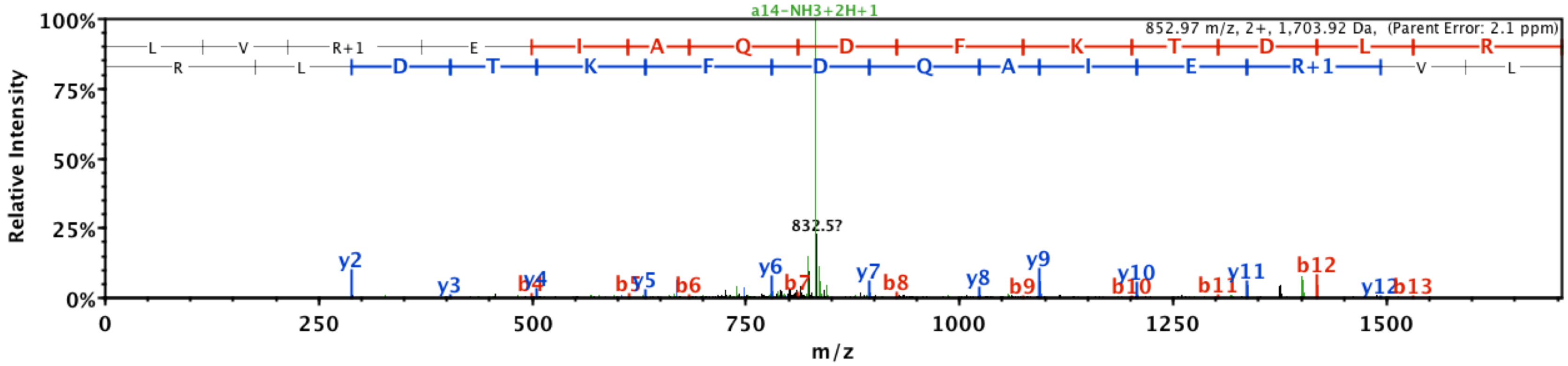

B	B Ions	B+2H	B-NH3	B-H2O	AA	Y Ions	Y+2H	Y-NH3	Y-H2O	Y
1	114.1				L	1,704.9	853.0	1,687.9	1,686.9	14
2	213.2				V	1,591.8	796.4	1,574.8	1,573.8	13
3	370.2	185.6	353.2		R+1	1,492.8	746.9	1,475.7	1,474.8	12
4	499.3	250.1	482.3	481.3	E	1,335.7	668.3	1,318.7	1,317.7	11
5	612.4	306.7	595.3	594.4	I	1,206.6	603.8	1,189.6	1,188.6	10
6	683.4	342.2	666.4	665.4	A	1,093.6	547.3	1,076.5	1,075.6	9
7	811.5	406.2	794.4	793.5	Q	1,022.5	511.8	1,005.5	1,004.5	8
8	926.5	463.8	909.5	908.5	D	894.5	447.7	877.4	876.5	7
9	1,073.6	537.3	1,056.5	1,055.6	F	779.4	390.2	762.4	761.4	6
10	1,201.7	601.3	1,184.6	1,183.6	K	632.4	316.7	615.3	614.4	5
11	1,302.7	651.9	1,285.7	1,284.7	T	504.3		487.3	486.3	4
12	1,417.7	709.4	1,400.7	1,399.7	D	403.2		386.2	385.2	3
13	1,530.8	765.9	1,513.8	1,512.8	L	288.2		271.2		2
14	1,704.9	853.0	1,687.9	1,686.9	R	175.1		158.1		1

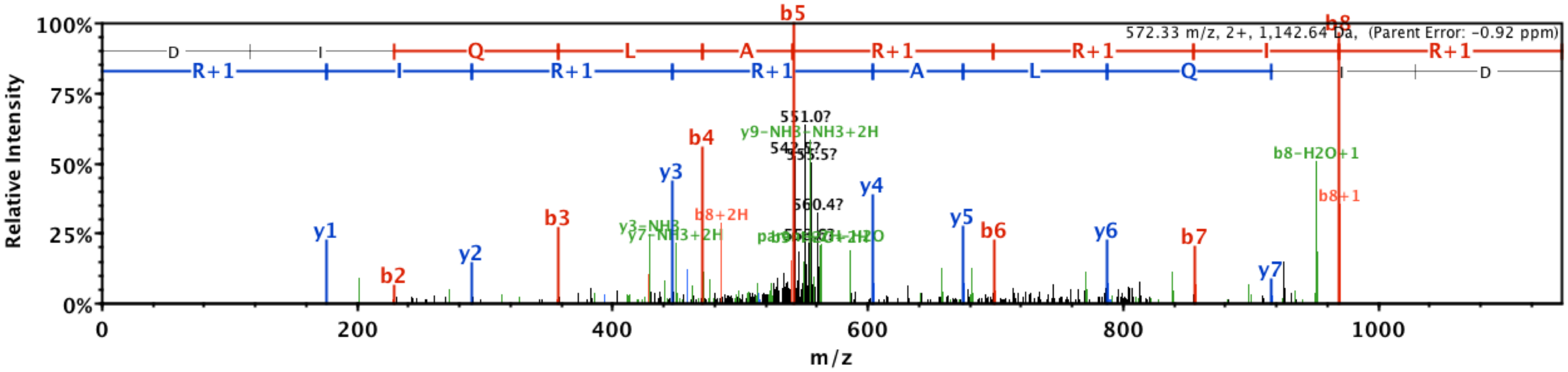

B	B Ions	B+2H	B-NH3	B-H2O	AA	Y Ions	Y+2H	Y-NH3	Y-H2O	Y
1	116.0			98.0	D	1,143.6	572.3	1,126.6	1,125.6	9
2	229.1			211.1	I	1,028.6	514.8	1,011.6		8
3	357.2		340.2	339.2	Q	915.5	458.3	898.5		7
4	470.3		453.2	452.3	L	787.5	394.2	770.5		6
5	541.3		524.3	523.3	A	674.4	337.7	657.4		5
6	698.4	349.7	681.4	680.4	R+1	603.4	302.2	586.3		4
7	855.5	428.2	838.4	837.5	R+1	446.3	223.6	429.2		3
8	968.6	484.8	951.5	950.5	I	289.2		272.2		2
9	1,143.6	572.3	1,126.6	1,125.6	R+1	176.1		159.1		1

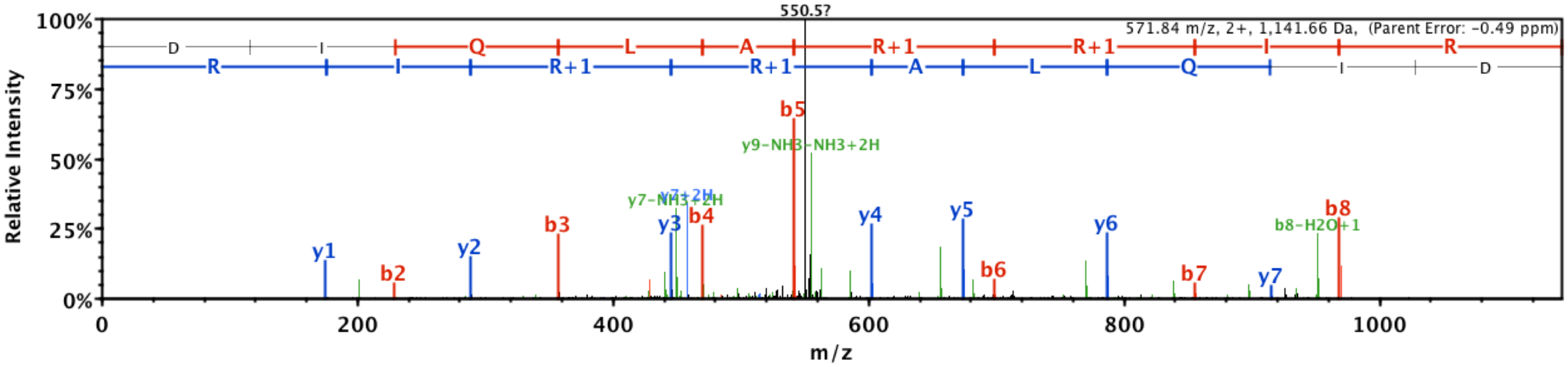

B	B Ions	B+2H	B-NH3	B-H2O	AA	Y Ions	Y+2H	Y-NH3	Y-H2O	Y
1	116.0			98.0	D	1,142.7	571.8	1,125.6	1,124.7	9
2	229.1			211.1	I	1,027.6	514.3	1,010.6		8
3	357.2		340.2	339.2	Q	914.6	457.8	897.5		7
4	470.3		453.2	452.3	L	786.5	393.8	769.5		6
5	541.3		524.3	523.3	A	673.4	337.2	656.4		5
6	698.4	349.7	681.4	680.4	R+1	602.4	301.7	585.3		4
7	855.5	428.2	838.4	837.5	R+1	445.3	223.1	428.3		3
8	968.6	484.8	951.5	950.5	I	288.2		271.2		2
9	1,142.7	571.8	1,125.6	1,124.7	R	175.1		158.1		1

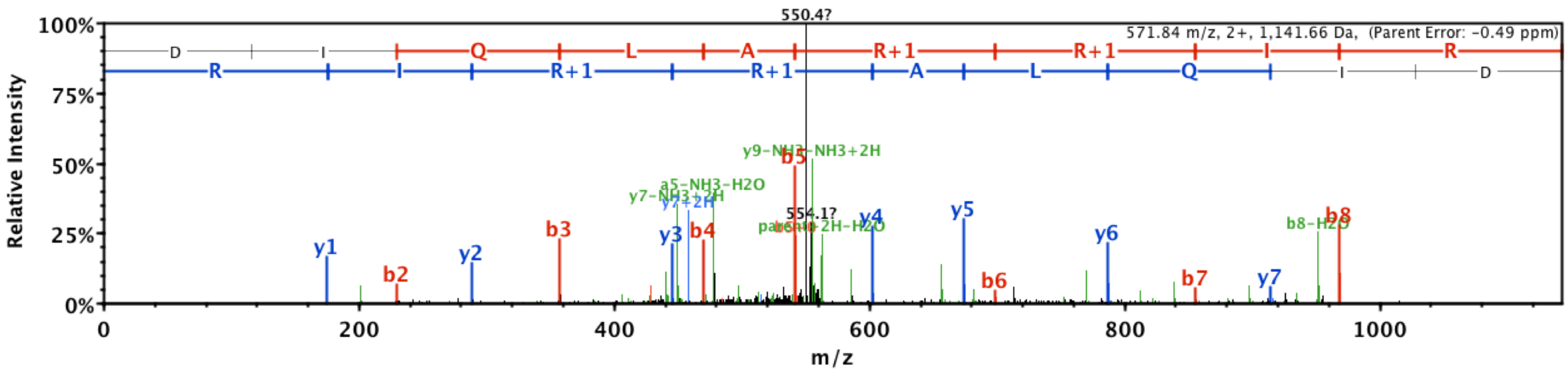

B	B Ions	B+2H	B-NH3	B-H2O	AA	Y Ions	Y+2H	Y-NH3	Y-H2O	Y
1	116.0			98.0	D	1,142.7	571.8	1,125.6	1,124.7	9
2	229.1			211.1	I	1,027.6	514.3	1,010.6		8
3	357.2		340.2	339.2	Q	914.6	457.8	897.5		7
4	470.3		453.2	452.3	L	786.5	393.8	769.5		6
5	541.3		524.3	523.3	A	673.4	337.2	656.4		5
6	698.4	349.7	681.4	680.4	R+1	602.4	301.7	585.3		4
7	855.5	428.2	838.4	837.5	R+1	445.3	223.1	428.3		3
8	968.6	484.8	951.5	950.5	I	288.2		271.2		2
9	1,142.7	571.8	1,125.6	1,124.7	R	175.1		158.1		1

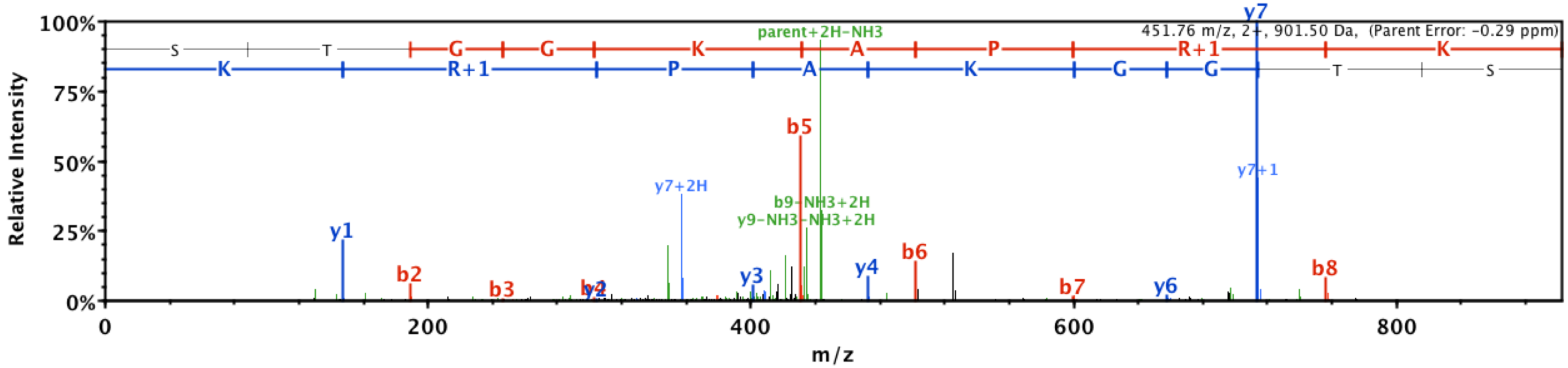

B	B Ions	B+2H	B-NH3	B-H2O	AA	Y Ions	Y+2H	Y-NH3	Y-H2O	Y
1	88.0			70.0	S	902.5	451.8	885.5	884.5	9
2	189.1			171.1	T	815.5	408.2	798.4	797.5	8
3	246.1			228.1	G	714.4	357.7	697.4		7
4	303.1			285.1	G	657.4	329.2	640.4		6
5	431.2	216.1	414.2	413.2	K	600.4	300.7	583.4		5
6	502.3	251.6	485.2	484.3	A	472.3	236.6	455.3		4
7	599.3	300.2	582.3	581.3	P	401.3	201.1	384.2		3
8	756.4	378.7	739.4	738.4	R+1	304.2	152.6	287.2		2
9	902.5	451.8	885.5	884.5	K	147.1		130.1		1

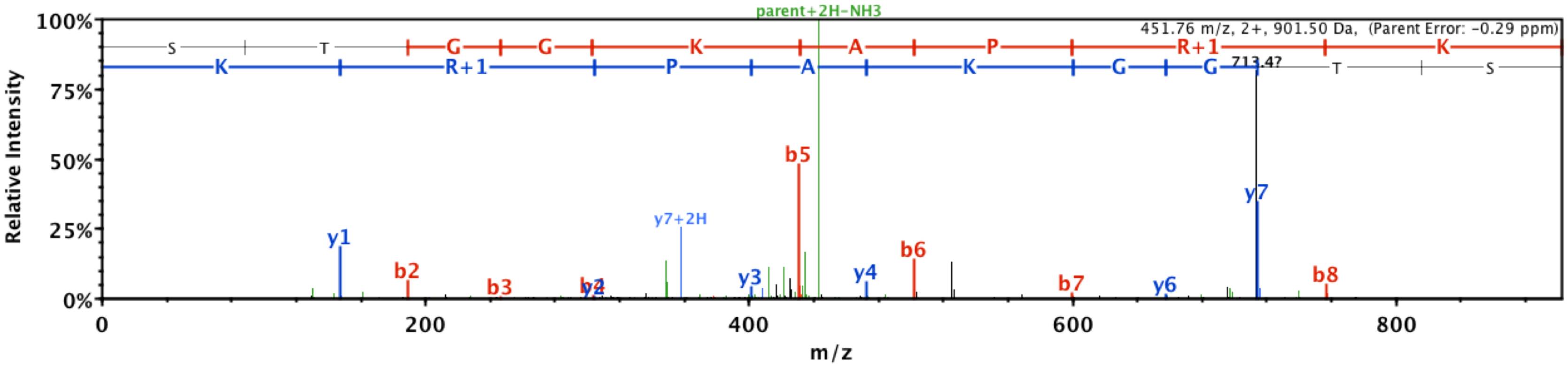

B	B Ions	B+2H	B-NH3	B-H2O	AA	Y Ions	Y+2H	Y-NH3	Y-H2O	Y
1	88.0			70.0	S	902.5	451.8	885.5	884.5	9
2	189.1			171.1	T	815.5	408.2	798.4	797.5	8
3	246.1			228.1	G	714.4	357.7	697.4		7
4	303.1			285.1	G	657.4	329.2	640.4		6
5	431.2	216.1	414.2	413.2	K	600.4	300.7	583.4		5
6	502.3	251.6	485.2	484.3	A	472.3	236.6	455.3		4
7	599.3	300.2	582.3	581.3	P	401.3	201.1	384.2		3
8	756.4	378.7	739.4	738.4	R+1	304.2	152.6	287.2		2
9	902.5	451.8	885.5	884.5	K	147.1		130.1		1

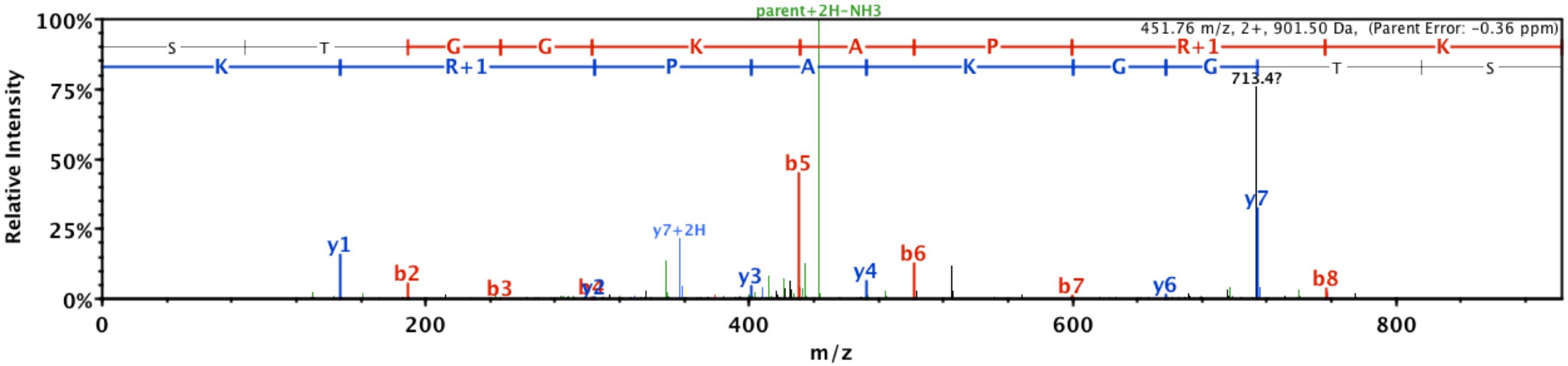

B	B Ions	B+2H	B-NH3	B-H2O	AA	Y Ions	Y+2H	Y-NH3	Y-H2O	Y
1	88.0			70.0	S	902.5	451.8	885.5	884.5	9
2	189.1			171.1	T	815.5	408.2	798.4	797.5	8
3	246.1			228.1	G	714.4	357.7	697.4		7
4	303.1			285.1	G	657.4	329.2	640.4		6
5	431.2	216.1	414.2	413.2	K	600.4	300.7	583.4		5
6	502.3	251.6	485.2	484.3	A	472.3	236.6	455.3		4
7	599.3	300.2	582.3	581.3	P	401.3	201.1	384.2		3
8	756.4	378.7	739.4	738.4	R+1	304.2	152.6	287.2		2
9	902.5	451.8	885.5	884.5	K	147.1		130.1		1

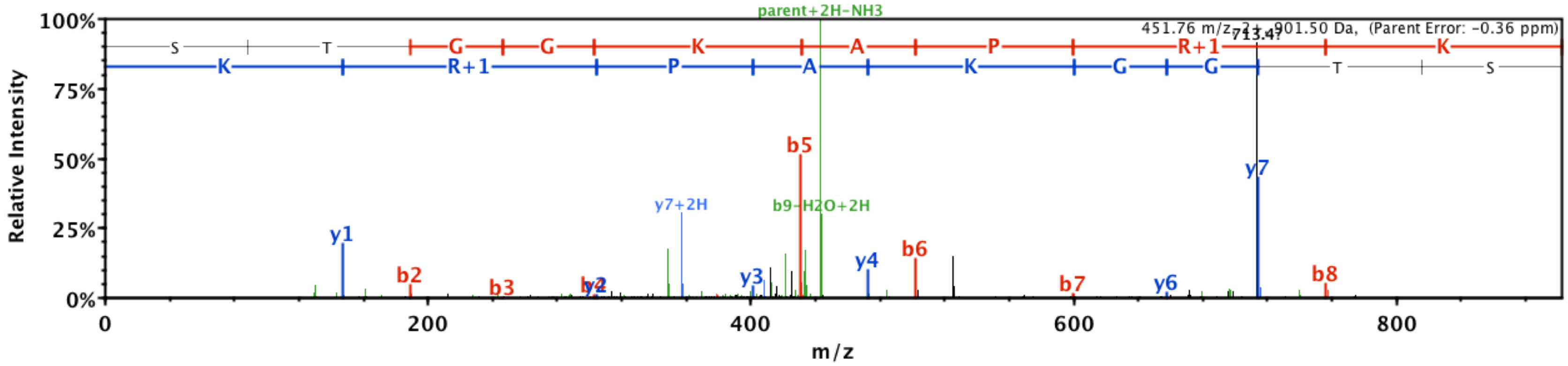

B	B Ions	B+2H	B-NH3	B-H2O	AA	Y Ions	Y+2H	Y-NH3	Y-H2O	Y
1	88.0			70.0	S	902.5	451.8	885.5	884.5	9
2	189.1			171.1	T	815.5	408.2	798.4	797.5	8
3	246.1			228.1	G	714.4	357.7	697.4		7
4	303.1			285.1	G	657.4	329.2	640.4		6
5	431.2	216.1	414.2	413.2	K	600.4	300.7	583.4		5
6	502.3	251.6	485.2	484.3	A	472.3	236.6	455.3		4
7	599.3	300.2	582.3	581.3	P	401.3	201.1	384.2		3
8	756.4	378.7	739.4	738.4	R+1	304.2	152.6	287.2		2
9	902.5	451.8	885.5	884.5	K	147.1		130.1		1

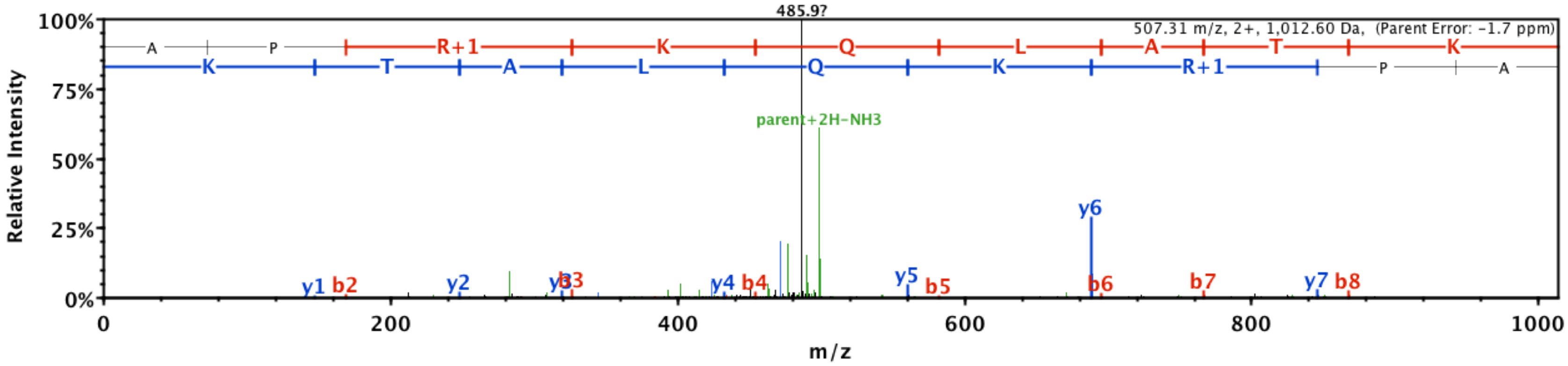

B	B Ions	B+2H	B-NH3	B-H2O	AA	Y Ions	Y+2H	Y-NH3	Y-H2O	Y
1	72.0				A	1,013.6	507.3	996.6	995.6	9
2	169.1				P	942.6	471.8	925.5	924.6	8
3	326.2	163.6	309.2		R+1	845.5	423.3	828.5	827.5	7
4	454.3	227.6	437.3		K	688.4	344.7	671.4	670.4	6
5	582.3	291.7	565.3		Q	560.3		543.3	542.3	5
6	695.4	348.2	678.4		L	432.3		415.3	414.3	4
7	766.5	383.7	749.4		A	319.2		302.2	301.2	3
8	867.5	434.3	850.5	849.5	T	248.2		231.1	230.1	2
9	1,013.6	507.3	996.6	995.6	K	147.1		130.1		1

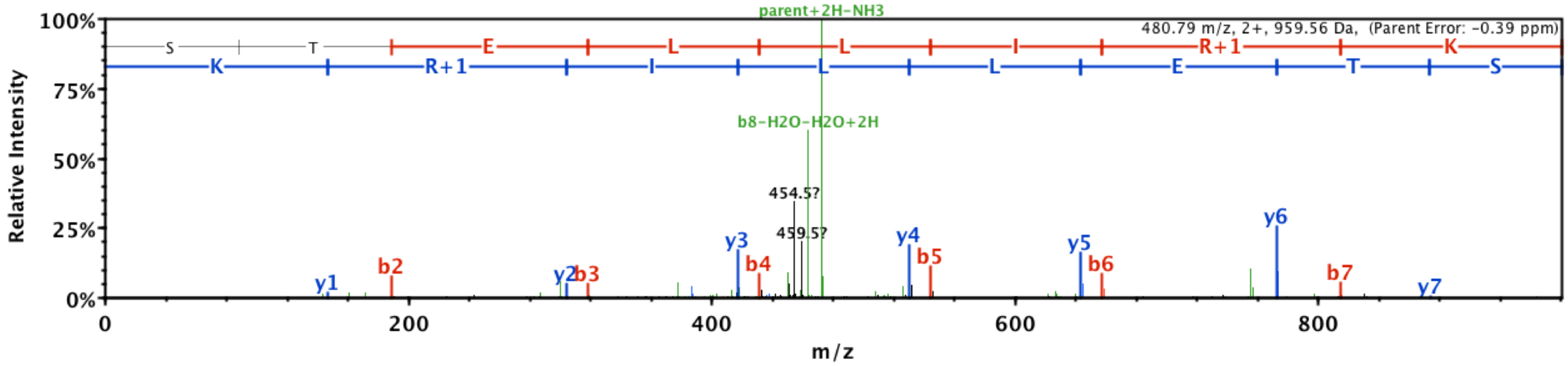

B	B Ions	B+2H	B-NH3	B-H2O	AA	Y Ions	Y+2H	Y-NH3	Y-H2O	Y
1	88.0			70.0	S	960.6	480.8	943.5	942.6	8
2	189.1			171.1	T	873.5	437.3	856.5	855.5	7
3	318.1			300.1	E	772.5	386.8	755.5	754.5	6
4	431.2			413.2	L	643.5	322.2	626.4		5
5	544.3			526.3	L	530.4	265.7	513.3		4
6	657.4	329.2		639.4	I	417.3	209.1	400.3		3
7	814.5	407.7	797.4	796.5	R+1	304.2	152.6	287.2		2
8	960.6	480.8	943.5	942.6	K	147.1		130.1		1

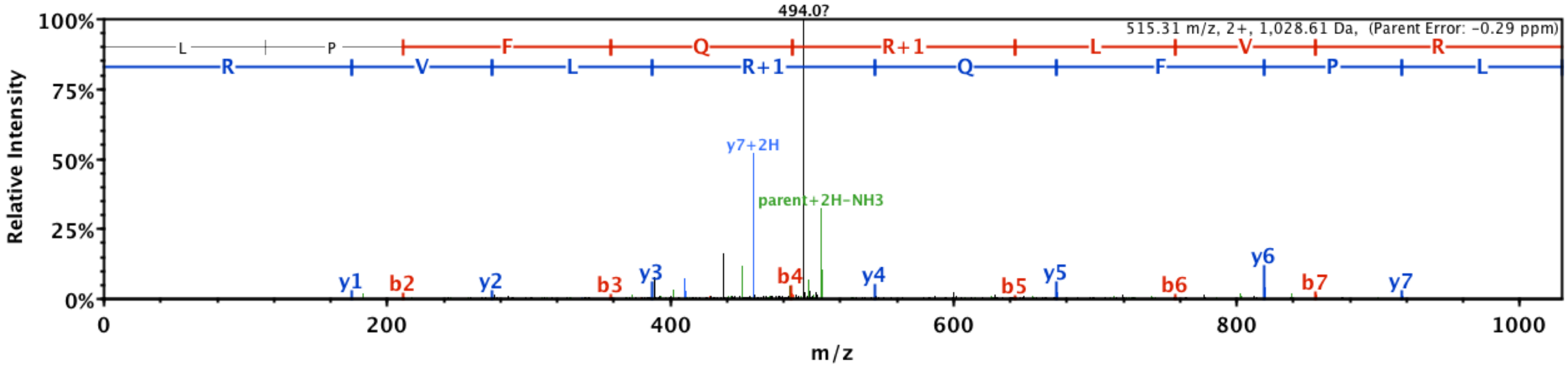

B	B Ions	B+2H	B-NH3	B-H2O	AA	Y Ions	Y+2H	Y-NH3	Y-H2O	Y
1	114.1				L	1,029.6	515.3	1,012.6		8
2	211.1				P	916.5	458.8	899.5		7
3	358.2				F	819.5	410.2	802.5		6
4	486.3		469.2		Q	672.4	336.7	655.4		5
5	643.4	322.2	626.3		R+1	544.4	272.7	527.3		4
6	756.4	378.7	739.4		L	387.3		370.2		3
7	855.5	428.3	838.5		V	274.2		257.2		2
8	1,029.6	515.3	1,012.6		R	175.1		158.1		1

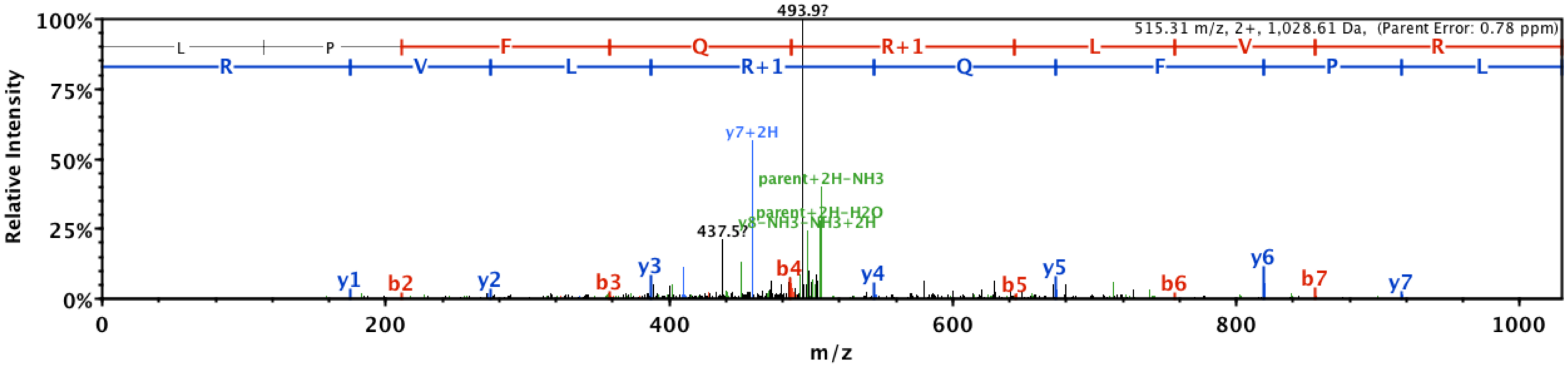

B	B Ions	B+2H	B-NH3	B-H2O	AA	Y Ions	Y+2H	Y-NH3	Y-H2O	Y
1	114.1				L	1,029.6	515.3	1,012.6		8
2	211.1				P	916.5	458.8	899.5		7
3	358.2				F	819.5	410.2	802.5		6
4	486.3		469.2		Q	672.4	336.7	655.4		5
5	643.4	322.2	626.3		R+1	544.4	272.7	527.3		4
6	756.4	378.7	739.4		L	387.3		370.2		3
7	855.5	428.3	838.5		V	274.2		257.2		2
8	1,029.6	515.3	1,012.6		R	175.1		158.1		1

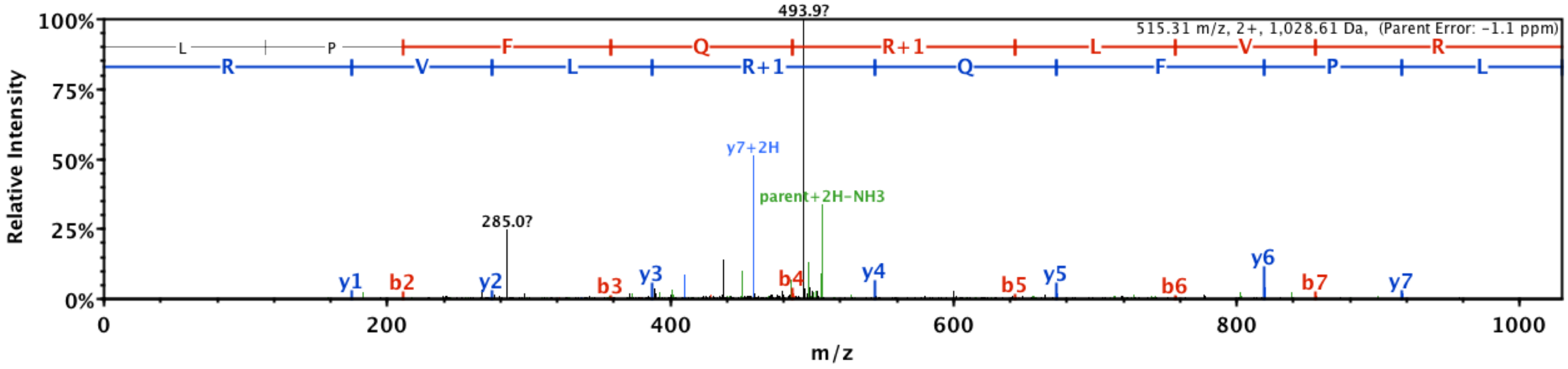

B	B Ions	B+2H	B-NH3	B-H2O	AA	Y Ions	Y+2H	Y-NH3	Y-H2O	Y
1	114.1				L	1,029.6	515.3	1,012.6		8
2	211.1				P	916.5	458.8	899.5		7
3	358.2				F	819.5	410.2	802.5		6
4	486.3		469.2		Q	672.4	336.7	655.4		5
5	643.4	322.2	626.3		R+1	544.4	272.7	527.3		4
6	756.4	378.7	739.4		L	387.3		370.2		3
7	855.5	428.3	838.5		V	274.2		257.2		2
8	1,029.6	515.3	1,012.6		R	175.1		158.1		1

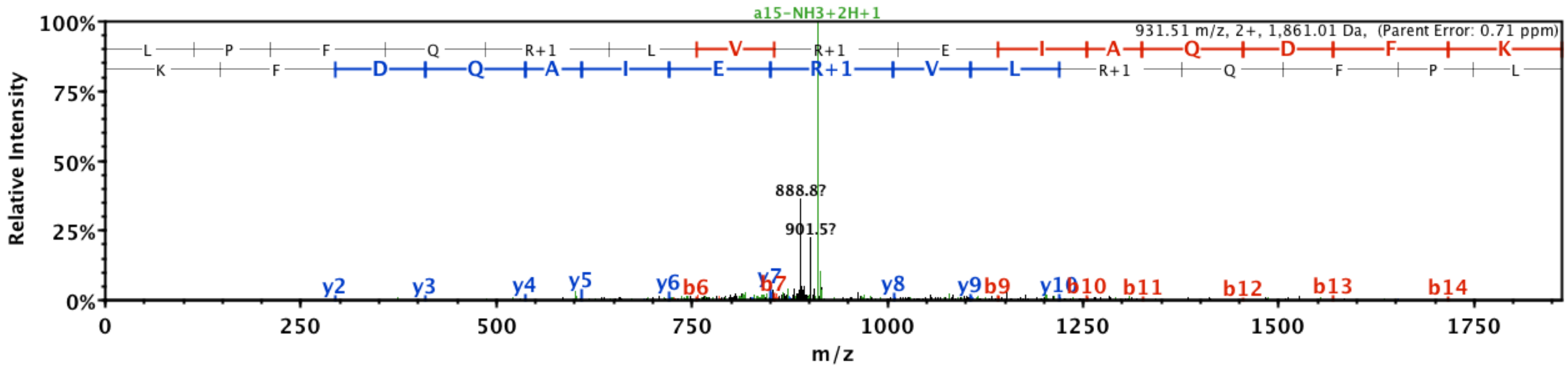

B	B Ions	B+2H	B-NH3	B-H2O	AA	Y Ions	Y+2H	Y-NH3	Y-H2O	Y
1	114.1				L	1,862.0	931.5	1,845.0	1,844.0	15
2	211.1				P	1,748.9	875.0	1,731.9	1,730.9	14
3	358.2				F	1,651.9	826.4	1,634.9	1,633.9	13
4	486.3		469.2		Q	1,504.8	752.9	1,487.8	1,486.8	12
5	643.4	322.2	626.3		R+1	1,376.8	688.9	1,359.7	1,358.7	11
6	756.4	378.7	739.4		L	1,219.7	610.3	1,202.6	1,201.7	10
7	855.5	428.3	838.5		V	1,106.6	553.8	1,089.6	1,088.6	9
8	1,012.6	506.8	995.6		R+1	1,007.5	504.3	990.5	989.5	8
9	1,141.6	571.3	1,124.6	1,123.6	E	850.4	425.7	833.4	832.4	7
10	1,254.7	627.9	1,237.7	1,236.7	I	721.4	361.2	704.4	703.4	6
11	1,325.8	663.4	1,308.7	1,307.7	A	608.3		591.3	590.3	5
12	1,453.8	727.4	1,436.8	1,435.8	Q	537.3		520.2	519.3	4
13	1,568.8	784.9	1,551.8	1,550.8	D	409.2		392.2	391.2	3
14	1,715.9	858.5	1,698.9	1,697.9	F	294.2		277.2		2
15	1,862.0	931.5	1,845.0	1,844.0	K	147.1		130.1		1

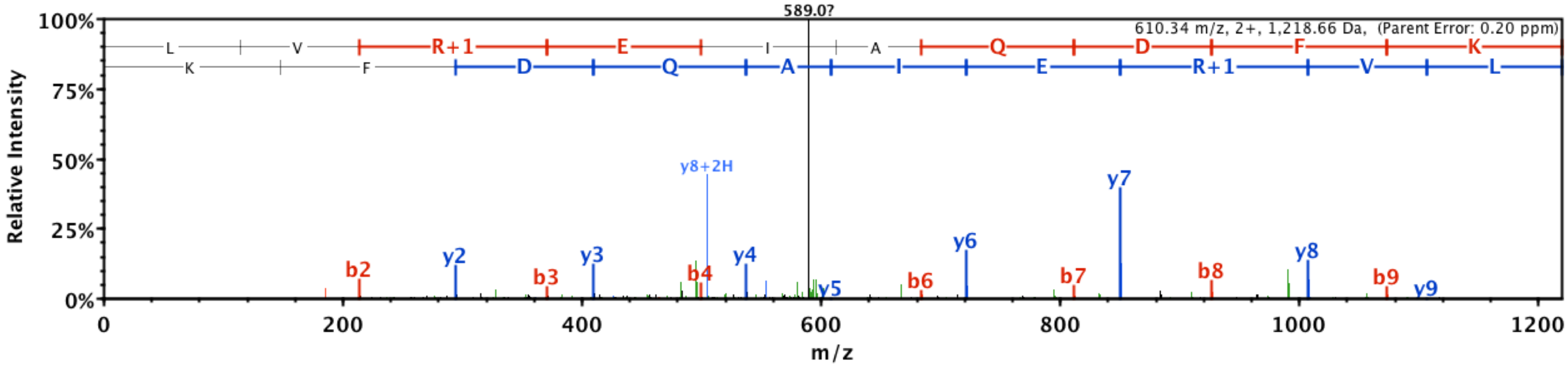

B	B Ions	B+2H	B-NH3	B-H2O	AA	Y Ions	Y+2H	Y-NH3	Y-H2O	Y
1	114.1				L	1,219.7	610.3	1,202.6	1,201.7	10
2	213.2				V	1,106.6	553.8	1,089.6	1,088.6	9
3	370.2	185.6	353.2		R+1	1,007.5	504.3	990.5	989.5	8
4	499.3	250.1	482.3	481.3	E	850.4	425.7	833.4	832.4	7
5	612.4	306.7	595.3	594.4	I	721.4	361.2	704.4	703.4	6
6	683.4	342.2	666.4	665.4	A	608.3		591.3	590.3	5
7	811.5	406.2	794.4	793.5	Q	537.3		520.2	519.3	4
8	926.5	463.8	909.5	908.5	D	409.2		392.2	391.2	3
9	1,073.6	537.3	1,056.5	1,055.6	F	294.2		277.2		2
10	1,219.7	610.3	1,202.6	1,201.7	K	147.1		130.1		1

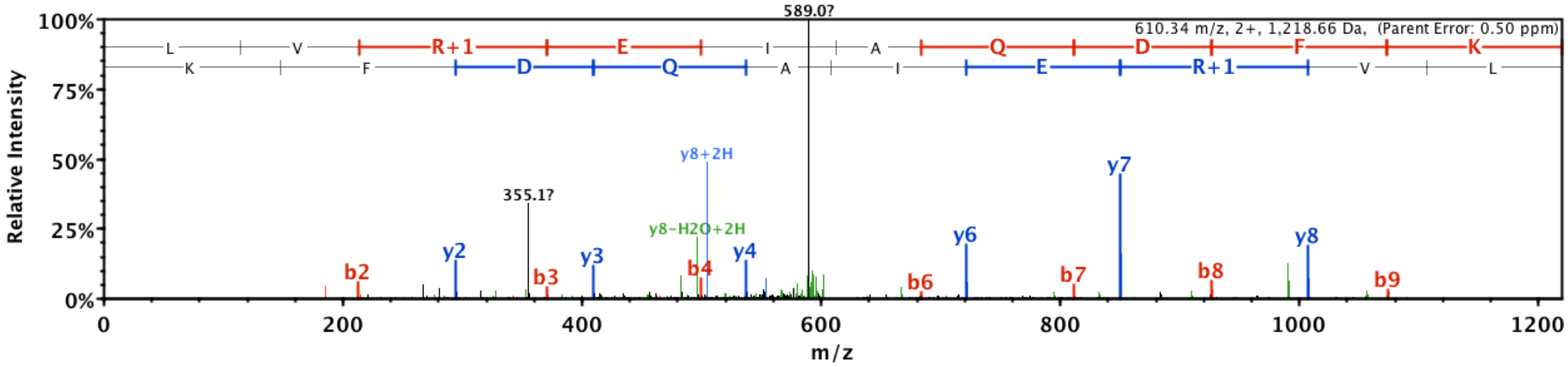

B	B Ions	B+2H	B-NH3	B-H2O	AA	Y Ions	Y+2H	Y-NH3	Y-H2O	Y
1	114.1				L	1,219.7	610.3	1,202.6	1,201.7	10
2	213.2				V	1,106.6	553.8	1,089.6	1,088.6	9
3	370.2	185.6	353.2		R+1	1,007.5	504.3	990.5	989.5	8
4	499.3	250.1	482.3	481.3	E	850.4	425.7	833.4	832.4	7
5	612.4	306.7	595.3	594.4	I	721.4	361.2	704.4	703.4	6
6	683.4	342.2	666.4	665.4	A	608.3		591.3	590.3	5
7	811.5	406.2	794.4	793.5	Q	537.3		520.2	519.3	4
8	926.5	463.8	909.5	908.5	D	409.2		392.2	391.2	3
9	1,073.6	537.3	1,056.5	1,055.6	F	294.2		277.2		2
10	1,219.7	610.3	1,202.6	1,201.7	K	147.1		130.1		1

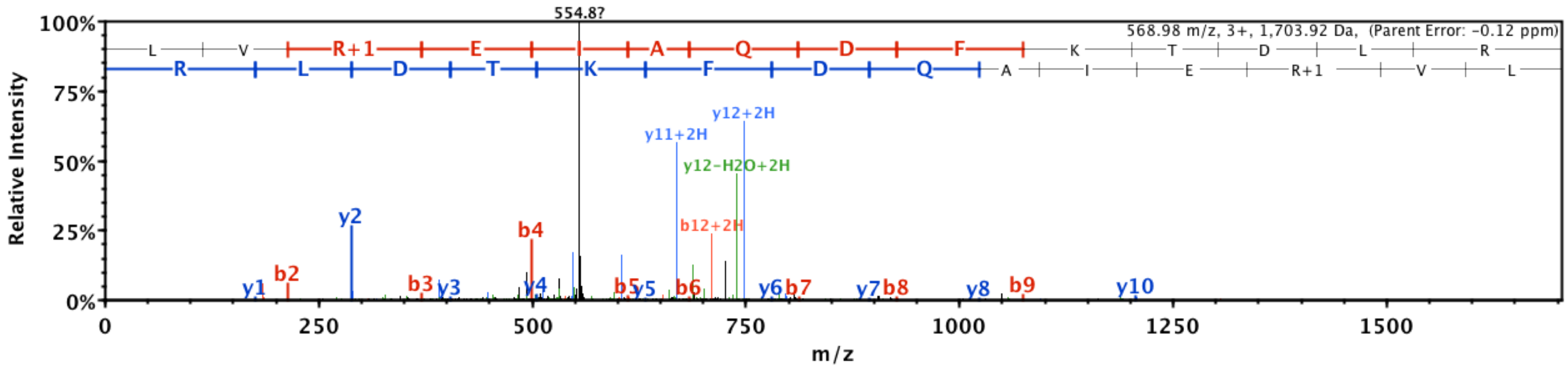

B	B Ions	B+2H	B-NH3	B-H2O	AA	Y Ions	Y+2H	Y-NH3	Y-H2O	Y
1	114.1	57.5			L	1,704.9	853.0	1,687.9	1,686.9	14
2	213.2	107.1			V	1,591.8	796.4	1,574.8	1,573.8	13
3	370.2	185.6	353.2		R+1	1,492.8	746.9	1,475.7	1,474.8	12
4	499.3	250.1	482.3	481.3	E	1,335.7	668.3	1,318.7	1,317.7	11
5	612.4	306.7	595.3	594.4	I	1,206.6	603.8	1,189.6	1,188.6	10
6	683.4	342.2	666.4	665.4	A	1,093.6	547.3	1,076.5	1,075.6	9
7	811.5	406.2	794.4	793.5	Q	1,022.5	511.8	1,005.5	1,004.5	8
8	926.5	463.8	909.5	908.5	D	894.5	447.7	877.4	876.5	7
9	1,073.6	537.3	1,056.5	1,055.6	F	779.4	390.2	762.4	761.4	6
10	1,201.7	601.3	1,184.6	1,183.6	K	632.4	316.7	615.3	614.4	5
11	1,302.7	651.9	1,285.7	1,284.7	T	504.3	252.6	487.3	486.3	4
12	1,417.7	709.4	1,400.7	1,399.7	D	403.2	202.1	386.2	385.2	3
13	1,530.8	765.9	1,513.8	1,512.8	L	288.2	144.6	271.2		2
14	1,704.9	853.0	1,687.9	1,686.9	R	175.1	88.1	158.1		1

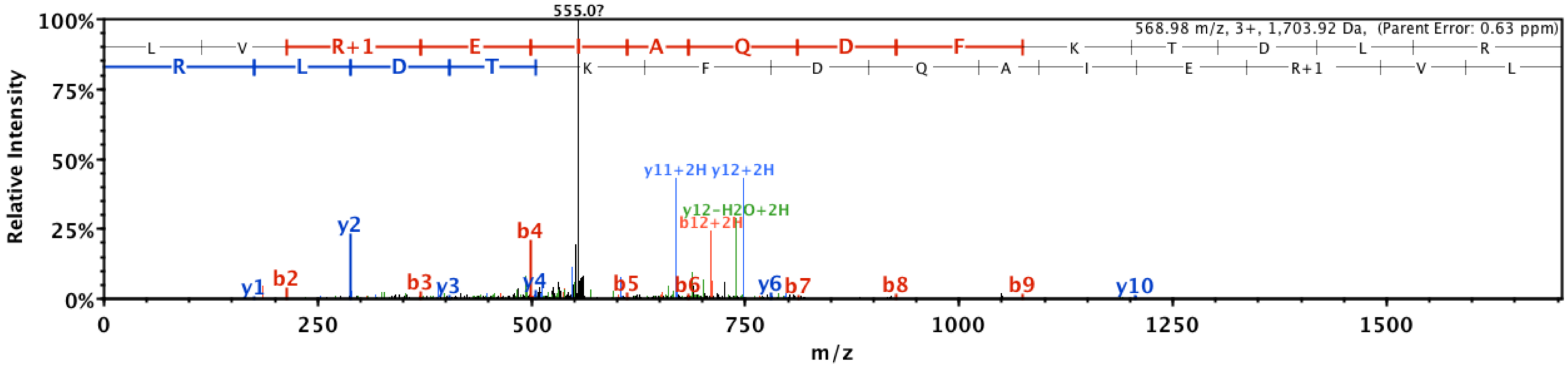

B	B Ions	B+2H	B-NH3	B-H2O	AA	Y Ions	Y+2H	Y-NH3	Y-H2O	Y
1	114.1	57.5			L	1,704.9	853.0	1,687.9	1,686.9	14
2	213.2	107.1			V	1,591.8	796.4	1,574.8	1,573.8	13
3	370.2	185.6	353.2		R+1	1,492.8	746.9	1,475.7	1,474.8	12
4	499.3	250.1	482.3	481.3	E	1,335.7	668.3	1,318.7	1,317.7	11
5	612.4	306.7	595.3	594.4	I	1,206.6	603.8	1,189.6	1,188.6	10
6	683.4	342.2	666.4	665.4	A	1,093.6	547.3	1,076.5	1,075.6	9
7	811.5	406.2	794.4	793.5	Q	1,022.5	511.8	1,005.5	1,004.5	8
8	926.5	463.8	909.5	908.5	D	894.5	447.7	877.4	876.5	7
9	1,073.6	537.3	1,056.5	1,055.6	F	779.4	390.2	762.4	761.4	6
10	1,201.7	601.3	1,184.6	1,183.6	K	632.4	316.7	615.3	614.4	5
11	1,302.7	651.9	1,285.7	1,284.7	T	504.3	252.6	487.3	486.3	4
12	1,417.7	709.4	1,400.7	1,399.7	D	403.2	202.1	386.2	385.2	3
13	1,530.8	765.9	1,513.8	1,512.8	L	288.2	144.6	271.2		2
14	1,704.9	853.0	1,687.9	1,686.9	R	175.1	88.1	158.1		1

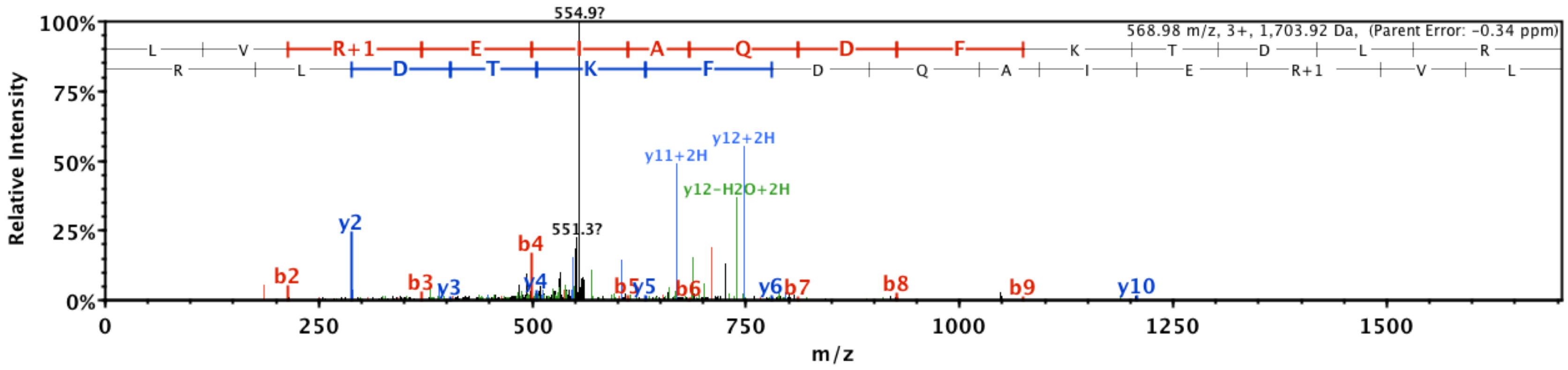

B	B Ions	B+2H	B-NH3	B-H2O	AA	Y Ions	Y+2H	Y-NH3	Y-H2O	Y
1	114.1	57.5			L	1,704.9	853.0	1,687.9	1,686.9	14
2	213.2	107.1			V	1,591.8	796.4	1,574.8	1,573.8	13
3	370.2	185.6	353.2		R+1	1,492.8	746.9	1,475.7	1,474.8	12
4	499.3	250.1	482.3	481.3	E	1,335.7	668.3	1,318.7	1,317.7	11
5	612.4	306.7	595.3	594.4	I	1,206.6	603.8	1,189.6	1,188.6	10
6	683.4	342.2	666.4	665.4	A	1,093.6	547.3	1,076.5	1,075.6	9
7	811.5	406.2	794.4	793.5	Q	1,022.5	511.8	1,005.5	1,004.5	8
8	926.5	463.8	909.5	908.5	D	894.5	447.7	877.4	876.5	7
9	1,073.6	537.3	1,056.5	1,055.6	F	779.4	390.2	762.4	761.4	6
10	1,201.7	601.3	1,184.6	1,183.6	K	632.4	316.7	615.3	614.4	5
11	1,302.7	651.9	1,285.7	1,284.7	T	504.3	252.6	487.3	486.3	4
12	1,417.7	709.4	1,400.7	1,399.7	D	403.2	202.1	386.2	385.2	3
13	1,530.8	765.9	1,513.8	1,512.8	L	288.2	144.6	271.2		2
14	1,704.9	853.0	1,687.9	1,686.9	R	175.1	88.1	158.1		1

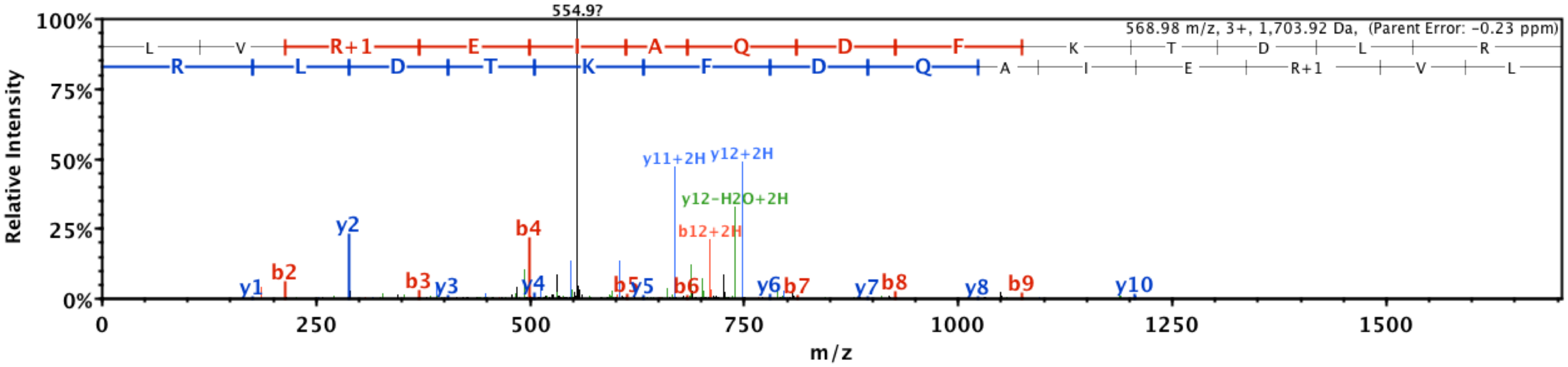

B	B Ions	B+2H	B-NH3	B-H2O	AA	Y Ions	Y+2H	Y-NH3	Y-H2O	Y
1	114.1	57.5			L	1,704.9	853.0	1,687.9	1,686.9	14
2	213.2	107.1			V	1,591.8	796.4	1,574.8	1,573.8	13
3	370.2	185.6	353.2		R+1	1,492.8	746.9	1,475.7	1,474.8	12
4	499.3	250.1	482.3	481.3	E	1,335.7	668.3	1,318.7	1,317.7	11
5	612.4	306.7	595.3	594.4	I	1,206.6	603.8	1,189.6	1,188.6	10
6	683.4	342.2	666.4	665.4	A	1,093.6	547.3	1,076.5	1,075.6	9
7	811.5	406.2	794.4	793.5	Q	1,022.5	511.8	1,005.5	1,004.5	8
8	926.5	463.8	909.5	908.5	D	894.5	447.7	877.4	876.5	7
9	1,073.6	537.3	1,056.5	1,055.6	F	779.4	390.2	762.4	761.4	6
10	1,201.7	601.3	1,184.6	1,183.6	K	632.4	316.7	615.3	614.4	5
11	1,302.7	651.9	1,285.7	1,284.7	T	504.3	252.6	487.3	486.3	4
12	1,417.7	709.4	1,400.7	1,399.7	D	403.2	202.1	386.2	385.2	3
13	1,530.8	765.9	1,513.8	1,512.8	L	288.2	144.6	271.2		2
14	1,704.9	853.0	1,687.9	1,686.9	R	175.1	88.1	158.1		1

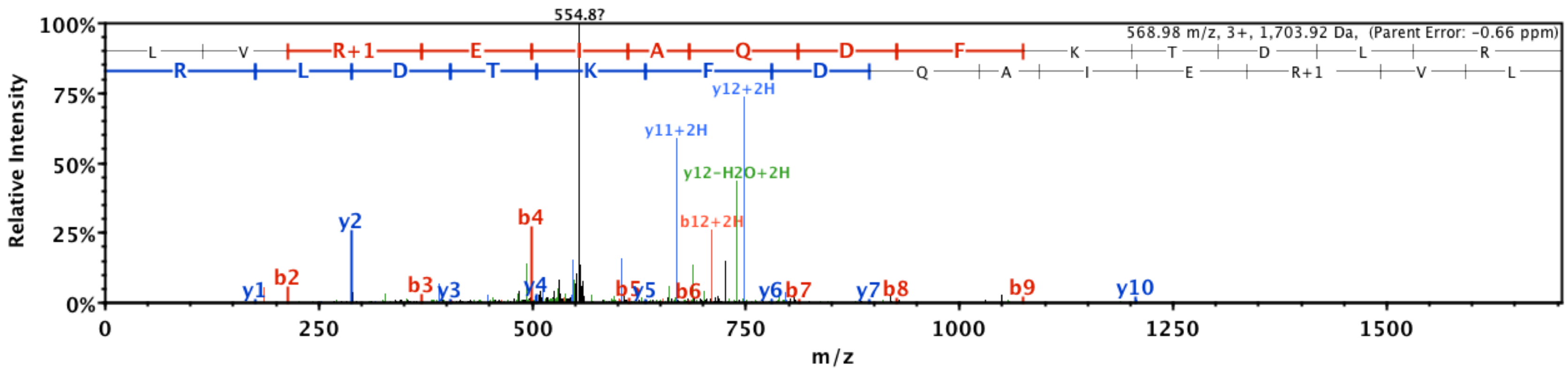

B	B Ions	B+2H	B-NH3	B-H2O	AA	Y Ions	Y+2H	Y-NH3	Y-H2O	Y
1	114.1	57.5			L	1,704.9	853.0	1,687.9	1,686.9	14
2	213.2	107.1			V	1,591.8	796.4	1,574.8	1,573.8	13
3	370.2	185.6	353.2		R+1	1,492.8	746.9	1,475.7	1,474.8	12
4	499.3	250.1	482.3	481.3	E	1,335.7	668.3	1,318.7	1,317.7	11
5	612.4	306.7	595.3	594.4	I	1,206.6	603.8	1,189.6	1,188.6	10
6	683.4	342.2	666.4	665.4	A	1,093.6	547.3	1,076.5	1,075.6	9
7	811.5	406.2	794.4	793.5	Q	1,022.5	511.8	1,005.5	1,004.5	8
8	926.5	463.8	909.5	908.5	D	894.5	447.7	877.4	876.5	7
9	1,073.6	537.3	1,056.5	1,055.6	F	779.4	390.2	762.4	761.4	6
10	1,201.7	601.3	1,184.6	1,183.6	K	632.4	316.7	615.3	614.4	5
11	1,302.7	651.9	1,285.7	1,284.7	T	504.3	252.6	487.3	486.3	4
12	1,417.7	709.4	1,400.7	1,399.7	D	403.2	202.1	386.2	385.2	3
13	1,530.8	765.9	1,513.8	1,512.8	L	288.2	144.6	271.2		2
14	1,704.9	853.0	1,687.9	1,686.9	R	175.1	88.1	158.1		1

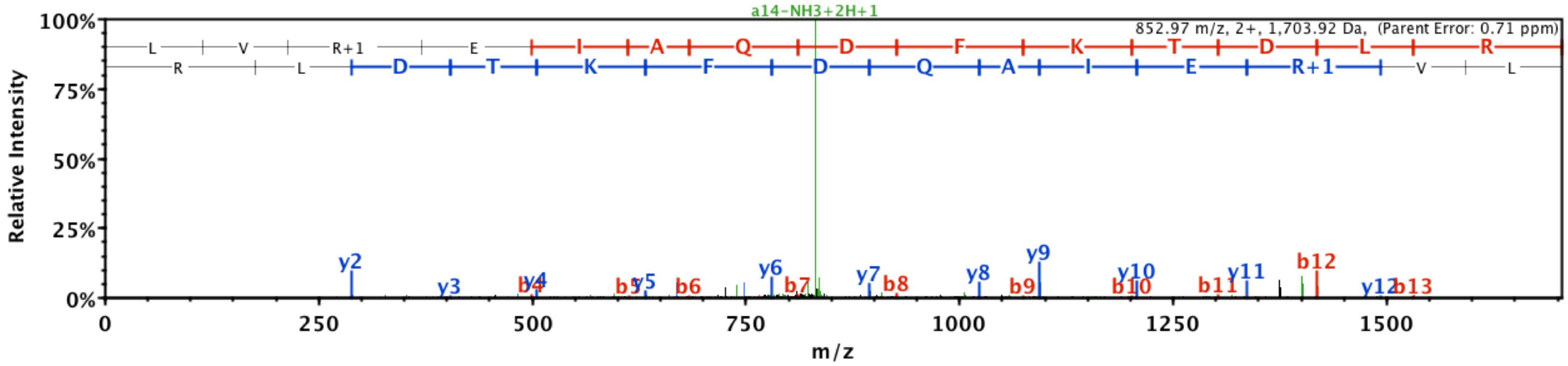

B	B Ions	B+2H	B-NH3	B-H2O	AA	Y Ions	Y+2H	Y-NH3	Y-H2O	Y
1	114.1				L	1,704.9	853.0	1,687.9	1,686.9	14
2	213.2				V	1,591.8	796.4	1,574.8	1,573.8	13
3	370.2	185.6	353.2		R+1	1,492.8	746.9	1,475.7	1,474.8	12
4	499.3	250.1	482.3	481.3	E	1,335.7	668.3	1,318.7	1,317.7	11
5	612.4	306.7	595.3	594.4	I	1,206.6	603.8	1,189.6	1,188.6	10
6	683.4	342.2	666.4	665.4	A	1,093.6	547.3	1,076.5	1,075.6	9
7	811.5	406.2	794.4	793.5	Q	1,022.5	511.8	1,005.5	1,004.5	8
8	926.5	463.8	909.5	908.5	D	894.5	447.7	877.4	876.5	7
9	1,073.6	537.3	1,056.5	1,055.6	F	779.4	390.2	762.4	761.4	6
10	1,201.7	601.3	1,184.6	1,183.6	K	632.4	316.7	615.3	614.4	5
11	1,302.7	651.9	1,285.7	1,284.7	T	504.3		487.3	486.3	4
12	1,417.7	709.4	1,400.7	1,399.7	D	403.2		386.2	385.2	3
13	1,530.8	765.9	1,513.8	1,512.8	L	288.2		271.2		2
14	1,704.9	853.0	1,687.9	1,686.9	R	175.1		158.1		1

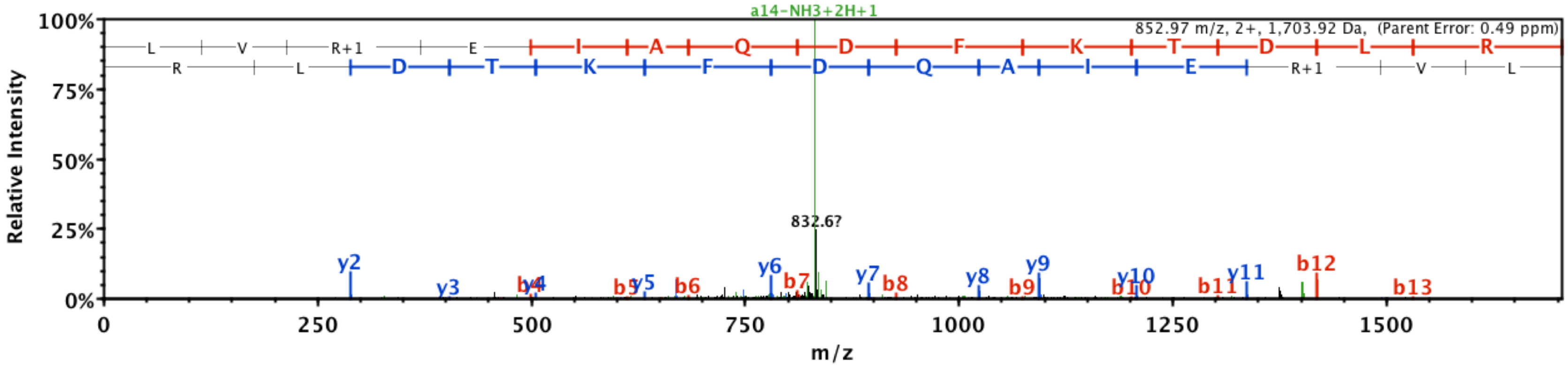

B	B Ions	B+2H	B-NH3	B-H2O	AA	Y Ions	Y+2H	Y-NH3	Y-H2O	Y
1	114.1				L	1,704.9	853.0	1,687.9	1,686.9	14
2	213.2				V	1,591.8	796.4	1,574.8	1,573.8	13
3	370.2	185.6	353.2		R+1	1,492.8	746.9	1,475.7	1,474.8	12
4	499.3	250.1	482.3	481.3	E	1,335.7	668.3	1,318.7	1,317.7	11
5	612.4	306.7	595.3	594.4	I	1,206.6	603.8	1,189.6	1,188.6	10
6	683.4	342.2	666.4	665.4	A	1,093.6	547.3	1,076.5	1,075.6	9
7	811.5	406.2	794.4	793.5	Q	1,022.5	511.8	1,005.5	1,004.5	8
8	926.5	463.8	909.5	908.5	D	894.5	447.7	877.4	876.5	7
9	1,073.6	537.3	1,056.5	1,055.6	F	779.4	390.2	762.4	761.4	6
10	1,201.7	601.3	1,184.6	1,183.6	K	632.4	316.7	615.3	614.4	5
11	1,302.7	651.9	1,285.7	1,284.7	T	504.3		487.3	486.3	4
12	1,417.7	709.4	1,400.7	1,399.7	D	403.2		386.2	385.2	3
13	1,530.8	765.9	1,513.8	1,512.8	L	288.2		271.2		2
14	1,704.9	853.0	1,687.9	1,686.9	R	175.1		158.1		1

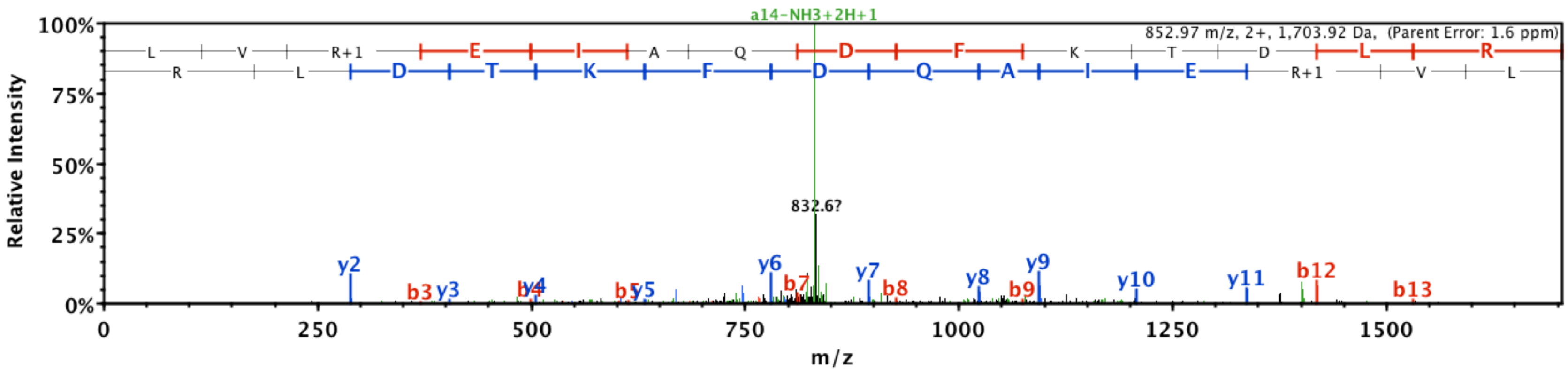

B	B Ions	B+2H	B-NH3	B-H2O	AA	Y Ions	Y+2H	Y-NH3	Y-H2O	Y
1	114.1				L	1,704.9	853.0	1,687.9	1,686.9	14
2	213.2				V	1,591.8	796.4	1,574.8	1,573.8	13
3	370.2	185.6	353.2		R+1	1,492.8	746.9	1,475.7	1,474.8	12
4	499.3	250.1	482.3	481.3	E	1,335.7	668.3	1,318.7	1,317.7	11
5	612.4	306.7	595.3	594.4	I	1,206.6	603.8	1,189.6	1,188.6	10
6	683.4	342.2	666.4	665.4	A	1,093.6	547.3	1,076.5	1,075.6	9
7	811.5	406.2	794.4	793.5	Q	1,022.5	511.8	1,005.5	1,004.5	8
8	926.5	463.8	909.5	908.5	D	894.5	447.7	877.4	876.5	7
9	1,073.6	537.3	1,056.5	1,055.6	F	779.4	390.2	762.4	761.4	6
10	1,201.7	601.3	1,184.6	1,183.6	K	632.4	316.7	615.3	614.4	5
11	1,302.7	651.9	1,285.7	1,284.7	T	504.3		487.3	486.3	4
12	1,417.7	709.4	1,400.7	1,399.7	D	403.2		386.2	385.2	3
13	1,530.8	765.9	1,513.8	1,512.8	L	288.2		271.2		2
14	1,704.9	853.0	1,687.9	1,686.9	R	175.1		158.1		1

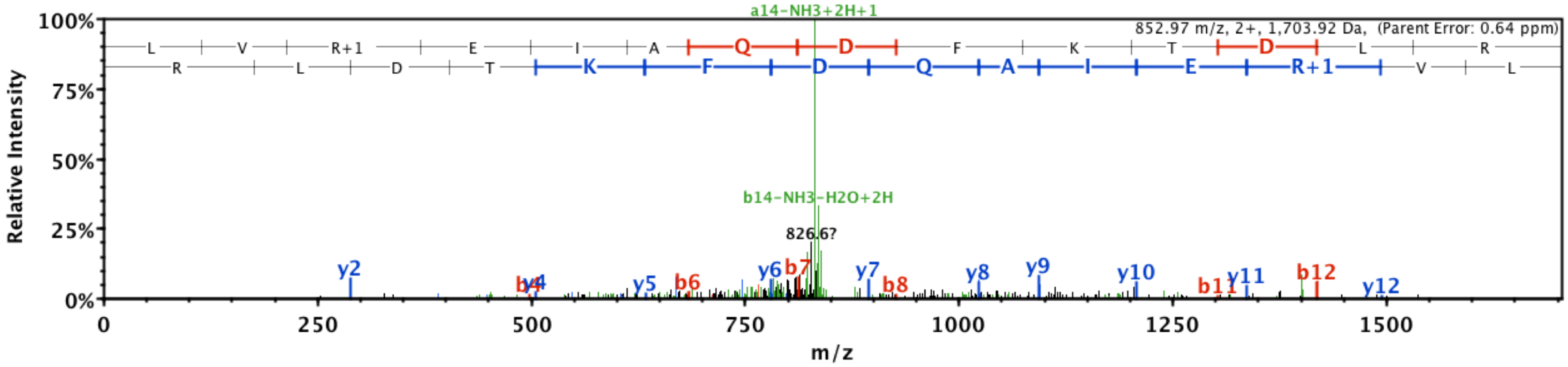

B	B Ions	B+2H	B-NH3	B-H2O	AA	Y Ions	Y+2H	Y-NH3	Y-H2O	Y
1	114.1				L	1,704.9	853.0	1,687.9	1,686.9	14
2	213.2				V	1,591.8	796.4	1,574.8	1,573.8	13
3	370.2	185.6	353.2		R+1	1,492.8	746.9	1,475.7	1,474.8	12
4	499.3	250.1	482.3	481.3	E	1,335.7	668.3	1,318.7	1,317.7	11
5	612.4	306.7	595.3	594.4	I	1,206.6	603.8	1,189.6	1,188.6	10
6	683.4	342.2	666.4	665.4	A	1,093.6	547.3	1,076.5	1,075.6	9
7	811.5	406.2	794.4	793.5	Q	1,022.5	511.8	1,005.5	1,004.5	8
8	926.5	463.8	909.5	908.5	D	894.5	447.7	877.4	876.5	7
9	1,073.6	537.3	1,056.5	1,055.6	F	779.4	390.2	762.4	761.4	6
10	1,201.7	601.3	1,184.6	1,183.6	K	632.4	316.7	615.3	614.4	5
11	1,302.7	651.9	1,285.7	1,284.7	T	504.3		487.3	486.3	4
12	1,417.7	709.4	1,400.7	1,399.7	D	403.2		386.2	385.2	3
13	1,530.8	765.9	1,513.8	1,512.8	L	288.2		271.2		2
14	1,704.9	853.0	1,687.9	1,686.9	R	175.1		158.1		1

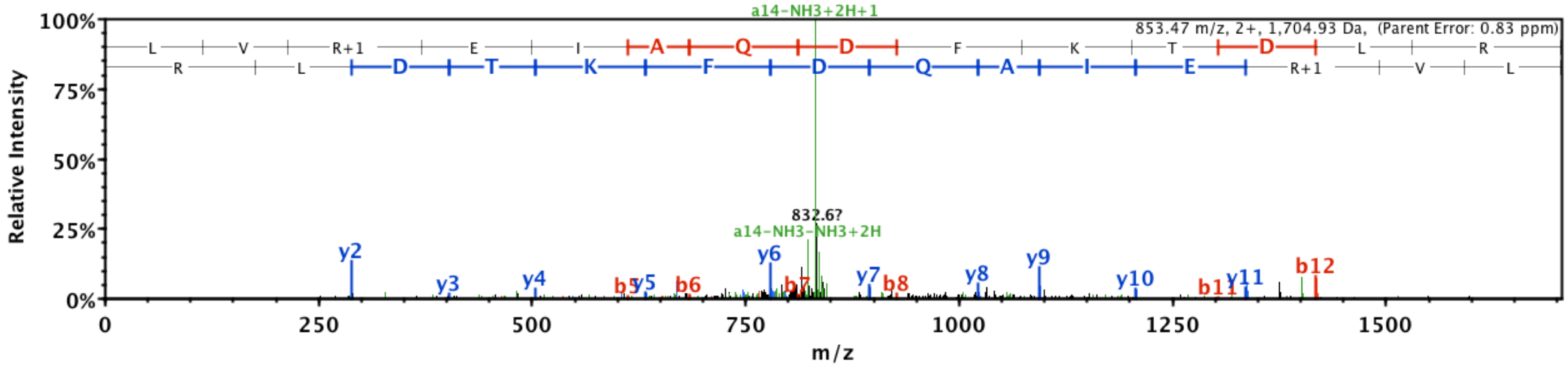

B	B Ions	B+2H	B-NH3	B-H2O	AA	Y Ions	Y+2H	Y-NH3	Y-H2O	Y
1	114.1				L	1,704.9	853.0	1,687.9	1,686.9	14
2	213.2				V	1,591.8	796.4	1,574.8	1,573.8	13
3	370.2	185.6	353.2		R+1	1,492.8	746.9	1,475.7	1,474.8	12
4	499.3	250.1	482.3	481.3	E	1,335.7	668.3	1,318.7	1,317.7	11
5	612.4	306.7	595.3	594.4	I	1,206.6	603.8	1,189.6	1,188.6	10
6	683.4	342.2	666.4	665.4	A	1,093.6	547.3	1,076.5	1,075.6	9
7	811.5	406.2	794.4	793.5	Q	1,022.5	511.8	1,005.5	1,004.5	8
8	926.5	463.8	909.5	908.5	D	894.5	447.7	877.4	876.5	7
9	1,073.6	537.3	1,056.5	1,055.6	F	779.4	390.2	762.4	761.4	6
10	1,201.7	601.3	1,184.6	1,183.6	K	632.4	316.7	615.3	614.4	5
11	1,302.7	651.9	1,285.7	1,284.7	T	504.3		487.3	486.3	4
12	1,417.7	709.4	1,400.7	1,399.7	D	403.2		386.2	385.2	3
13	1,530.8	765.9	1,513.8	1,512.8	L	288.2		271.2		2
14	1,704.9	853.0	1,687.9	1,686.9	R	175.1		158.1		1

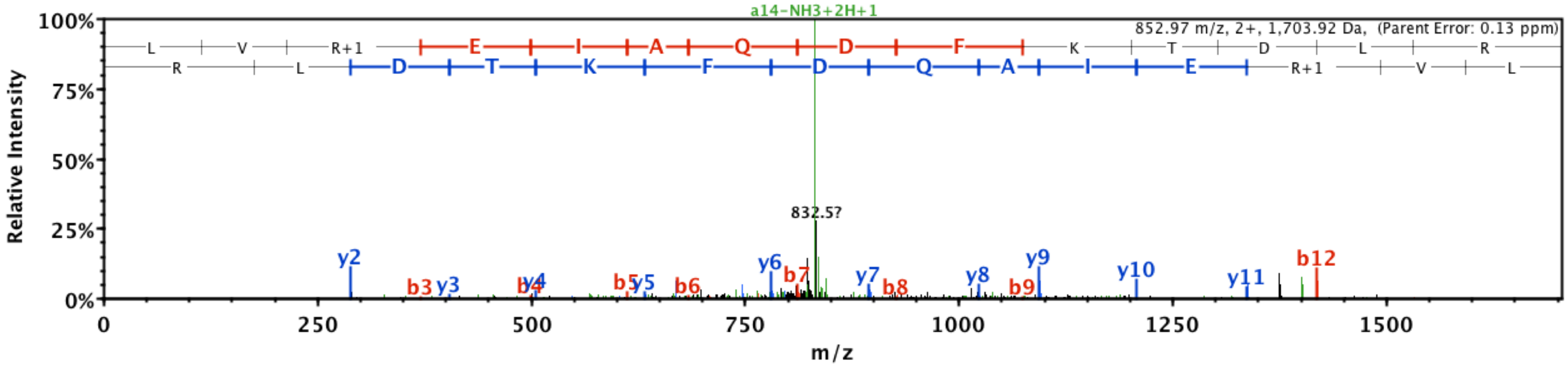

B	B Ions	B+2H	B-NH3	B-H2O	AA	Y Ions	Y+2H	Y-NH3	Y-H2O	Y
1	114.1				L	1,704.9	853.0	1,687.9	1,686.9	14
2	213.2				V	1,591.8	796.4	1,574.8	1,573.8	13
3	370.2	185.6	353.2		R+1	1,492.8	746.9	1,475.7	1,474.8	12
4	499.3	250.1	482.3	481.3	E	1,335.7	668.3	1,318.7	1,317.7	11
5	612.4	306.7	595.3	594.4	I	1,206.6	603.8	1,189.6	1,188.6	10
6	683.4	342.2	666.4	665.4	A	1,093.6	547.3	1,076.5	1,075.6	9
7	811.5	406.2	794.4	793.5	Q	1,022.5	511.8	1,005.5	1,004.5	8
8	926.5	463.8	909.5	908.5	D	894.5	447.7	877.4	876.5	7
9	1,073.6	537.3	1,056.5	1,055.6	F	779.4	390.2	762.4	761.4	6
10	1,201.7	601.3	1,184.6	1,183.6	K	632.4	316.7	615.3	614.4	5
11	1,302.7	651.9	1,285.7	1,284.7	T	504.3		487.3	486.3	4
12	1,417.7	709.4	1,400.7	1,399.7	D	403.2		386.2	385.2	3
13	1,530.8	765.9	1,513.8	1,512.8	L	288.2		271.2		2
14	1,704.9	853.0	1,687.9	1,686.9	R	175.1		158.1		1

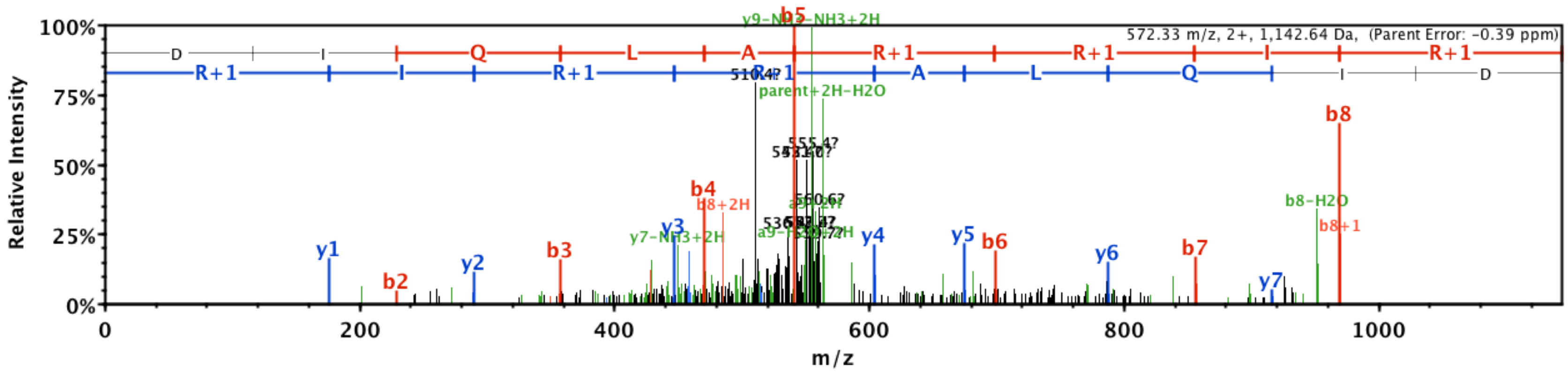

B	B Ions	B+2H	B-NH3	B-H2O	AA	Y Ions	Y+2H	Y-NH3	Y-H2O	Y
1	116.0			98.0	D	1,143.6	572.3	1,126.6	1,125.6	9
2	229.1			211.1	I	1,028.6	514.8	1,011.6		8
3	357.2		340.2	339.2	Q	915.5	458.3	898.5		7
4	470.3		453.2	452.3	L	787.5	394.2	770.5		6
5	541.3		524.3	523.3	A	674.4	337.7	657.4		5
6	698.4	349.7	681.4	680.4	R+1	603.4	302.2	586.3		4
7	855.5	428.2	838.4	837.5	R+1	446.3	223.6	429.2		3
8	968.6	484.8	951.5	950.5	I	289.2		272.2		2
9	1,143.6	572.3	1,126.6	1,125.6	R+1	176.1		159.1		1

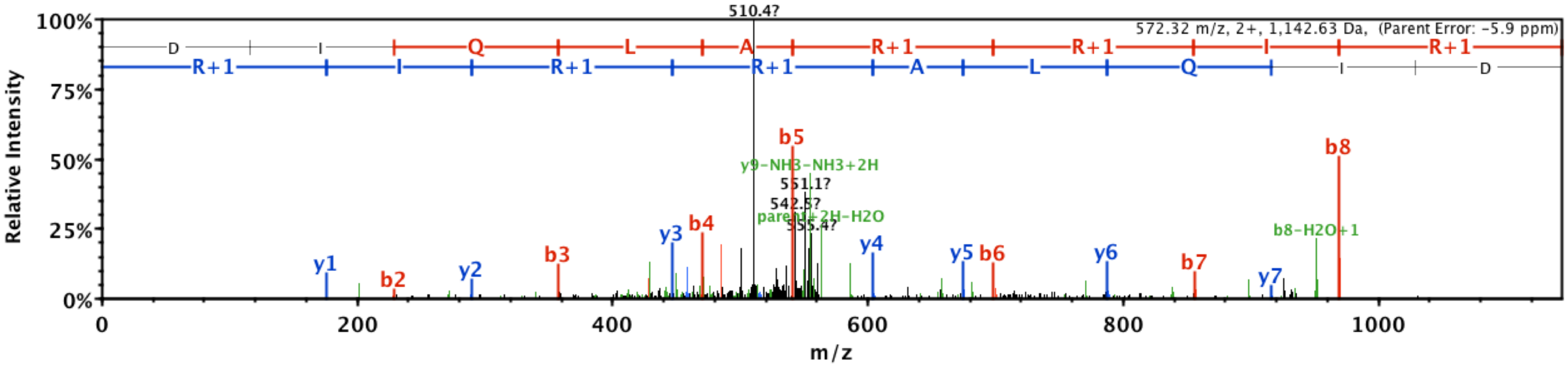

B	B Ions	B+2H	B-NH3	B-H2O	AA	Y Ions	Y+2H	Y-NH3	Y-H2O	Y
1	116.0			98.0	D	1,143.6	572.3	1,126.6	1,125.6	9
2	229.1			211.1	I	1,028.6	514.8	1,011.6		8
3	357.2		340.2	339.2	Q	915.5	458.3	898.5		7
4	470.3		453.2	452.3	L	787.5	394.2	770.5		6
5	541.3		524.3	523.3	A	674.4	337.7	657.4		5
6	698.4	349.7	681.4	680.4	R+1	603.4	302.2	586.3		4
7	855.5	428.2	838.4	837.5	R+1	446.3	223.6	429.2		3
8	968.6	484.8	951.5	950.5	I	289.2		272.2		2
9	1,143.6	572.3	1,126.6	1,125.6	R+1	176.1		159.1		1

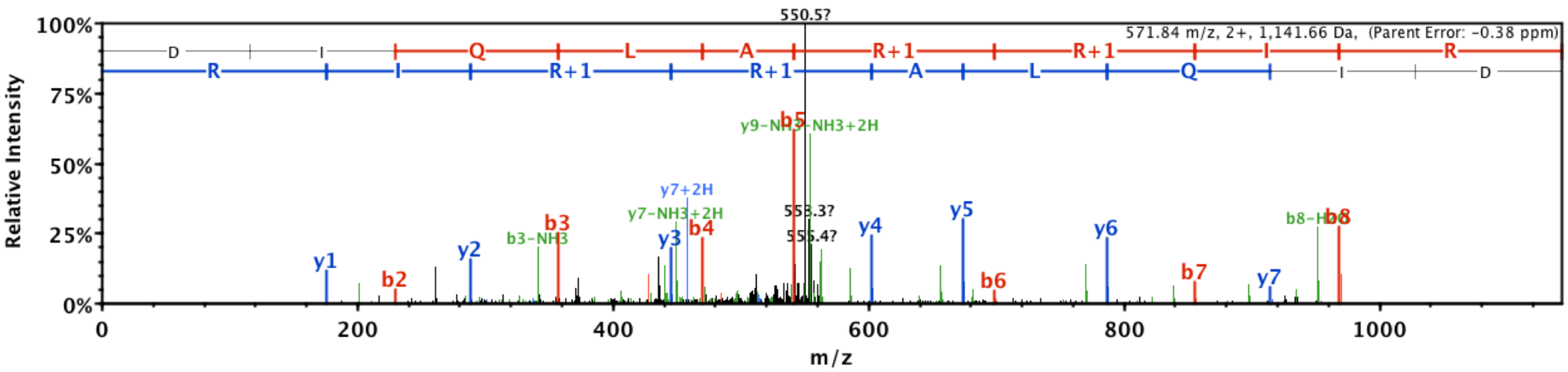

B	B Ions	B+2H	B-NH3	B-H2O	AA	Y Ions	Y+2H	Y-NH3	Y-H2O	Y
1	116.0			98.0	D	1,142.7	571.8	1,125.6	1,124.7	9
2	229.1			211.1	I	1,027.6	514.3	1,010.6		8
3	357.2		340.2	339.2	Q	914.6	457.8	897.5		7
4	470.3		453.2	452.3	L	786.5	393.8	769.5		6
5	541.3		524.3	523.3	A	673.4	337.2	656.4		5
6	698.4	349.7	681.4	680.4	R+1	602.4	301.7	585.3		4
7	855.5	428.2	838.4	837.5	R+1	445.3	223.1	428.3		3
8	968.6	484.8	951.5	950.5	I	288.2		271.2		2
9	1,142.7	571.8	1,125.6	1,124.7	R	175.1		158.1		1

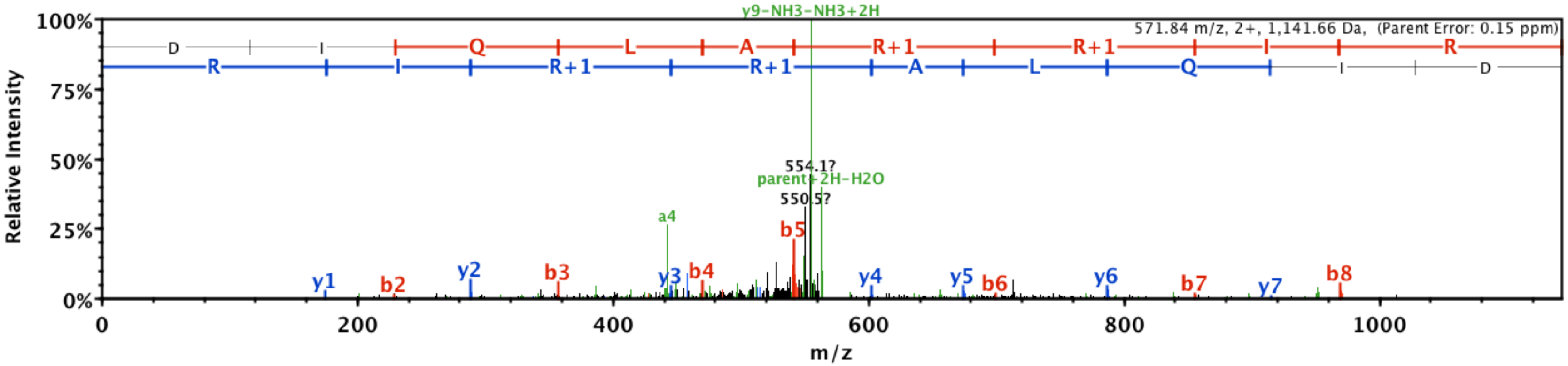

B	B Ions	B+2H	B-NH3	B-H2O	AA	Y Ions	Y+2H	Y-NH3	Y-H2O	Y
1	116.0			98.0	D	1,142.7	571.8	1,125.6	1,124.7	9
2	229.1			211.1	I	1,027.6	514.3	1,010.6		8
3	357.2		340.2	339.2	Q	914.6	457.8	897.5		7
4	470.3		453.2	452.3	L	786.5	393.8	769.5		6
5	541.3		524.3	523.3	A	673.4	337.2	656.4		5
6	698.4	349.7	681.4	680.4	R+1	602.4	301.7	585.3		4
7	855.5	428.2	838.4	837.5	R+1	445.3	223.1	428.3		3
8	968.6	484.8	951.5	950.5	I	288.2		271.2		2
9	1,142.7	571.8	1,125.6	1,124.7	R	175.1		158.1		1

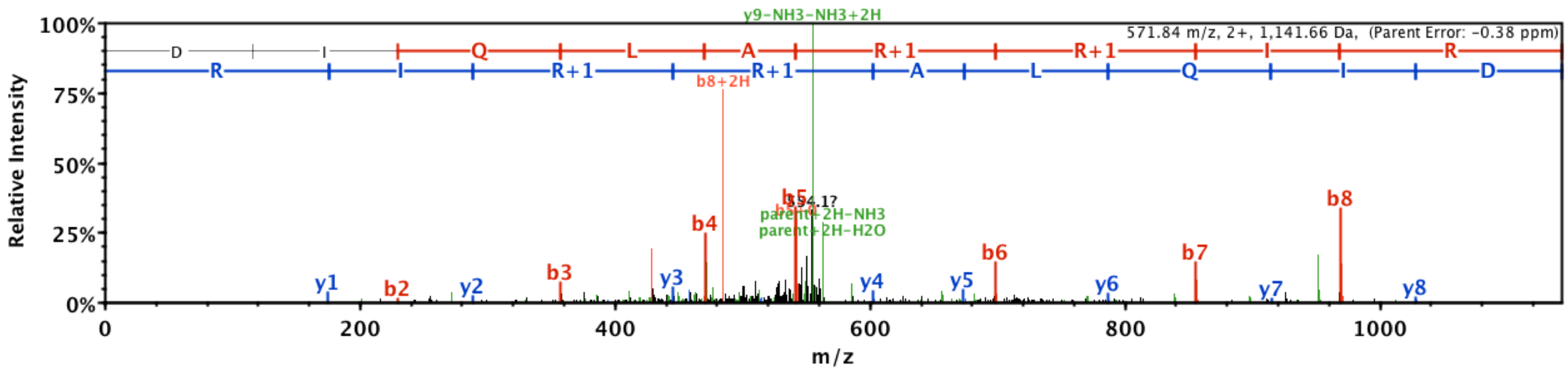

B	B Ions	B+2H	B-NH3	B-H2O	AA	Y Ions	Y+2H	Y-NH3	Y-H2O	Y
1	116.0			98.0	D	1,142.7	571.8	1,125.6	1,124.7	9
2	229.1			211.1	I	1,027.6	514.3	1,010.6		8
3	357.2		340.2	339.2	Q	914.6	457.8	897.5		7
4	470.3		453.2	452.3	L	786.5	393.8	769.5		6
5	541.3		524.3	523.3	A	673.4	337.2	656.4		5
6	698.4	349.7	681.4	680.4	R+1	602.4	301.7	585.3		4
7	855.5	428.2	838.4	837.5	R+1	445.3	223.1	428.3		3
8	968.6	484.8	951.5	950.5	I	288.2		271.2		2
9	1,142.7	571.8	1,125.6	1,124.7	R	175.1		158.1		1

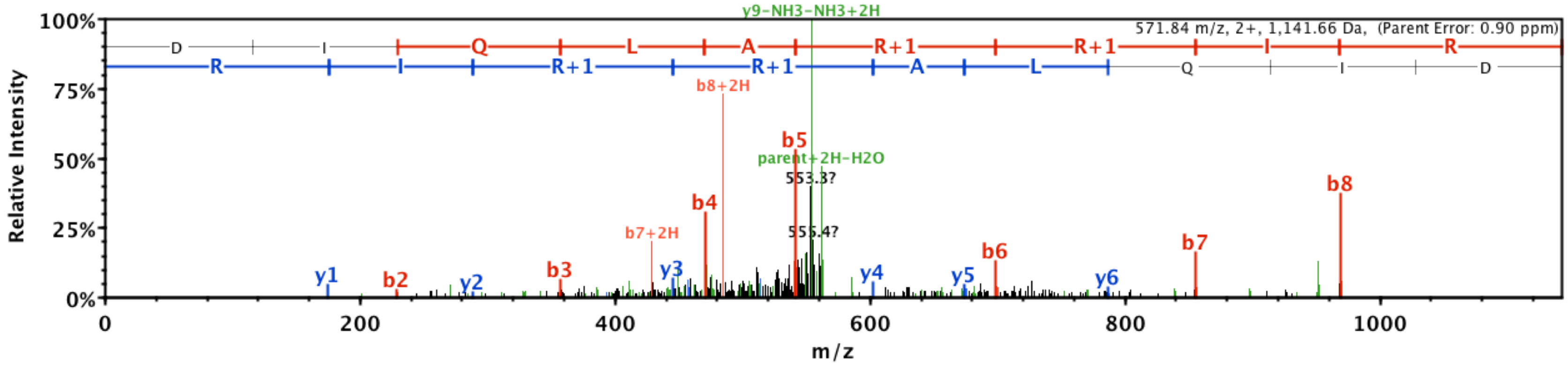

B	B Ions	B+2H	B-NH3	B-H2O	AA	Y Ions	Y+2H	Y-NH3	Y-H2O	Y
1	116.0			98.0	D	1,142.7	571.8	1,125.6	1,124.7	9
2	229.1			211.1	I	1,027.6	514.3	1,010.6		8
3	357.2		340.2	339.2	Q	914.6	457.8	897.5		7
4	470.3		453.2	452.3	L	786.5	393.8	769.5		6
5	541.3		524.3	523.3	A	673.4	337.2	656.4		5
6	698.4	349.7	681.4	680.4	R+1	602.4	301.7	585.3		4
7	855.5	428.2	838.4	837.5	R+1	445.3	223.1	428.3		3
8	968.6	484.8	951.5	950.5	I	288.2		271.2		2
9	1,142.7	571.8	1,125.6	1,124.7	R	175.1		158.1		1

LP9

Derived from human lysozyme

Uniprot ID: B2R4C5

Citrullinated peptides identified when incubated with PPAD

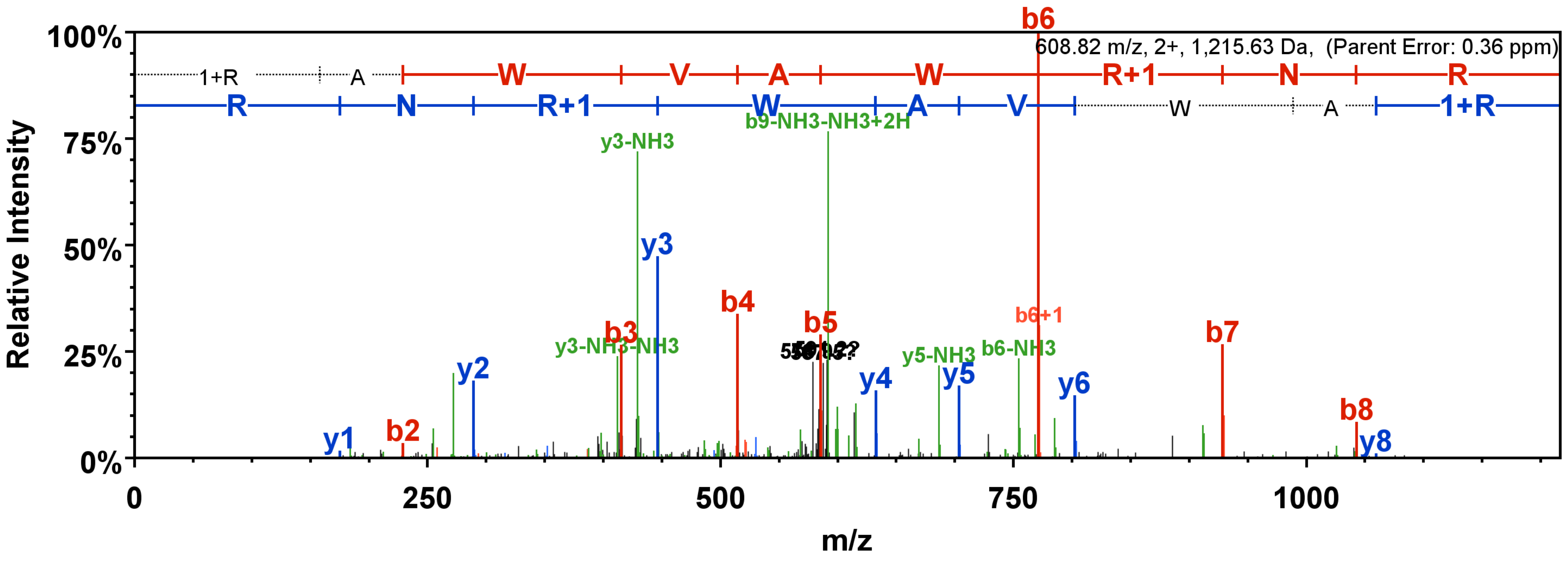

B	B Ions	B+2H	B-NH3	B-H2O	AA	Y Ions	Y+2H	Y-NH3	Y-H2O	Y
1	158.1	79.5	141.1		R+1	1,216.6	608.8	1,199.6		9
2	229.1	115.1	212.1		A	1,059.5	530.3	1,042.5		8
3	415.2	208.1	398.2		W	988.5	494.8	971.5		7
4	514.3	257.6	497.3		V	802.4	401.7	785.4		6
5	585.3	293.2	568.3		A	703.4	352.2	686.3		5
6	771.4	386.2	754.4		W	632.3	316.7	615.3		4
7	928.5	464.7	911.5		R+1	446.2	223.6	429.2		3
8	1,042.5	521.8	1,025.5		N	289.2		272.1		2
9	1,216.6	608.8	1,199.6		R	175.1		158.1		1

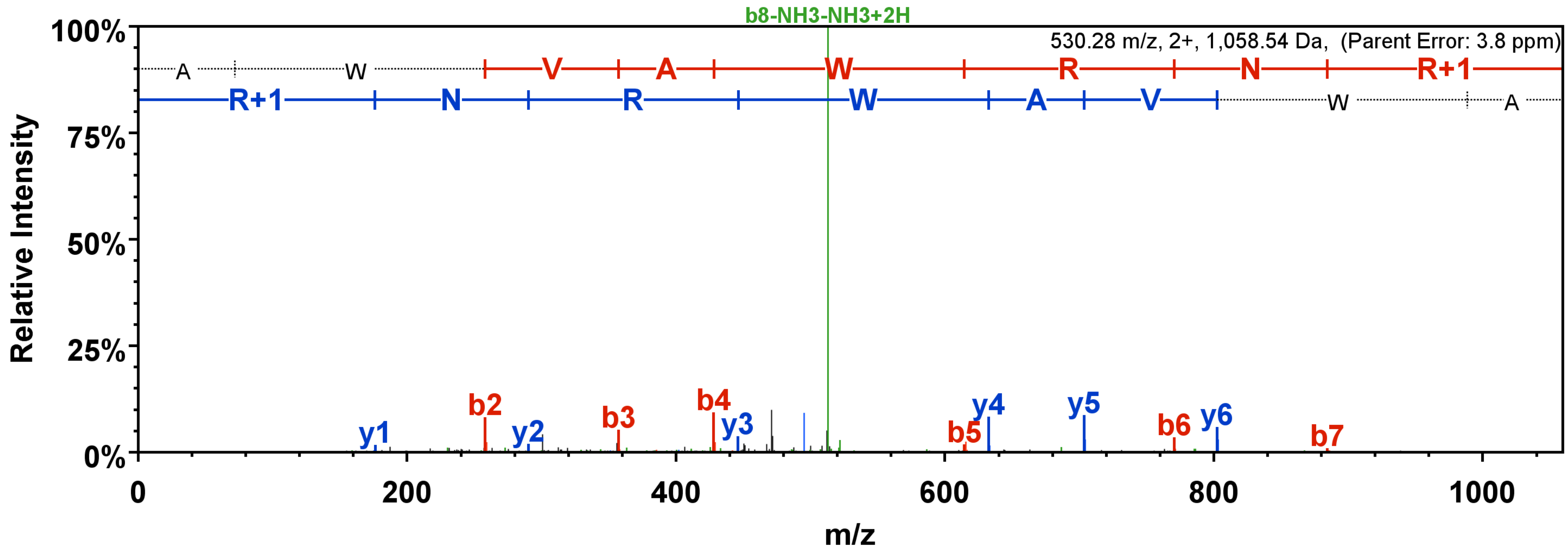

B	B Ions	B+2H	B-NH3	B-H2O	AA	Y Ions	Y+2H	Y-NH3	Y-H2O	Y
1	72.0				A	1,059.5	530.3	1,042.5		8
2	258.1				W	988.5	494.8	971.5		7
3	357.2				V	802.4	401.7	785.4		6
4	428.2				A	703.4	352.2	686.3		5
5	614.3				W	632.3	316.7	615.3		4
6	770.4	385.7	753.4		R	446.2	223.6	429.2		3
7	884.5	442.7	867.4		N	290.1		273.1		2
8	1,059.5	530.3	1,042.5		R+1	176.1		159.1		1

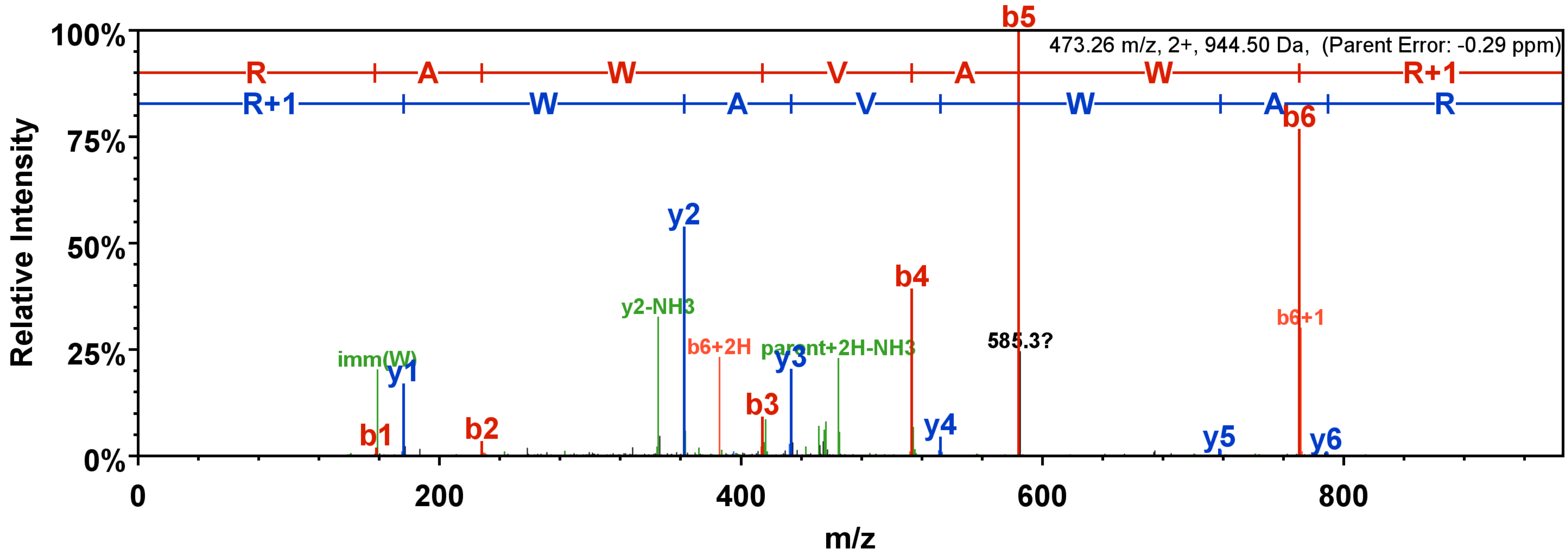

B	B Ions	B+2H	B-NH3	B-H2O	AA	Y Ions	Y+2H	Y-NH3	Y-H2O	Y
1	157.1	79.1	140.1		R	945.5	473.3	928.5		7
2	228.1	114.6	211.1		A	789.4	395.2	772.4		6
3	414.2	207.6	397.2		W	718.4		701.3		5
4	513.3	257.2	496.3		V	532.3		515.3		4
5	584.3	292.7	567.3		A	433.2		416.2		3
6	770.4	385.7	753.4		W	362.2		345.2		2
7	945.5	473.3	928.5		R+1	176.1		159.1		1
